# Super-enhancer hijacking *LINC01977* promotes malignancy of early-stage lung adenocarcinoma addicted to the canonical TGF-β/SMAD3 pathway

**DOI:** 10.1186/s13045-022-01331-2

**Published:** 2022-08-18

**Authors:** Te Zhang, Wenjie Xia, Xuming Song, Qixing Mao, Xing Huang, Bing Chen, Yingkuan Liang, Hui Wang, Yuzhong Chen, Xinnian Yu, Zeyu Zhang, Wenmin Yang, Lin Xu, Gaochao Dong, Feng Jiang

**Affiliations:** 1 Department of Thoracic Surgery, Affiliated Cancer Hospital of Nanjing Medical University and Jiangsu Cancer Hospital and Jiangsu Institute of Cancer Research, 42 Baiziting Road, Xuanwu District, Nanjing, 210009 China; 2 Jiangsu Key Laboratory of Molecular and Translational Cancer Research, 42 Baiziting Road, Xuanwu District, Nanjing, 210009 China; 3 Collaborative Innovation Center for Cancer Personalized Medicine, Nanjing Medical University, 101 Longmian Avenue, Jiangning District, Nanjing, 211166 China; 4 Department of Pathology, Affiliated Cancer Hospital of Nanjing Medical University and Jiangsu Cancer Hospital and Jiangsu Institute of Cancer Research, 42 Baiziting Road, Xuanwu District, Nanjing, 210009 China; 5 Department of Thoracic Surgery, The First Affiliated Hospital of Soochow University, Suzhou, 215031 China; 6 Department of Oncology, Affiliated Cancer Hospital of Nanjing Medical University and Jiangsu Cancer Hospital and Jiangsu Institute of Cancer Research, 42 Baiziting Road, Xuanwu District, Nanjing, 210009 China

**Keywords:** Super-enhancer, *LINC01977*, TGF-β/SMAD3, Lung adenocarcinoma, Tumor-associated macrophages, Malignancy

## Abstract

**Background:**

Lung adenocarcinoma (LUAD) is the leading cause of death worldwide. However, the roles of long noncoding RNAs (lncRNAs) hijacked by super-enhancers (SEs), vital regulatory elements of the epigenome, remain elusive in the progression of LUAD metastasis.

**Methods:**

SE-associated lncRNA microarrays were used to identify the dysregulated lncRNAs in LUAD. ChIP-seq, Hi-C data analysis, and luciferase reporter assays were utilized to confirm the hijacking of *LINC01977* by SE. The functions and mechanisms of *LINC01977* in LUAD were explored by a series of in vitro and in vivo assays.

**Results:**

We found that *LINC01977*, a cancer-testis lncRNA, was hijacked by SE, which promoted proliferation and invasion both in vitro and in vivo. *LINC01977* interacted with SMAD3 to induce its nuclear transport, which facilitated the interaction between SMAD3 and CBP/P300, thereby regulating the downstream target gene ZEB1. Additionally, SMAD3 up-regulated *LINC09177* transcription by simultaneously binding the promoter and SE, which was induced by the infiltration of M2-like tumor-associated macrophages (TAM2), subsequently activating the TGF-β/SMAD3 pathway. Moreover, *LINC01977* expression was positively correlated with TAM2 infiltration and SMAD3 expression, especially in early-stage LUAD. Higher chromatin accessibility in the SE region of *LINC01977* was observed with high expression of TGF-β. Early-stage LUAD patients with high *LIN01977* expression had a shorter disease-free survival.

**Conclusions:**

TAM2 infiltration induced a rich TGF-β microenvironment, activating SMAD3 to bind the promoter and the SE of *LINC01977*, which up-regulated *LINC01977* expression. *LINC01977* also promoted malignancy via the canonical TGF-β/SMAD3 pathway. *LINC01977* hijacked by SE could be a valuable therapeutic target, especially for the treatment of early-stage LUAD.

**Supplementary Information:**

The online version contains supplementary material available at 10.1186/s13045-022-01331-2.

## Background

Lung cancer is a major cause of cancer-associated death worldwide, of which the most prevalent histological subtype is lung adenocarcinoma (LUAD), accounting for more than 40% of all cases [1]. Resection with or without adjuvant chemotherapy and/or adjuvant radiotherapy is the standard treatment for patients with early-stage LUAD [2]. However, many of these patients will relapse with 5 years, from 20% of those with stage I disease to 50% of those with stage III, thereby contributing to the dismal prognosis of patients [3]. Although the detection of circulating tumor DNA (ctDNA) has been proved to be an effective method for identifying patients who are at high risk of disease recurrence on the basis of minimal residual disease (MRD), it is important to reveal the mechanism responsible for the interpreted relapse of early-stage LUAD patients [4].

A better understanding of the cancer genome and driving events has revolutionized the treatment of patients who possess genomic driver alterations that can be targeted with matched drugs [5]. Targeted therapies that attack EGFR mutations, as well as ALK- and ROS1-fusion events, have dramatically improved the survival of LUAD patients [6]. However, to date, cancer genes exclusively mutated in the context of metastatic disease have not been identified [7, 8]. Dynamic epigenetic alterations, such as enhancer reprogramming and super-enhancer (SE) hijacking events, are now being implicated as the key drivers of metastasis [9, 10]. It is not well understood whether SE is the key driver of LUAD metastasis, especially in patients with early-stage disease.

Super-enhancers, which refer to large genomic domains with enriched enhancer activity, recruit core regulatory circuitry transcription factors (TFs) to mediate transcriptional dysregulation in human cancers [11]. In a previous study, our group demonstrated that ELF3, EHF, and TGIF1 formed an SE-dependent transcriptional regulatory network to perturb transcriptional events in LUAD [12]. SEs also generate enhancer RNAs (eRNAs) or hijack promoters to up-regulate non-coding RNAs, namely super-enhancer-associated long non-coding RNAs (SE-lncRNAs). It has been reported that TP63 and SOX2 cooperatively regulate *lncRNA CCAT1* expression by activating its SE and promoter in squamous cell carcinoma [13]. *LINC01503*, which is regulated by SE and TP63, activates ERK and AKT pathways to promote the progression of squamous cell carcinoma [14]. Nevertheless, the functions of lncRNAs hijacked by SEs in LUAD are still unknown.

TGF-β, a secreted cytokine, activates SMAD2 and SMAD3, which combine with SMAD4 to form a trimeric complex that translocates into the nucleus [15]. The canonical TGF-β/SMAD3 pathway is vital for the progression of malignancy in LUAD patients [16]. Single nucleotide polymorphisms (SNPs) in SMAD3 are highly predictive of the outcome of LUAD patients after gefitinib treatment [17]. Moreover, TGF-β has been reported to promote programmed death ligand 1 (PD-L1) expression in TAMs, while a phase 3 clinical trial of anti-PD-L1/TGF-β trap (M7824) for NSCLC failed to show significant therapeutic benefits compared with PD-1 inhibitor (pembrolizumab), and thus, the clinical trial was terminated [18]. M2-like tumor-associated macrophages (TAM2) in the tumor microenvironment are the predominant sources of TGF-β [19]. However, the mechanism by which cancer cells response and adaptive behaviors to TAM2 high infiltration in the extrinsic TME whether dependent on the intrinsic characteristics referring to the activity of SEs are still unknown.

Herein, we investigate dysregulated SE-associated lncRNAs in LUAD and identified that the up-regulated *LINC01977* was hijacked by SE, which promoted the malignant phenotype both in vitro and in vivo. Mechanistically, *LINC01977* participated in the TGF-β/SMAD3 pathway by promoting the nuclear accumulation of SMAD3 and enhancing the establishment of the SMAD3/CBP/P300 complex, resulting in the epigenetic activation of ZEB1, the central switch of the epithelial–mesenchymal transition (EMT). Equally important, *LINC01977* expression was positively correlated with TAM2 infiltration and SMAD3 expression. LUAD patients with high *LINC01977* expression had a shorter disease-free survival, especially in early-stage. These results highlight the potential of SE-hijacked lncRNAs as prognostic biomarkers and therapeutic targets for early-stage LUAD.

## Methods

### Cell lines, PBMC isolation, and cell sorting

LUAD cell lines (A549, A427, PC-9, H1299, SW1573, H1975, H358), the human bronchial epithelial cell line (HBE), and the 293T cell line were obtained from the cell bank of the Shanghai Institute of Cell Biology (Chinese Academy of Medical Science, Shanghai, China) and maintained in RPMI-1640 medium (Gibco, USA) or Dulbecco’s modified Eagle’s medium (DMEM; Gibco) supplemented with 10% fetal bovine serum (FBS, Gibco) and 1% penicillin–streptomycin (Gibco) at 37 °C with 5% CO_2_. Based on short tandem repeat (STR) profiling by the manufacturers, no cell lines used in this study were found in the database of commonly misidentified cell lines. All cell lines were authenticated and tested routinely for their authenticity and found to be free of mycoplasma contamination. Gene tracks and mutation status correlation analysis of A549, PC-9, and H1299 are listed in Additional file 1: Figure S1H-L.

Blood was collected in EDTA-coated tubes (BD Biosciences, USA) and centrifuged at 1200 g for 15 min to separate the plasma. The mononuclear cells were purified by density gradient centrifugation using Ficoll (Ficoll-Paque PLUS; GE Healthcare, USA) according to the manufacturer’s instructions.

PBMCs were stained with monoclonal antibodies against CD3 (BioLegend, USA), CD4 (BioLegend), and CD8 (BioLegend). Thereafter, the cells were washed and CD3^+^ T cells, CD4^+^ T cells, and CD8^+^ T cells were sorted in a biosafety cabinet (Baker Hood, USA) using the FACS Aria cell sorter (BD Biosciences, USA) at 70 pounds per square inch. All antibody information is provided in Additional file 2: Table S9.

### Human tissue specimens

LUAD tissues and paired adjacent normal tissues were collected from patients at the Affiliated Cancer Hospital of Nanjing Medical University (Jiangsu Cancer Hospital, Jiangsu Institute of Cancer Research, Nanjing, China). All specimens were obtained from patients who underwent surgical resection of LUAD and reviewed by experienced pathologists at the Affiliated Cancer Hospital of Nanjing Medical University. All samples were obtained from biobank of Jiangsu Cancer Hospital (Jiangsu Institute of Cancer Research & The Affiliated Cancer Hospital of Nanjing Medical University). All patients had signed informed consent for donating their samples.

The specimens were collected, immediately frozen in liquid nitrogen, and stored at -80 °C until use. Five pairs of LUAD tissues and adjacent normal tissues were used for SE-associated lncRNA microarrays. Forty pairs of LUAD tissues and adjacent normal tissues were used for RNA extraction for qRT-PCR. Ninety-eight pairs of LUAD tissues and adjacent normal tissues were used for the construction of tissue microarrays (TMAs). One hundred eighty-six LUAD tumor tissues were used for RNA extraction for qRT-PCR and fixation in formalin overnight, followed by embedding in paraffin by a routine procedure. Written informed consent was obtained from all the subjects. The study protocol was approved by the ethics committee of Affiliated Cancer Hospital of Nanjing Medical University, Nanjing, China. All clinicopathological information is provided in Additional file 2: Tables S1, S2, and S5.

### Reagents and tagged plasmid construction

TGF-β, SGC-CBP30, JQ1, SB431542, and SIS3 were purchased from ApexBio (USA). The N-terminal HA tag was purchased from Novus Biologicals (USA). The N-terminal HA-tagged expression vectors (for overexpression in mammalian cells) for SMAD3 and truncated mutants were procured from BioWorld (China). The Flag-tagged expression vectors for *LINC01977* and truncated mutants were also procured from BioWorld. All reagent information is provided in Additional file 2: Table S9, and cloning primer information is provided in Additional file 2: Table S7.

### RNA interference

Cell transfection was performed according to the manufacturer’s instructions. Antisense oligonucleotide (ASO) were purchased from RiboBio (China). The constructs were verified by sequencing and validated by qRT-PCR. All sequence information is listed in Additional file 2: Table S7.

### Genome editing with CRISPR-Cas9

CRISPR-Cas9 gene editing was used to knock out SMAD3, CREBBP (CBP) and EP300 (P300). In brief, gRNAs were designed using the online tool CRISPick (https://portals.broadinstitute.org/gppx/crispick/public). LUAD cell lines were transfected using lipofectamine CRISPRMAX with Cas9 protein. Genomic DNA was extracted using the Genomic DNA extraction kit purchased from TIANGEN (China). The targeted region was PCR amplified. gRNA oligonucleotides and corresponding primers are listed in Additional file 2: Table S7.

### RNAscope ISH assay

RNAscope ISH assays were performed using the RNAscope 2.5 High-Definition Red Reagent Assay Kit (Advanced Cell Diagnostics, USA) to detect *LINC01977*. ISH was performed according to the manufacturer’s instructions. In brief, 98 pairs of LUAD 4-μm-thick TMA sections were mounted onto SuperFrostPlus slides, dried, and stored at -80 °C. The sections were pretreated with hydrogen peroxide and incubated for 15 min, followed by incubation with Protease Plus reagent for 30 min at 4 °C. Next, the sections were hybridized with the corresponding target probe for 2 h at 4 °C, followed by a series of amplification and washing steps. Chromogenic signal detection was performed by incubation with DAB for 20 min at room temperature. The sections were counterstained with Mayer’s hematoxylin solution (to stain nuclei) and mounted with EcoMount reagent. According to the manufacturer’s instructions, each dot represented a single mRNA molecule in these sections. All reagent and kit information is provided in Additional file 2: Table S9.

### Immunohistochemistry (IHC)

Serial 4-μm-thick paraffin-embedded sections were dewaxed and rehydrated. Antigen retrieval was performed in a pressure cooker for 5 min in 10 mM Tris containing 1 mM EDTA (pH 9.0). The sections were incubated with specific antibodies against SMAD3 Ser423/425, CBP, ZEB1 E-cadherin, Ki-67, PCNA, and cleaved caspase 3 at 4 °C, and immunodetection was performed with DAB on the following day. All antibody information is provided in Additional file 2: Table S9.

### Fluorescence in situ hybridization (FISH) assay and immunofluorescence staining

FISH assays were carried out with the lncRNA FISH Kit (RiboBio). In brief, the cells were fixed and permeabilized in PBS containing 0.5% Triton X-100. FISH probes were designed by RiboBio. Hybridization was carried out overnight in a humidified chamber at 37 °C in the dark. All images were obtained with a CarlZeiss LSM710 confocal microscope (Germany). 4’,6-Diamidino-2-phenylindole and Cy3 channels were used to detect the signals. U6 and 18S were used as the nuclear and cytoplasmic markers, respectively. Immunofluorescence staining was performed according to the manufacturer’s instructions. All antibody information is provided in Additional file 2: Table S9.

### Colony formation assay

Colony formation assays were used to monitor cellular clonogenic potential. In brief, following transfection, treated cells (1.7 × 10^3^) were plated in 6-well plates in triplicate. After 14 days of incubation, the cells were washed twice with PBS, fixed with methanol for 10 min, and stained with 0.1% crystal violet solution for 10 min, followed by analysis.

### 5-Ethynyl-20-deoxyuridine (EdU) assay

The EdU DNA Proliferation Kit (KeyGene, China) was used to monitor cell proliferation according to the manufacturer’s instructions. In brief, LUAD cells were cultured in 96-well plates in complete media until 80% confluent and treated with 50 μM EdU for 6 h, followed by analysis.

### Cell cycle analysis using flow cytometry

For cell cycle distribution analysis, LUAD cells (1.0 × 10^5^) were fixed in ice-cold 70% ethanol before staining with propidium iodide (PI) and analyzed with a FAC-Scan flow cytometer (BD Biosciences, USA). Data were analyzed using FlowJo software (FlowJo LLC, USA).

### RNA extraction, reverse transcription, and quantitative PCR (qPCR)

Total RNA from cells and fresh-frozen tissues were extracted using TRIzol reagent (Invitrogen, USA) according to the manufacturer’s protocol. For FFPE samples, RNA was extracted from three 5-μM-thick LUAD FFPE specimens with the RNeasy FFPE Kit (Qiagen, Germany). For qRT-PCR, cDNA was synthesized using the PrimeScript RT Reagent Kit (Takara, China). The reaction was carried out for 15 min at 37 °C, 5 min at 85 °C, and then 4 °C until further use.

For qPCR, the expression of genes was measured with the PowerUp SYBR Green Master Mix (Thermo Fisher Scientific, USA) in triplicate using the Applied Biosystems Prism 7500 Fast Sequence Detection System (USA). GAPDH, ACTB, and snRNA U6 were used as the internal controls. The relative level of each target RNA was calculated using the 2^△△Ct^ method and normalized to ACTB. All primer sequence information is listed in Additional file 2: Table S8, and all reagent and kit information is listed in Additional file 2: Table S9.

### Dual-luciferase reporter assay

The wild-type ZEB1 promoter (− 1 to − 2000) and mutants were synthesized and cloned into the pGL3-basic luciferase reporter plasmid (RealGene, China). The *LINC01977* promoter (− 1 to − 2000) and the associated SE region (E1–E6) were also synthesized and cloned into the pGL3-basic luciferase reporter plasmid (RealGene). The cells were transfected with a mixture of Renilla luciferase and the indicated luciferase reporter. After transfection for 48 h, the cells were harvested, and luciferase activity was evaluated using the Dual-Luciferase Assay Kit (Promega, USA) and GLOMAX 96 microplate luminometer (Promega). The relative luciferase activity was normalized to Renilla luciferase activity. DNA sequence and cloning primer information is provided in Additional file 2: Table S7.

### Western blotting

Western blotting was performed as described previously [12]. In brief, cell lysates were separated by 10% sodium dodecyl sulfate polyacrylamide gel electrophoresis (SDS-PAGE), and proteins were transferred to polyvinylidene difluoride (PVDF) membranes (Merck Millipore, USA). The membranes were incubated with primary antibodies at 4 °C overnight, followed by incubation with corresponding secondary antibodies. Target proteins were detected and qualified using a grayscale ratio with the Odyssey CLx Imaging System (LI-COR, USA). All antibody information is provided in Additional file 2: Table S9.

### Three-dimensional tumor spheroid culture

LUAD cells were cultured as hanging drops for 24 h, which allowed the formation of spheroids, with each spheroid containing approximately 5 × 10^3^ cells. The cells were seeded into gelatin-functionalized non-adherent U-bottom 96-well plates (Corning, USA). The cells grew into spheroids for 5 days at 37 °C with 5% CO_2_ and images were acquired with a microscope (Olympus, Japan).

### Transwell migration and invasion assays

For migration assay, the cells were plated into upper chambers of Transwell units (pore size, 8 μm; Corning). For invasion assays, the cells were added into Matrigel (BD Biosciences, USA)-coated upper chambers of Transwell units (8 μm; Corning, USA). The lower chamber was filled with RPMI-1640 medium supplemented with 10% FBS. The Transwell units were incubated for 24–48 h and stained with 0.1% crystal violet for 10 min. The number of migrated or invaded cells on the lower surface of the membrane was determined with a microscope (Olympus).

### Wound healing assay

LUAD cells (4 × 10^5^) were seeded in 12-well plates and transfected. At 24 h after transfection, the cell monolayers were wounded by scratching with a sterile 200-μl micropipette tip. The cells were imaged with a phase-contrast microscope immediately and 24 h after wounding. The migration area was determined by Adobe Photoshop software (USA). The assay was performed three independent times.

### RNA pull-down and RIP assay

RNA was transcribed in vitro using the RNAmax-T7 transcription Kit (RiboBio) and biotinylated using the Pierce RNA 3’ End Desthiobiotinylation Kit (Thermo Fisher Scientific) according to the manufacturer’s instructions. The cells were lysed using Pierce IP lysis buffer (Thermo Fisher Scientific). RNA pull-down assays were performed using the Pierce Magnetic RNA–Protein Pull-Down Kit (Thermo Fisher Scientific). Next, desthiobiotinylated RNA was captured with streptavidin magnetic beads and incubated with whole cell lysates at 4 °C for 6 h, followed by washing and elution of the RBP complex. The eluted proteins were subjected to western blotting and specific bands were processed by LC/MS by OE Biotechnology (China). Candidate proteins are listed in Additional file 2: Table S3. All kit and reagent information is listed in Additional file 2: Table S9.

RIP assays were performed with the Magna Nuclear RIP (Native) Nuclear RNA-Binding Protein Immunoprecipitation Kit (Merck Millipore) according to the manufacturer’s instructions. After antibody incubation and proteinase K digestion, the immunoprecipitated RNAs were extracted, purified, and subjected to qPCR. RNA levels were normalized to the input (10%). All antibody and kit information is listed in Additional file 2: Table S9.

#### Chromatin immunoprecipitation (ChIP) assay

ChIP assays were performed with the ChIP-IT Express Kit (ActiveMotif, USA). In brief, the cells (1 × 10^7^) were lysed in lysis buffer and sonicated to obtain chromatin fragments (100–500 bp) using the Covaris M220 Focused-Ultrasonicator (USA). The chromatin fragments and magnetic beads were incubated with antibodies at 4 °C overnight. After washing, the immunoprecipitated DNA was eluted, purified, and detected by qRT-PCR using the Applied Biosystems Prism 7500 Fast Sequence Detection System. All primer information is listed in Additional file 2: Table S8, and all kit information is provided in Additional file 2: Table S9.

### Subcellular fractionation

The PARIS protein and RNA Isolation Kit (Invitrogen) was used to detect *LINC01977* expression in cytoplasmic and nuclear fractions according to the manufacturer’s instructions. RNA was extracted from cytoplasmic and nuclear fractions and subjected to qPCR. GAPDH was used as the cytoplasmic marker, while U6 was used as the nuclear marker. All primer information is listed in Additional file 2: Table S8, and all kit information is provided in Additional file 2: Table S9.

### Tumor xenograft and lung metastasis models

BALB/c nu/nu female mice at 4 to 6 weeks old were purchased from Gem Pharmatech (China) and maintained according to protocols approved by the Institutional Animal Care and Use Committee of Nanjing Medical University.

Single-cell suspensions of 2 × 10^6^ A549 cells in 50 μL of diluted Matrigel (1:1) were injected subcutaneously into the dorsal flanks of 4-week-old mice. The mice were randomized into two groups (*n* = 5/group) until their tumors reached a size of approximately 100 mm^3^. Next, the mice were treated with intratumoral injections of scrambled or in vivo-optimized *LINC01977* inhibitor (10 nmol per injection, RiboBio) every 3 days. Tumor growth was monitored every 3 days using calipers. After 4 weeks, the mice were killed, and the tumor volume and weight were measured.

For the lung metastasis model, single-cell suspensions of 1 × 10^6^ A549 cells in 100 μL of sterilized PBS were injected into the tail vein of 4-week-old mice. The mice were randomized into four groups until the metastatic foci in lungs were detected by the IVIS Spectrum In Vivo Imaging System (IVIS Lumina XR, USA). Next, the mice were injected into the tail vein with vehicle [formulated in ethanol/cremophor/water at 10:10:80 (v/v/v)], *LINC01977*-ASO (10 nmol), SGC-CBP30 (25 mg/kg), or combination. The drug was administered every 3 days, and the mice were monitored for survival. After 4 weeks, the mice were evaluated by the IVIS. All mice were monitored until death. All tissues from the cell-based xenograft or lung metastasis model underwent further pathological analysis. All reagent information is listed in Additional file 2: Table S9.

### In silico bioinformatics analysis

RNA expression profiles and survival information from patients in The Cancer Genome Atlas-LUAD (TCGA-LUAD) were downloaded from UCSC Xena (https://xenabrowser.net/), while those without integrated survival information were removed from further evaluation. The analysis of differentially expressed genes (DEGs) was performed using the “*DESeq2*” R package. Gene Set Enrichment Analysis (GSEA) was performed using the “clusterProfiler” R package in “hallmark gene sets” (msigdb.v7.0.entrez.gmt). The tumor immune microenvironment was assessed by CIBERSORT. The data of H3K27ac and H3K4me1 ChIP-seq from A549 cells (human alveolar adenocarcinoma cell line, ENCODE Accession: ENCFF651IDK, ENCFF055ATH), PC-9 cells (human lung adenocarcinoma cell line, ENCODE Accession: ENCFF500YWU, ENCFF485RPN), normal lung tissue (ENCODE Accession: ENCFF868JVP, ENCFF902ZAM), and the data of Hi-C from A549 cells (ENCODE Accession: ENCFF121YPY) were downloaded from Encyclopedia of DNA Elements (ENCODE). Peak calling was performed using MACS2 (parameters: -keep-dup all -g hs -q 0.01). The calling of SE was carried out with ROSE. All parameters were set to their default values. SEs identified by ROSE with a length < 2 kb were filtered out. The GREAT (version 3.0.0) online tool was used to identify genes linked to SEs based on the association rule: basal + extension: 1 kb upstream, 1 kb downstream, and 1000 kb max extension. These data were visualized by IGV 2.9.4 and Juicebox 1.11.08 online tools.

### Statistical analysis

Statistical analysis was performed using GraphPad Software 9 (USA). The survival curves for prognostic analysis were generated using the Kaplan–Meier method, and log-rank tests were utilized to identify the significance of the differences. Correlations were analyzed by the Pearson correlation test. All data are expressed as the means ± standard deviation (SD). The Student’s t test was used for two-group comparisons. For comparisons among more than two groups, the Wilcoxon test and one-way ANOVA were used for non-parametric and parametric data. *P* > 0.05 was considered not significant (*n*.s.), and *P* < 0.05 was considered statistically significant (*P < 0.05; **P < 0.01; ***P < 0.001).

## Results

### Identification of *LINC01977* as a super-enhancer-associated cancer-testis lncRNA

**Fig. 1:**
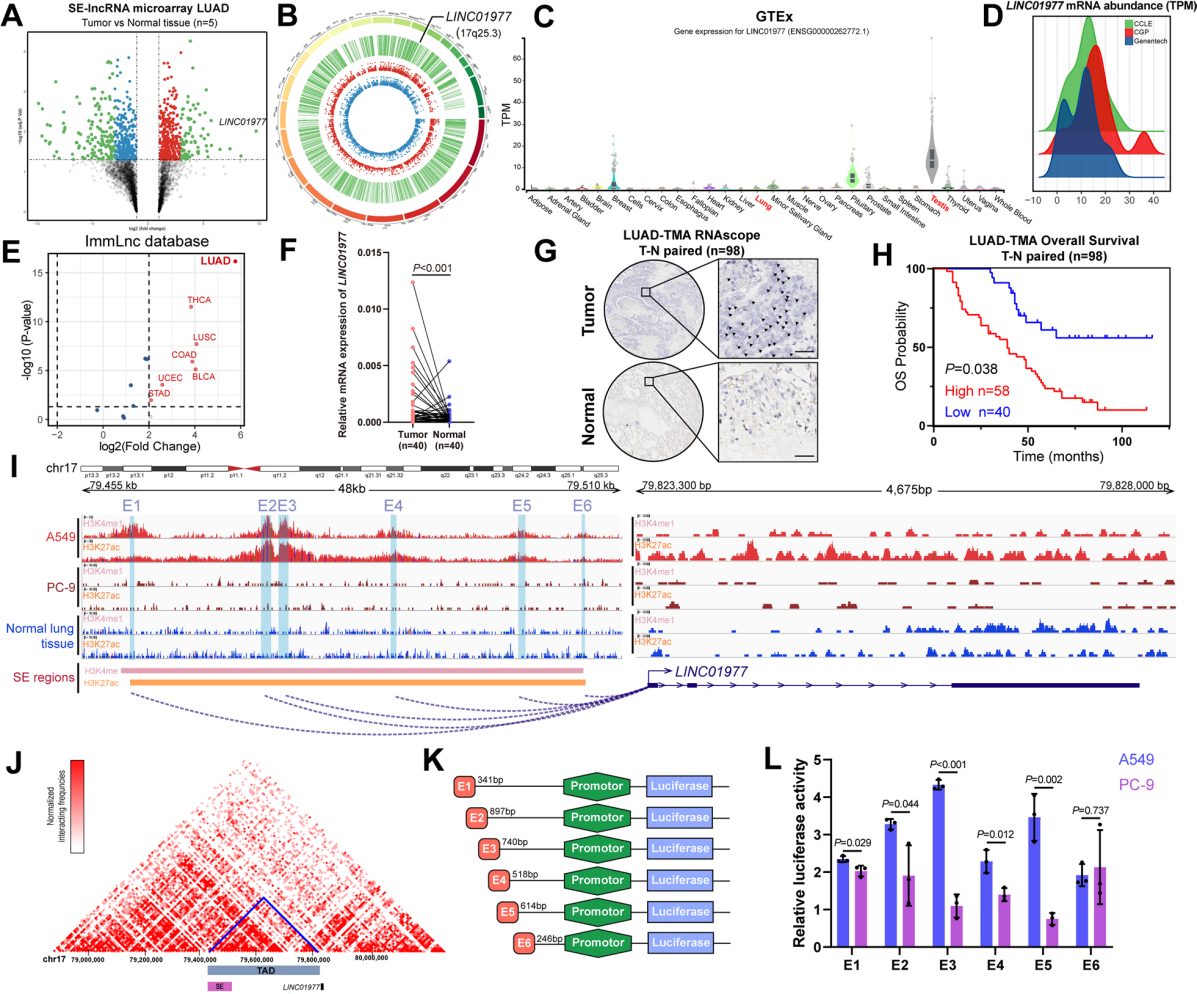
Identification of *LINC01977* as a super-enhancer-associated cancer-testis lncRNA. **A** Volcano plot of gene expression microarray results. The *x*-axis shows fold change between normal and LUAD samples and the *y*-axis shows one-way ANOVA *P* value; points corresponding to twofold change cutoff and *P* < 0.05 are colored red (increasing) and blue (decreasing), points corresponding to significantly decreasing or increasing lncRNAs are colored green, respectively. **B** The circos plot showing dysregulated lncRNAs found with SE-associated lncRNAs microarray analysis of 5 tumor-normal pairs LUAD samples. **C** Tissue-specific gene expression from GTEx data for *LINC01977*. **D** Density plots are shown expression of *LINC01977* in cancer cells from CCLE, CGP and Genentech data. **E** Volcano plot of *LINC01977* expression from ImmLnc database (http://bio-bigdata.hrbmu.edu.cn/ImmLnc/). The *x*-axis shows fold change between normal and tumor samples, and the y-axis shows one-way ANOVA *P* value; points corresponding to twofold change cutoff and *P* < 0.05 are colored red (significantly increasing). **F** qPCR detection of *LINC01977* expression in LUAD (*n* = 40) tumor-normal paired tissues. **G** RNAscope ISH detection of *LINC01977* expression in LUAD (*n* = 98) tumor-normal paired tissue microarray. Arrows denote *LINC01977* RNAscope ISH red dots. Scale bar: 20um. **H** Kaplan–Meier survival curves of LUAD patients on tissue microarray grouped by *LINC01977* expression. **I** Gene tracks depicting the gene body and super-enhancer region of *LINC01977* in LUAD cells A549 and PC-9, as well as normal lung tissue with measured H3K27ac and H3K4me1 marks. The data were retrieved from Encode project. (**J**) *LINC01977* in A549 cell line topologically associated domain (TAD) region was predicted on the basis of the Hi-C data (http://promoter.bx.psu.edu/hi-c/view.php). **K** Schematic representation of constructing enhancer-promoter dual-luciferase reporter plasmids strategy. (**L**) The luciferase activities of six enhancer elements were measured through dual-luciferase reporter assay in A549 and PC-9 cells. Data in L are representative of three independent experiments and presented as mean ± S.D., *n* = 3 biologically independent samples, and the *P* value was determined by a two-tailed unpaired Student’s *t* test and one-way ANOVA, respectively. *P ≤ 0.05; **P ≤ 0.01; ***P ≤ 0.001; *n*.s. not significantly

**Fig. 2:**
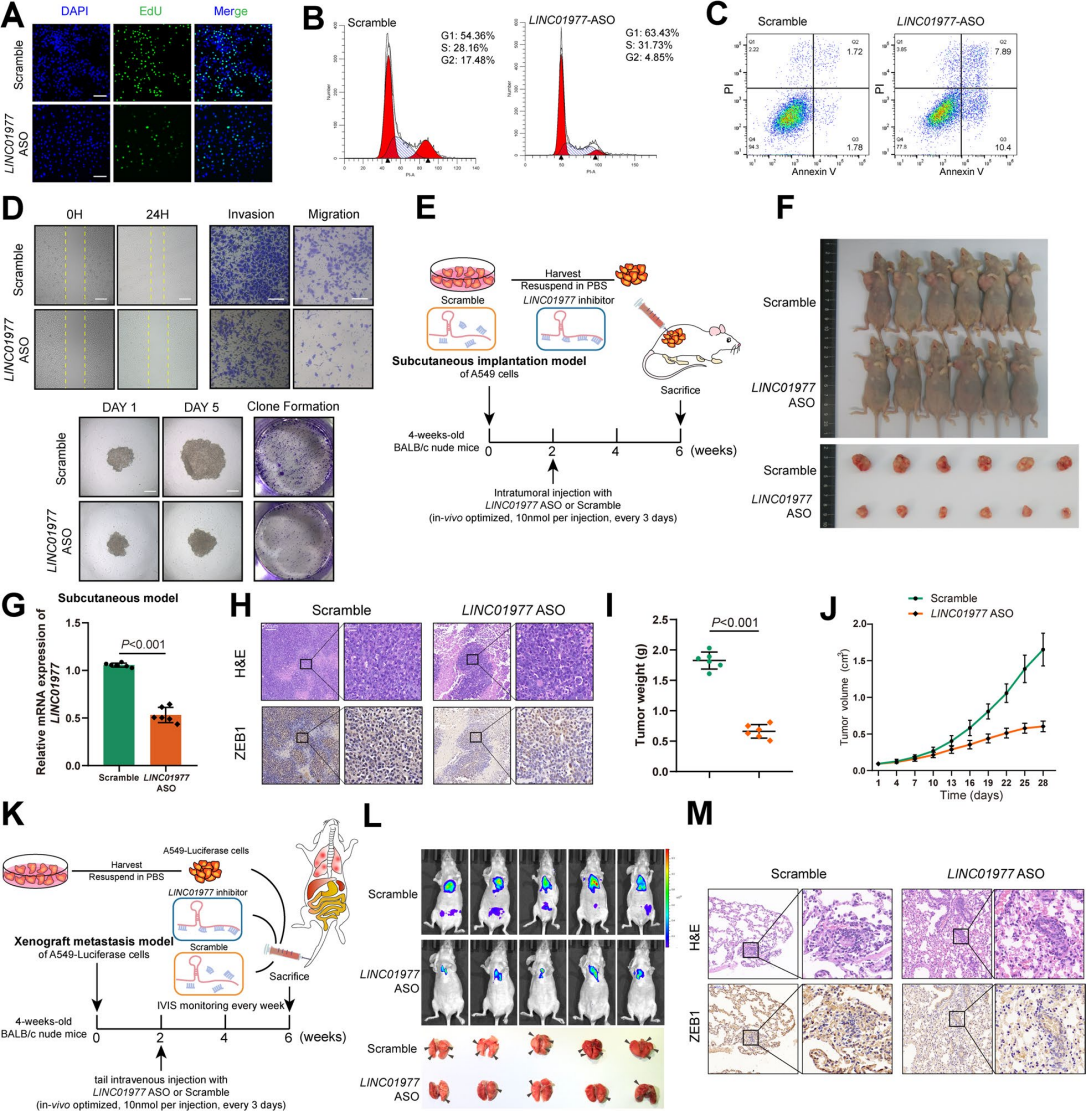
*LINC01977* exerts oncogenic roles in vitro and in vivo. **A** Number of proliferating *LINC01977*-silenced A549 cells, as determined by the EdU incorporation assay. Cells transfected with scramble were used as control. DAPI was used to stain the nuclei. Scale bars: 200 μm. **B** Different cell cycle phases of *LINC01977*-silenced A549 cells. Cells were stained using propidium iodide (PI), and the percentages were determined by flow cytometry. **C** The apoptosis of *LINC01977*-silenced A549 cells. Cells were stained using PI and annexin V, and the percentages were determined by flow cytometry. **D** The invasion, migration of *LINC01977*-silenced A549 cells assessed by wound healing and transwell assays (up panel). The proliferation ability of *LINC01977*-silenced A549 cells assessed by spheroid formation and colony formation assay (down panel). **E** Schematic representation of xenograft nude mouse model. **F** Representative images of mice-bearing tumors derived from xenograft nude mouse model. **G** qRT-PCR showing mRNA expression levels of LINC01977 in tumor tissues obtained from subcutaneous model. **H** H&E stained and ZEB1 IHC of serial sections from mice-bearing tumors. H&E, scale bars: 100 μm; IHC, scale bars: 20 μm. Tumor weight (**I**) and tumor volume (**J**) were assessed. Data point in represent (mean ± SEM) are shown from *n* = 6 biologically independent samples by two-sided unpaired t test. **K** Schematic representation of xenograft metastasis model. **L** Representative images of IVIS detection and metastatic loci in the lung derived from mouse model. **M** H&E stained and ZEB1 IHC detection of serial sections from lung metastasis nude mouse model. H&E, scale bars: 100 μm; IHC, scale bars: 20 μm. Data in A–D are representative of three independent experiments and presented as mean ± S.D., A–D (*n* = 3), G, I and J (*n* = 6) biologically independent samples, and the *P* value was determined by a two-tailed unpaired Student’s *t* test and one-way ANOVA, respectively

To characterize the dysregulated lncRNAs regulated by SEs, super-enhancer-associated lncRNA (SE-lncRNA) microarrays were used for five paired tumor and adjacent normal tissues from LUAD patients (Additional file 2: Table S1), which revealed that 515 lncRNAs were differentially expressed, including 189 significantly up-regulated and 326 significantly down-regulated lncRNAs in malignant tissues compared with normal tissues. By overlapping the dysregulated lncRNA from TCGA and our microarray analysis data, we obtained 55 dysregulated lncRNAs (Additional file 1: Figure S1A, B), of which 18 lncRNA transcripts have been validated in NCBI (https://www.ncbi.nlm.nih.gov/). We focused our attention on the transcript *LINC01977* due to the following reasons: (1) It was the most significantly differentially expressed lncRNA in the analysis (Fig. 1A); (2) it was localized in 17q25.3 (Fig. 1B), whose genomic instability has been reported to be associated with the malignant progression of NSCLC [20]; and (3) analysis of the GTEx dataset demonstrated that *LINC01977* showed high expression in the normal testis (Fig. 1C), referring to cancer-testis genes, which have potential roles in tumorigenesis and progression [21].

Additionally, pan-cancer analysis from the public database showed that *LINC01977* showed higher expression in cancer cell lines (CCLE, CGP, and Genentech) (Fig. 1D) and LUAD tumor tissues (ImmLnc dataset) (Fig. 1E). Subsequently, we performed qRT-PCR on 40 paired LUAD tissues and observed that *LINC01977* was highly expressed in paired LUAD tumor tissues (Fig. 1F), which was validated in the other 186 unmatched LUAD tumor tissues (Additional file 1: Figure S1C) and the GEO dataset of LUAD (Additional file 1: Figure S1D, E). Next, we performed RNAscope in situ hybridization (ISH) to detect *LINC01977* expression in the tissue microarray -containing 98 pairs of well-annotated LUAD patients (Fig. 1G, Additional file 2: Table S2). Our results demonstrated that *LINC01977* was highly expressed in LUAD tissues and correlated with poorer overall survival (Fig. 1H).

As *LINC01977* was annotated as a SE-associated lncRNA in the aforementioned microarray, we assessed the impact of the SE on the transcriptional regulation of *LINC01977*. We mined the H3K27ac and H3K4me1 ChIP-seq data, the active markers of the SE, in the canonical LUAD cell lines (A549 and PC-9) and normal lung tissue from the Encyclopedia of DNA Elements (ENCODE) database, and an aberrantly activated SE region located upstream of *LINC01977* spanning 48 kb was identified in A549 cells (Fig. 1I). However, in the PC-9 cell line, there were only modest changes in these specific peaks, which may have been due to the tissue and cell line specificity of SE [22]. Moreover, Hi-C data of A549 cells from the public database highlighted that the SE region directly interacted with the promoter region of *LINC01977* (Fig. 1J). To clarify whether the SE region we identified in A549 cells could affect the transcription of *LINC01977*, the SE region of *LINC01977* was divided into six constituents (E1–E6), which were designed as dual-luciferase reporter gene plasmids, containing the *LINC01977* promoter region (Fig. 1K). Consistent with SE-specific peaks we obtained from ChIP-seq data, majority of SE sub-region (5/6) were generally higher in A549 cells compared to PC-9 cells. The results suggested that SE caused strong transcription-enhancing activity in *LINC01977* in A549 cells, while moderate transcription-enhancing activity in PC-9 cells (Fig. 1L). Moreover, treatment with the BRD4 inhibitor JQ1 decreased the *LINC01977* mRNA level in A549 cells, while mRNA alteration of the neighboring gene *CBX4* was not significant, consistent with the feature of SE-lncRNAs (Additional file 1: Figure S1F). Additionally, the *LINC01977* mRNA level was different in A549 cells compared with the other LUAD cell lines and normal lung cell line (Additional file 1: Figure S1G). To exclude the possibility that this difference is due to the driver mutation status in LUAD cell lines, we performed correlation analysis in TCGA-LUAD dataset, which suggesting that no differences were detected in *LINC01977* expression by mutation status (Additional file 1: Figure S1H-L). Collectively, these findings indicated that the cancer-testis lncRNA, *LINC01977*, was driven by the aberrantly activated SE in LUAD.

### *LINC01977* exerts oncogenic roles in vitro and in vivo

To investigate the function of *LINC01977* in the malignant progression of LUAD, we performed loss-of-function experiments in vitro and in vivo. Antisense oligonucleotides (ASO) were selected to inhibit *LINC01977* expression. The transfection efficiency in LUAD cell lines were evaluated by qRT-PCR (Additional file 1: Figure S2A, B). We performed EdU assays and demonstrated that *LINC01977* promoted proliferation of the LUAD cell line (Fig. 2A, Additional file 1: Figure S2C). Additionally, cell cycle assessment suggested that the absence of *LINC01977* blocked the G1/S cell cycle transition (Fig. 2B, Additional file 1: Figure S2D). Next, annexin V staining followed by flow cytometry analysis showed increased apoptosis in *LINC01977*-deficient LUAD cells compared with the control LUAD cells (Fig. 2C, Additional file 1: Figure S2E). The migration and invasion abilities of LUAD cells were detected by wound healing, Transwell, and Matrigel invasion assays, which suggested that the absence of *LINC01977* significantly reduced cell migration and invasion (Fig. 2D, Additional file 1: Figure S2F–H). Furthermore, 3-D tumor sphere formation and 2-D plate clone formation were assessed to evaluate the clonogenicity of LUAD cells, and the results revealed that the absence of *LINC01977* reduced the clonogenicity of LUAD cells (Fig. 2D, Additional file 1: Figure S2I, J). These results revealed that *LINC01977* deficiency attenuated LUAD cells proliferation and malignant phenotypes in vitro.

Next, we investigated the biological functions of *LINC01977 *in vivo. Nude mice were injected with A549 cells to establish a subcutaneous tumor bearing model, and *LINC01977*-ASO or the control was injected intratumorally every 3 days (Fig. 2E). The results indicated that tumors injected with *LINC01977*-ASO were smaller in size, lighter in weight, and retarded in growth compared with controls; meanwhile, efficient *LINC01977* knockdown was confirmed by qRT-PCR (Fig. 2F–J). Additionally, we assessed whether inhibition of *LINC01977* affected tumor metastasis in the xenograft metastasis model (Fig. 2K). Cancer cell colonization in secondary organs was monitored by the IVIS (Fig. 2L). Analysis of lung metastatic colonies indicated that *LINC01977*-ASO suppressed the formation of distant metastases (Fig. 2L, M, Additional file 1: Figure S2K). Taken collectively, these findings indicated that *LINC01977* behaved as an oncogene to promote the malignant progression of LUAD.

### *LINC01977* binding to SMAD3 protein is dependent on the MH2 domain

**Fig. 3:**
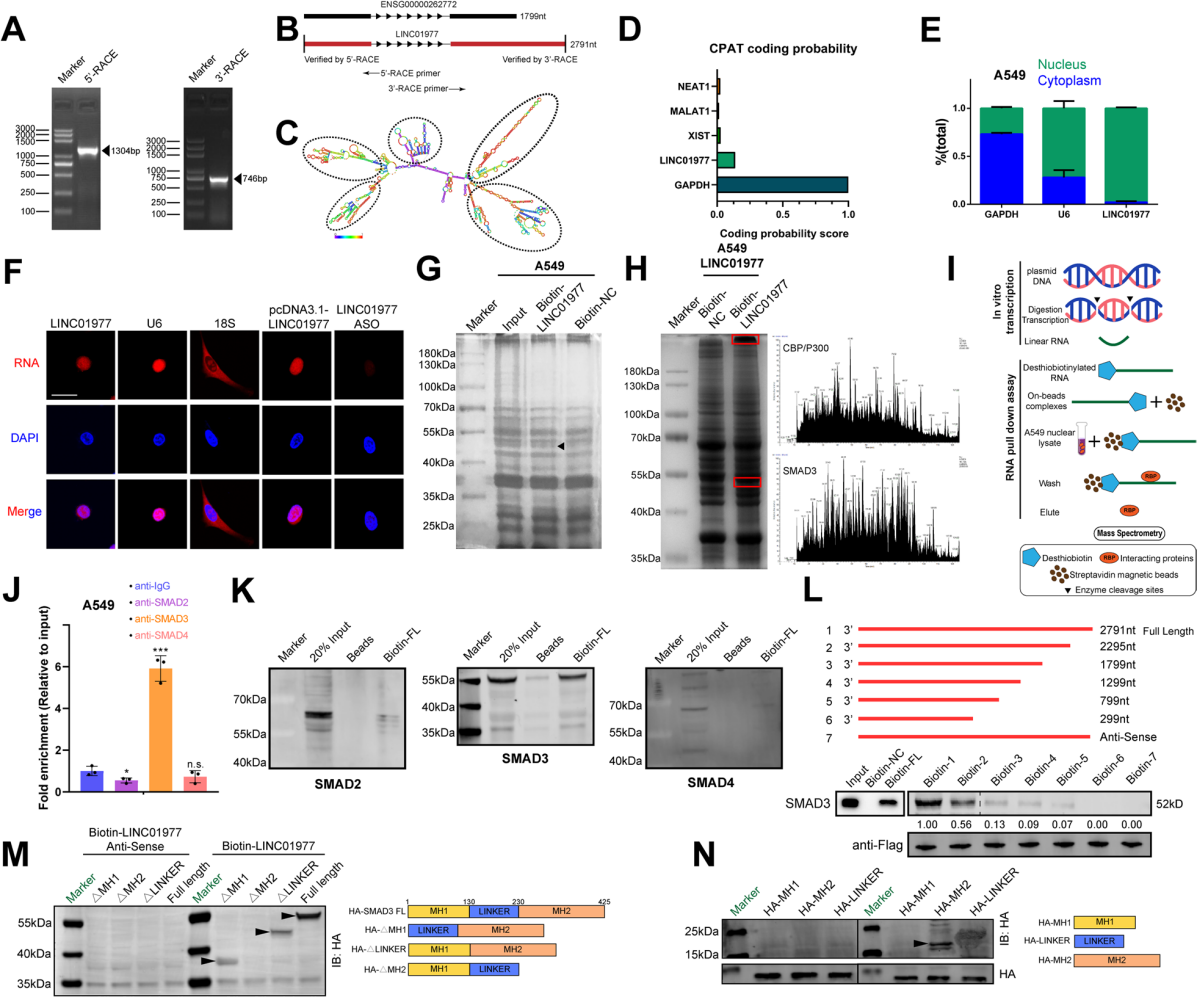
*LINC01977* binding to SMAD3 protein is dependent on the MH2 domain. **A** PCR products from the 3′-RACE and 5′-RACE procedures as shown by agarose gel electrophoresis. **B** Full length of *LINC01977* predicted in the UCSC Genome Browser and measured *LINC01977* acquired by 3′-RACE and 5′-RACE. **C** *LINC01977* is predicted to have five stem-loop structures, which were indicated with dashed circles. **D** Coding potential was determined by Coding Potential Assessment Tool (CPAT). **E** qRT-PCR detection of *LINC01977* expression in the cytoplasmic and nuclear fractions. **F** Subcellular localization of *LINC01977* detected by FISH. Scale bar: 5 μm. **G** Protein retrieved from the *LINC01977* pull-down assay in A549 cells were analyzed by SDS-PAGE. Specific band is indicated with arrow. **H** Protein retrieved from the *LINC01977* pull-down assay in *LINC01977* overexpressing A549 cells were analyzed by SDS-PAGE (left panel). Specific bands are indicated with red boxes, and results from LC/MS analysis were shown (right panel). **I** Schematic representation of RNA pull-down assay. **J** RIP assays were performed using anti-SMAD2/3/4 antibody in A549 cells. qRT-PCR was used to measure the enrichment of *LINC01977*. **K** RNA pull-down assays were performed using in vitro transcribed biotinylated full-length *LINC01977* and magnetic beads (as negative control). Western blot was used to evaluate the interaction between *LINC01977* and SMAD2/3/4. **L** Immunoblot detection of the SMAD3 protein in A549 cells as retrieved by in vitro transcribed biotinylated RNAs of different constructs of *LINC01977* or its antisense sequence (as negative control). **M**, (**N**) RNA pull-down assays were performed using in vitro transcribed biotinylated full-length *LINC01977* and its antisense sequence (as negative control), as well as different HA-tagged SMAD3 protein truncations. Western blot was used to evaluate the interaction between *LINC01977* and SMAD3 protein truncations by anti-HA antibody. Data in E and J are representative of three independent experiments and presented as mean ± S.D., *n* = 3 biologically independent samples, and the *P* value was determined by a two-tailed unpaired Student’s *t* test and one-way ANOVA, respectively. *P ≤ 0.05; **P ≤ 0.01; ***P ≤ 0.001; *n*.s. not significantly

To determine the exact full length of *LINC01977*, we performed 5’ and 3’ rapid amplification of complementary DNA ends (RACE) assays. The results revealed that *LINC01977* was a 2791-nt lncRNA, which was longer than the sequence we obtained from NCBI and contained stem-loop structure (Fig. 3A-C, Additional file 1: Figure S3). According to the coding potential assessment tool (CPAT), the score of *LINC01977* was very low compared with *GAPDH*, a well-known coding RNA, which was consistent with the characteristics of lncRNAs (Fig. 3D). To explore the subcellular localization of *LINC01977*, we detected its expression in cytoplasmic and nuclear fractions by qRT-PCR analysis, which indicated that *LINC01977* was predominantly localized in the nucleus (Fig. 3E). This was further verified by RNA fluorescence in situ hybridization (FISH) assays (Fig. 3F).

It has been widely reported that lncRNAs exert their biological effects by binding to proteins [23]. To uncover the mechanism underlying the role of *LINC01977* in LUAD, we performed RNA pull-down assays and subjected the precipitates to mass spectrometry analysis to identify the potential *LINC01977*-interacting proteins. The silver staining results showed enrichment of several bands of proteins potentially combined with *LINC01977*, distributed in the ~ 40–55 kDa and > 180 kDa regions (Fig. 3G–I). According to the unique peptides and cover percentages from the assays, we focused on the SMAD3 protein for the next experiment (Additional file 2: Table S3). CatRAPID was used to predict the potential SMAD3-binding regions in *LINC01977* and the potential protein-binding domains of *LINC01977* in SMAD3 (Additional file 1: Figure S4A). RIP and RNA pull-down assays confirmed that *LINC01977* interacted with SMAD3, and not with SMAD2 or SMAD4 (Fig. 3J–K, Additional file 1: Figure S4B, C), the important component of the R-SMAD complex.

To clarify the nucleotide sequence of *LINC01977* that binds to SMAD3, we constructed a series of *LINC01977* deletion mutants according to the stem-loop structures (Fig. 3C, Additional file 1: Figure S4D). The results showed that deletion of the RNA fragment ranging from 1799 to 2791 significantly decreased the binding to SMAD3, which revealed that this region was critical for the interaction of *LINC01977* with SMAD3 (Fig. 3L). Similarly, to assess the precise functional domains of SMAD3 interacting with *LINC01977*, we generated N-terminal HA fusion-tagged full-length (FL) or truncated SMAD3 constructs containing the MH1 domain (amino acids 1–130), linker domain (amino acids 130–230), and MH2 domain (amino acids 230–425) (Fig. 3M). RNA pull-down assays revealed that *LINC01977* bound to the MH2 domain of SMAD3, which is critical for the translocation, phosphorylation, and transcription factor/co-activator binding of SMAD3 (Fig. 3N) [24]. We also demonstrated that high expression of SMAD3 was associated with poor prognosis in patients with LUAD in both TCGA-LUAD and LUAD GEO datasets (GSE31210 and GSE13213) (Additional file 1: Figure S4E). Taken collectively, these results revealed that *LINC01977* bound with the MH2 domain of SMAD3 protein.

### LUAD cells adapt to TAM2-induced TGF-β enrichment in the tumor microenvironment via SE-*LINC01977*-dependent TGF-β/SMAD3 signaling pathway activation

**Fig. 4:**
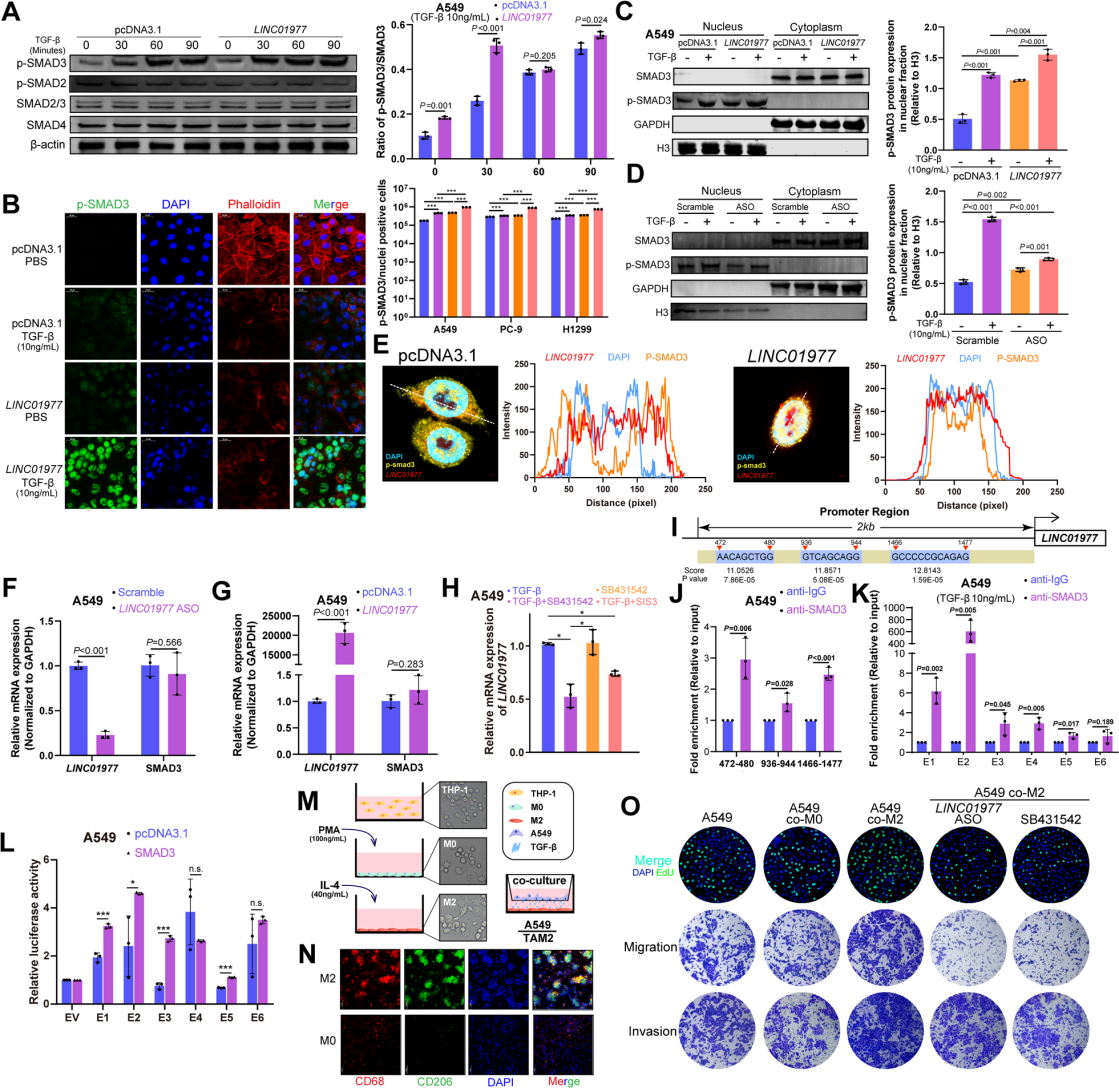
LUAD cells adapt to TAM2-induced TGF-β enrichment in the tumor microenvironment via SE-*LINC01977*-dependent TGF-β/SMAD3 signaling pathway activation. **A** Western blotting detection of phosphorylated SMAD3 (Ser423/425) levels in A549 cells transfected with pcDNA3.1 or *LINC01977* followed by treatment with TGF-β (10 ng/mL) for the indicated times (left panel). Quantitation of protein levels (right panel). **B** Represent images of phosphorylated SMAD3 (Ser423/425) nuclear translocation, assayed by immunofluorescence confocal microscopy in A549 cells with *LINC01977* overexpressed followed by treatment with TGF-β (10 ng/mL) (left panel). Actin, Phalloidin (red); nuclei, DAPI (blue); and phosphorylated SMAD3 Ser423/425 (green). Scale bar: 20 μm. Quantification of nuclear fluorescence of p-SMAD3 relative to total nucleus fluorescence. **C** Western blotting detection of phosphorylated SMAD3 (Ser423/425) levels in nucleoplasm and cytoplasm fractions of A549 cells transfected with pcDNA3.1 or *LINC01977* followed by treatment with TGF-β (10 ng/mL) (left panel) Quantitation of protein levels (right panel). **D** Western blotting detection of phosphorylated SMAD3 (Ser423/425) levels in nucleoplasm and cytoplasm fractions of A549 cells transfected with Scramble or *LINC01977*-ASO followed by treatment with TGF-β (10 ng/mL) (left panel). Quantitation of protein levels (right panel). **E** Confocal images showing the co-localization of *LINC01977* and phosphorylated SMAD3 (Ser423/425) in A549 cells transfected with pcDNA3.1 or *LINC01977* followed by treatment with TGF-β (10 ng/mL) for 2 h. Scale bar: 2 μm. Fluorescence intensity was quantified using ImageJ. **F** qRT-PCR detection of *LINC01977* and SMAD3 expression in A549 cells treated with scramble or *LINC01977-*ASO. **G** qRT-PCR detection of *LINC01977* and SMAD3 expression in A549 cells transfected with pcDNA3.1 or *LINC01977*. **H** qRT-PCR detection of *LINC01977* expression in A549 cells treated with TGF-β (10 ng/mL), TGF-β inhibition SB431542 (10 μM) and SIS3 (5 μM) as indicated. **I** The *LINC01977* promoter contains SMAD3-binding sites. **J** ChIP assays suggested that SMAD3 bound to the *LINC01977* promoter. (**K**) ChIP assays suggested that SMAD3 bound to the *LINC01977* SE. **L** SMAD3 overexpression elevated the SE-induced transcriptional activity of *LINC01977* in A549 cells. **M** Schematic representation of THP-1-derived M2-like macrophages and co-culture model. **N** M2 marker expression, CD68 and CD206, were detected by IF staining. Scale bar: 50 μm. (**O**) Cell proliferation was assessed by EdU incorporation assay; EdU (green); and nuclei, DAPI (blue), respectively (upper panel). Abilities of invasion and migration were detected by transwell assays, respectively (middle and lower panel). Scale bar: 50 μm. Data in A, C, E–K, O are representative of three independent experiments and presented as mean ± S.D., *n* = 3 biologically independent samples, and the *P* value was determined by a two-tailed unpaired Student’s *t* test and one-way ANOVA, respectively. *P ≤ 0.05; **P ≤ 0.01; ***P ≤ 0.001; *n*.s. not significantly

TGF-β signaling is mainly mediated through the canonical SMAD3 signaling pathway. TGF-β-mediated phosphorylation of SMAD3 on Ser423/425 is essential for TGF-β/SMAD3 signal transduction [25]. To assess whether *LINC01977* was involved in the TGF-β/SMAD3 signaling pathway, we detected phosphorylated SMAD3 Ser423/425 in a time-dependent TGF-β treated manner. We found that phosphorylated SMAD3 protein expression was elevated within 30 min of TGF-β treatment in *LINC01977*-overexpressing LUAD cells, suggesting that *LINC01977* contributes to the SMAD3-mediated early TGF-β response (Fig. 4A, Additional file 1: Figure S5A, B). To clarify the impact of *LINC01977* on p-SMAD3, immunofluorescence assays were performed in A549 cells. The results indicated that p-SMAD3 mainly appeared in the nucleus in TGF-β treated A549 cells and predominantly accumulated in the nucleus following *LINC01977* overexpression (Fig. 4B). Next, LUAD cells were harvested for nuclear and cytoplasmic fractions at 24 h after gain- or loss- of *LINC01977*. Fractionation followed by western blotting analysis demonstrated that *LINC01977* promoted p-SMAD3 nuclear accumulation, which was consistent with the immunofluorescence results (Fig. 4C, Additional file 1: Figure S5C, D). Furthermore, *LINC01977* deficiency attenuated p-SMAD3 nuclear translocation as well under TGF-β stimulation in LUAD cells (Fig. 4D, Additional file 1: Figure S5E, F).

To analyze the intracellular distribution of p-SMAD3 in A549 cells, we used two-photon confocal microscopy. Unsurprisingly, *LINC01977* overexpression significantly promoted p-SMAD3 nuclear accumulation after TGF-β stimulation (Fig. 4E). Moreover, perturbations in the *SMAD3* mRNA level were not observed in the presence or absence of *LINC01977* in A549 cells (Fig. 4F, G). Next, we assessed the impact of the TGF-β/SMAD3 signaling pathway on the expression of *LINC01977*. We used the p-SMAD3 inhibitor SIS3 and the TGF-β activation inhibitor SB431542, and the results revealed that *LINC01977* expression was significantly decreased during TGF-β inhibition and p-SMAD3 inactivation in LUAD cells (Fig. 4H, Additional file 1: Figure S5G). These results indicated that *LINC01977* expression was associated with the activation of the TGF-β/SMAD3 signaling pathway.

A previous study has reported that SMAD3 is a key transcription factor in the malignant progression of tumors. In LUAD, survival analysis revealed that high-expression SMAD3 was associated with poor prognosis (Additional file 1: Figure S4E) [26]. Motif analysis showed three predicted SMAD3-binding sites in the *LINC01977* promoter region (Fig. 4I). The results of ChIP-qPCR revealed SMAD3 occupancy in the *LINC01977* promoter (Fig. 4J). Furthermore, luciferase reporter assay revealed that *LINC01977* expression was promoted in a TGF-β/SMAD3 partially dependent manner (Additional file 1: Figure S5H). Interestingly, SMAD3 occupancy was also observed in the SE region of *LINC01977* in LUAD cells, which demonstrated that SMAD3 transcriptionally regulates *LINC01977* via promoter binding and epigenetic regulation of SE occupancy (Fig. 4K, Additional file 1: Figure S5I). Indeed, luciferase reporter assays supported this conclusion (Fig. 4L).

Tumor-associated macrophages (TAMs), highly abundant components in the tumor microenvironment, construct pre-metastasis niches [27]. In the malignant progression of LUAD, the abundance of TGF-β in the tumor microenvironment was attributed to the high infiltration of M2-like TAMs (TAM2) [28]. By GSEA, we also found that high M2 infiltration in LUAD was significantly associated with TGF-β signaling pathway activation and enriched in early-stage LUAD (Additional file 1: Figure S5J, K). Accordingly, we used the human monocyte THP-1-derived in vitro model that mimics this phenotype of TAM2 for co-culture with LUAD A549 cells (Fig. 4M), and the results were validated by the TAM2 specific marker CD68/CD206 via both IF and Flow cytometry analysis (Fig. 4N, Additional file 1: Figure 5L). Next, the secretion of TGF-β was confirmed by enzyme linked immunosorbent assay (ELISA) (Additional file 1: Figure S5M). qRT-PCR and functional experiments provided evidence that co-culturing with TAM2 increased *LINC01977* expression in LUAD cells and promoted the EMT process, consistent with TGF-β-induced malignant progression. These observations were also validated by *LINC01977* and TGF-β dependent experiments (Fig. 4O, Additional file 1: Figure S5N-P). Taken collectively, these results confirmed that TAM2 promoted the LUAD malignant phenotype.

It has been reported that lncRNAs are often enriched in a tissue- or cell-specific manner [29]. Studies on epigenetic diversity and heterogeneity in cancer have demonstrated that SEs are also highly cell-specific [30, 31]. To investigate how LUAD cells adapt to complex tumor microenvironments, we first detected *LINC01977* in LUAD cells and tumor microenvironment-associated cells. To this end, we obtained tumor microenvironment-associated cells derived from peripheral blood mononuclear cells (PBMCs), including CD4^+^ T cells, CD3^+^ T cells, CD8^+^ T cells, and DCs, as well as cancer-associated fibroblasts (CAFs). We observed that *LINC01977* was highly enriched in LUAD cell lines. Additionally, upon stimulation with TGF-β, increased *LINC01977* expression was observed in LUAD cells (A427, H1299, SW1573, and H1975), but the changes were not significant in both tumor microenvironment-associated cells and human normal bronchial epithelium (HBE) cells (Additional file 1: Figure S5Q, R). These observations potentially reflected that high *LINC01977* expression was the specific response of LUAD cells to TAM2 infiltration induced by TGF-β enrichment in the tumor microenvironment. As previously shown, *LINC01977* was involved in the TGF-β/SMAD3 signaling pathway, and these results were likely due to LUAD cell adaption to TAM2 infiltration. Additionally, this adaptive behavior was validated by functional experiments (Fig. 4O, Additional file 1: Figure S5O). Thus, our finding uncovered that LUAD cells adapt to TAM2-induced TGF-β enrichment in the tumor microenvironment in two ways: (i) by sensing and activating the canonical TGF-β signaling pathway and (ii) by SE-*LINC01977* binding and tethering SMAD3 in the nucleus to promote TGF-β/SMAD3 signaling transduction via epigenetic regulation.

### Epigenetic transcriptional activation of the SMAD3/CBP/P300 complex is *LINC01977*-dependent and can potentially serve as a target for combination therapy

**Fig. 5:**
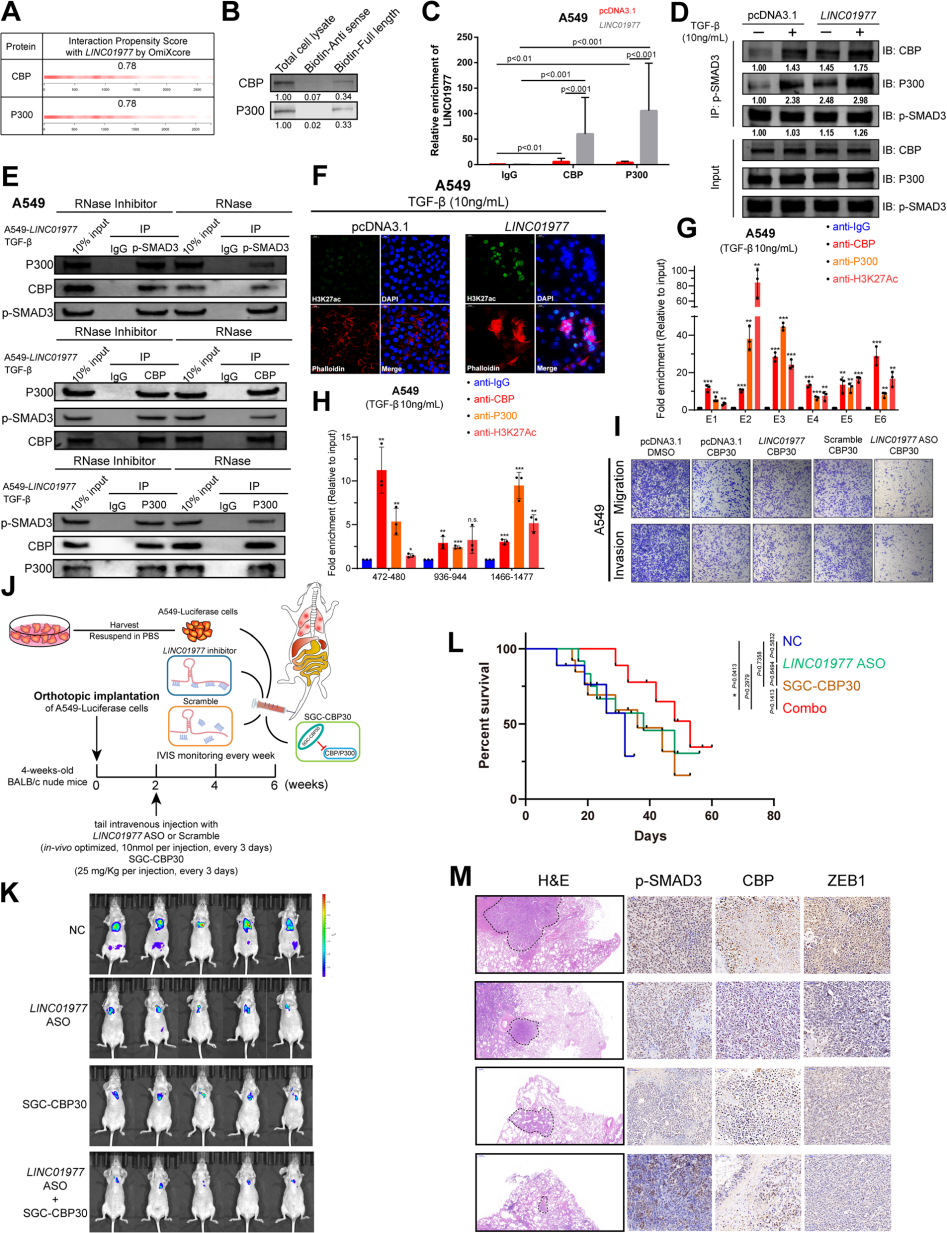
Epigenetic transcriptional activation of the SMAD3/CBP/P300 complex is *LINC01977*-dependent and can potentially serve as a target for combination therapy. **A** RNA-binding abilities of *LINC01977* and CBP/P300 were predicted by OmiXcore. **B** RNA pull-down and western blot assays were used to evaluate the interaction between *LINC01977* and CBP/P300. **C** RIP and qRT-PCR assays were used to measure the enrichment of *LINC01977*. **D** Co-immunoprecipitation (co-IP) of CBP or P300 with phosphorylated SMAD3 Ser423/425 in A549 cells transfected with pcDNA3.1 or *LINC01977* followed by treatment with TGF-β (10 ng/mL). **E** Co-IP assays of CBP or P300 with phosphorylated SMAD3 Ser423/425 in A549 cells, which were transfected with *LINC01977* followed by treatment with TGF-β (10 ng/mL), and treated with RNase inhibitor or RNase. **F** Represent images of H3K27ac, assayed by IF confocal microscopy in A549 cells treated with TGF-β (10 ng/mL). Actin, Phalloidin (red); nuclei, DAPI (blue); and H3K27ac (green). Scale bar: 20 μm. (**G, H**) ChIP assays suggested that *LINC01977* SE (**G**) and promoter (**H**) were co-occupied by CBP, P300 and H3K27ac. **I** Abilities of invasion and migration were diminished by combination of *LINC01977*-ASO and inhibition of CBP/P300, SGC-CBP30 (5 μM) in vitro. **J** Schematic representation of lung metastasis nude mouse model. **K** Representative images of IVIS detection and metastatic loci in the lung derived from mouse model. **L** Mouse overall survival after tail-vein injection. **M** H&E stained and phosphorylated SMAD3 Ser423/425, CBP, and ZEB1 IHC detection of serial sections from lung metastasis nude mouse model. Representative images are shown. H&E, scale bars: 500 μm; IHC, scale bars: 50 μm. Data in C, G–I are representative of three independent experiments and presented as mean ± S.D., *n* = 3 biologically independent samples, and the *P* value was determined by a two-tailed unpaired Student’s *t* test and one-way ANOVA, respectively. *P ≤ 0.05; **P ≤ 0.01; ***P ≤ 0.001; n.s. not significantly

CREB-binding protein (CBP) and P300 (CBP/P300), transcriptional co-activators, have been reported to bind to SMAD3 and enhance SMAD-induced transactivation of target genes [32]. In this study, we identified that CBP/P300 was among the listed proteins of the quantitative LC/MS assays in which *LINC01977* RNA pulled-down (Fig. 3H, Additional file 2: Table S3). Subsequently, OmiXcore was used to predict the interaction between CBP/P300 and *LINC01977* (Fig. 5A). Both RNA pull-down and RIP results validated the interaction (Fig. 5B, C). To assess the impact of *LINC01977* on SMAD3/CBP/P300, we performed co-IP assays in LUAD cells (Fig. 5D, Additional file 1: Figure S6A-C). The results revealed that *LINC01977* promoted the interaction between p-SMAD3 and CBP/P300 upon TGF-β stimulation. Next, we repeated the co-IP assays, where RNase was used to decrease *LINC01977* expression and RNase inhibitor was used to protect *LINC01977* from degradation. Unsurprisingly, the absence of *LINC01977* significantly abolished the interaction between p-SMAD3 and CBP/P300, while the interaction between CBP and P300 was unchanged in LUAD cells (Fig. 5E, Additional file 1: Figure S6D, E). As a well-known histone acetyltransferase (HAT), CBP/P300 is primarily responsible for acetylating H3K27 and regulating gene transcription [33]. To investigate whether *LINC01977* affects histone modification, in situ IF assays were performed to detect the transcriptional activation marker H3K27Ac level, which suggested that high expression of *LINC01977* indeed resulted in the acetylation of H3K27 (Fig. 5F). Furthermore, to verify whether the transcription of *LINC01977* could benefit from CBP/P300 and induce H3K27Ac, we performed ChIP-qPCR for detection. Interestingly, similar to the SMAD3 occupancy (Fig. 4I–K), co-occupancy of CBP/P300 and H3K27Ac was also detected in the SE (Fig. 5G, Additional file 1: Figure S6F, G) and promoter region of *LINC01977* (472–480 and 1466–1477) (Fig. 5H, Additional file 1: Figure S6H, IL). Taken collectively, these observations demonstrated that SE-*LINC01977* not only participated in the TGF-β/SMAD3 pathway in LUAD cells, but also leveraged the *LINC01977*-dependent SMAD3/CBP/P300 epigenetic transcriptional complex.

Recently, a growing number of small molecule inhibitors of key epigenetic regulators and nucleic acid drugs have been tested in cancer clinical trials [34]. Thus, we attempted to explore whether SGC-CBP30, the small molecule inhibitor of CBP/P300, could delay the malignant progression of LUAD. In vitro functional experiments suggested that combination therapy consisting of *LINC01977*-ASO and SGC-CBP30 significantly decreased LUAD cells migration and invasion (Fig. 5I, Additional file 1: Figure S6J-L). To better understand the effect of combination therapy in LUAD, the metastatic lung colonization model was used to mimic the in vivo environment (Fig. 5J). The results revealed that combination therapy resulted in remarkable suppression of lung metastasis, while SGC-CBP30 or *LINC01977*-ASO alone also produced inhibitory effects (Fig. 5K). Moreover, combination therapy-treated mice had significantly longer morbidity-free survival (Fig. 5L). Furthermore, consistent with tumor suppression, combination therapy significantly reduced p-SMAD3 and CBP levels in xenograft tumor mouse models compared with single-agent treatment (Fig. 5M). Thus, these results indicated that a significant therapeutic advantage was found by combining SGC-CBP30 with *LINC01977*-ASO compared with either agent alone.

### *LINC01977*-dependent SMAD3/CBP/P300 complex epigenetically up-regulates *ZEB1*, the central switch of EMT process, in LUAD cells

**Fig. 6:**
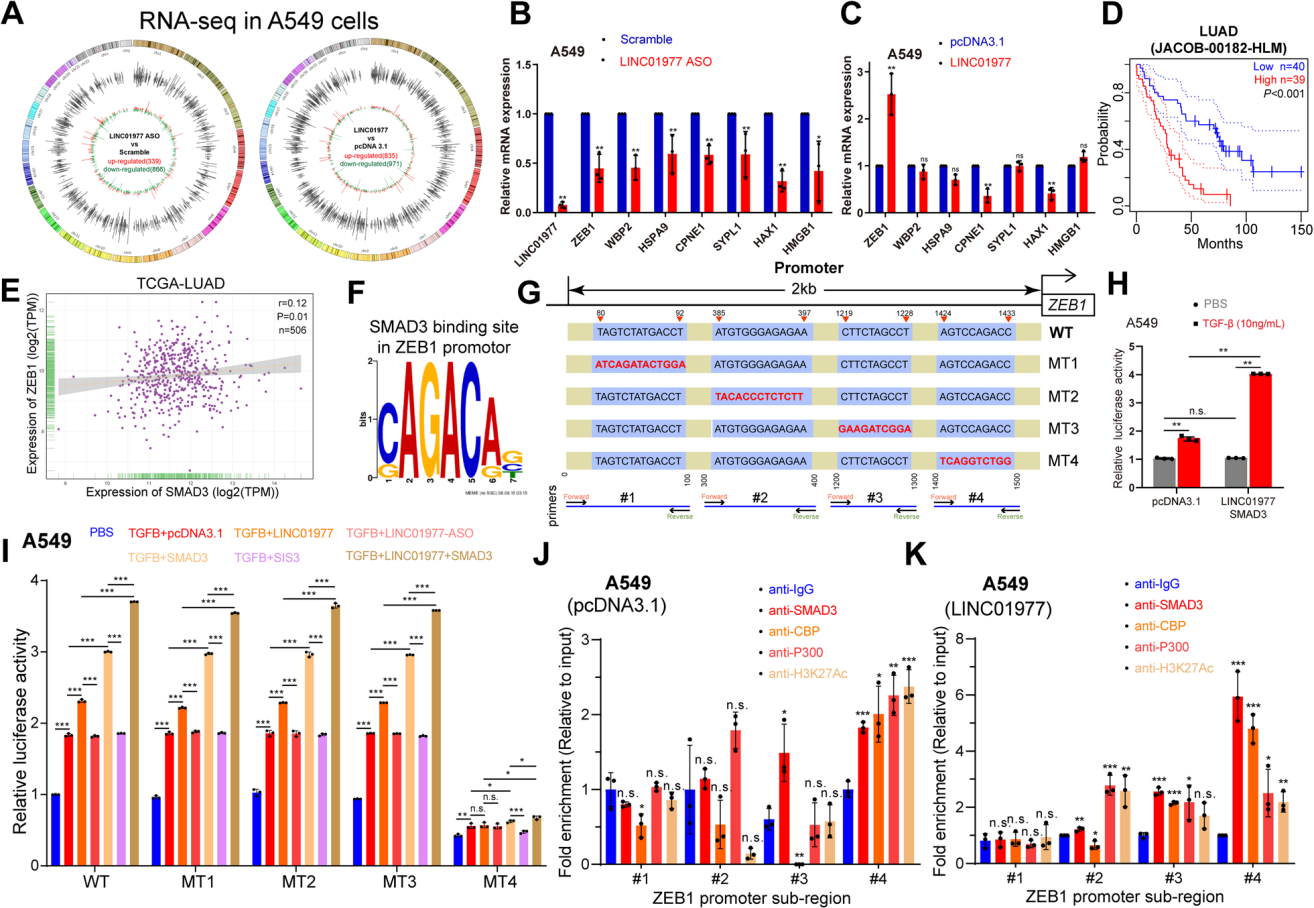
*LINC01977*-dependent SMAD3/CBP/P300 complex epigenetically up-regulates *ZEB1*, the central switch of EMT process, in LUAD cells. **A** The circos plots showing dysregulated RNAs found in RNA-seq analysis in A549 cells, up-regulated (pcDNA3.1 vs *LINC01977*) and down-regulated (*LINC01977*-ASO vs Scramble). **B**, **C** Significantly dysregulated genes were validated by qRT-PCR in A549 cells. **D** Kaplan–Meier survival analysis of ZEB1 expression in LUAD patients from JACOB-00812-HLM dataset. **E** Correlation analysis of *SMAD3* and *LINC01977* in TCGA-LUAD dataset. **F** SMAD3-binding motif analysis was performed on ZEB1 promoter via JASPAR database. (**G**) Schematic representation of SMAD3-binding sites on ZEB1 promoter and primers design strategy. **H** Transcription activity of ZEB1 was elevated in A549 cells treated with TGF-β (10 ng/mL), and significantly promoted by transfected with *LINC01977* and SMAD3 followed by treatment with TGF-β (10 ng/mL) as indicated. (**I**) Mutant-binding site #4 (MT4) diminished the transcriptional activity of *LINC01977* in A549 cells. Transcriptional activity of *LINC01977* was elevated by treated with TGF-β (10 ng/mL) and promoted by overexpression of *LINC01977* or SMAD3, also decreased by absent of *LINC01977* or SMAD3. **J** ChIP assays suggested that only #4 sub-region of ZEB1 promoter was co-occupied by CBP, P300, SMAD3 and H3K27ac. (**K**) ChIP assays suggested that co-occupancy of #4 sub-region of ZEB1 promoter by CBP, P300, SMAD3, and H3K27ac was promoted by *LINC01977* overexpression. Data in A-C and H–K are representative of three independent experiments and presented as mean ± S.D., *n* = 3 biologically independent samples, and the *P* value was determined by a two-tailed unpaired Student’s *t* test and one-way ANOVA, respectively. *P ≤ 0.05; **P ≤ 0.01; ***P ≤ 0.001; n.s. not significantly

To determine the major pathways affected by *LINC01977*, we performed RNA-seq analysis in A549 cells to explore the disturbed transcriptome affected by *LINC01977* and to identify the potential downstream targets. There were 835 up-regulated genes (*LINC01977* vs pcDNA3.1) and 866 down-regulated genes (*LINC01977*-ASO vs scramble) (Fig. 6A, Additional file 2: Table S4). Analysis from Kyoto Encyclopedia of Genes and Genomes (KEGG) showed that highly expressed *LINC01977* was associated with cancer, signal transduction, and transport and catabolism (Additional file 1: Figure S7A). Gene Ontology (GO) enrichment analysis also revealed that *LINC01977* was associated with protein binding and nuclear processes (Additional file 1: Figure S7B). By screening overlapping differentially expressed genes and qRT-PCR validation, *ZEB1* was noted to have a consistent correlation tendency with *LINC01977* expression (Fig. 6B, C). Among these genes, ZEB1 was the most attractive candidate based on the following: (i) ZEB1, the core transcriptional inducer of EMT, is a key factor for cell plasticity and cancer metastasis [35], (ii) ZEB1 maintains cell fate and stem cell quiescence [36], and (iii) its elevated expression in poor-prognosis LUAD (Fig. 6D). We therefore proceeded to examine whether ZEB1 was the downstream target gene of SE-*LINC01977* in LUAD cells.

A positive correlation between *SMAD3* and *ZEB1* was observed in the LUAD public dataset (Fig. 6E). Moreover, motif analysis indicated that the SMAD3 specific binding motif, SMAD-binding element (SBE), was found in the ZEB1 promoter region by JASPAR (Fig. 6F). According to the identified binding sites, we constructed wild-type (WT) and mutant (MT1-MT4) luciferase reporter gene plasmids (Fig. 6G). The results revealed that transcription of *ZEB1* was promoted by TGF-β, and it was profoundly elevated by *LINC01977*-SMAD3 overexpressed together, indicating that *LINC01977*-SMAD3 played an important role in the transcriptional activation of *ZEB1* (Fig. 6H). Subsequently, to clarify which SMAD3-binding site plays the major role in *ZEB1* regulation, we performed luciferase activity assays, which suggested that a mutation in binding site #4 directly abolished the regulation from TGF-β/*LINC01977*-SMAD3 to *ZEB1* (Fig. 6I, Additional file 1: Figure S7C, D). Furthermore, ChIP-qPCR results also provided evidence that the *LINC01977*-dependent SMAD3/CBP/P300 complex co-occupied the wild-type binding site #4, along with increased acetylation at H3K27 (Fig. 6J, Additional file 1: Figure S7E, F). With *LINC01977* overexpression in A549 cells, these co-occupancies were enhanced together (Fig. 6K). Additionally, to further validate whether loss-of chaperon proteins attenuate ZEB1 expression, we employed CRISPR/Cas9 system to knockout SMAD3, CBP and P300 in LUAD cells. Gene editing efficiency were verified by PCR amplification (Additional file 1: Figure S7G, H). With *LINC01977*-overexpressed LUAD cells, loss-of chaperon proteins significantly decreased *ZEB1* expression, suggesting that *LINC01977*-mediated transcriptional regulation of ZEB1 was dependent on *LINC01977*-SMAD3/CBP/P300 complex (Additional file 1: Figure S7I). Furthermore, as an EMT activator, ZEB1 plays a key role in cell plasticity and promotes metastasis of cancers. EMT is also associated with greater metastatic potential [37]. High expression of *ZEB1* was associated with the EMT pathway activity by GSCA bioinformatic analysis (Additional file 1: Figure S7J). To confirm the impact of *LINC01977* on the EMT process, we performed qRT-PCR of EMT-associated genes and observed that *LINC01977* promoted EMT in LUAD cells (Additional file 1: Figure S7K). Subsequently, we detect EMT and cell cycle related proteins expression in gain- or loss- of *LINC01977* LUAD cells. The results of western blot assays suggested that *LINC01977* promoted EMT and cell cycle progression (Additional file 1: Figure S7L). Additionally, we performed IHC analysis in tumor tissues we obtained from subcutaneous model and xenograft metastasis model for in vivo validation. As expected, *LINC01977* deficiency decreased p-SMAD3, ZEB1, Ki-67, and PCNA expression; meanwhile, expression of epithelial marker E-cadherin and apoptosis marker cleaved caspase 3 was elevated, which revealed that *LINC01977* promotes EMT and proliferation in vivo (Additional file 1: Figure S7M). Taken together, these results established an axis for the SE-*LINC01977*-dependent SMAD3/CBP/P300 complex that epigenetically up-regulated *ZEB1*, thereby promoting the EMT process via *ZEB1* in LUAD cells.

### *LINC01977* is a promising predictor of poor outcome in early-stage LUAD

**Fig. 7:**
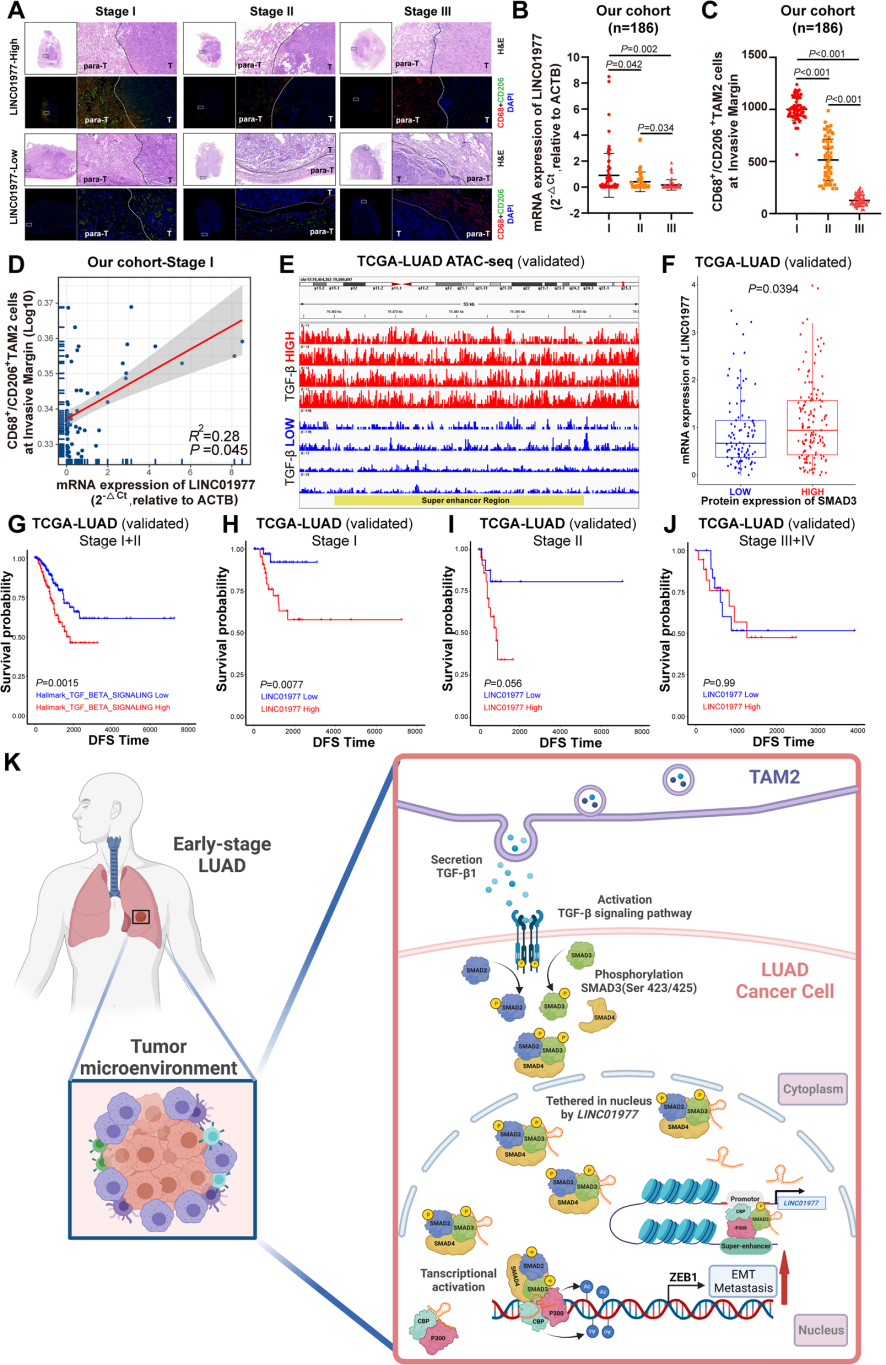
*LINC01977* is a promising predictor of poor outcome in early-stage LUAD. **A** Representative H&E and immunofluorescence staining serial sections of patients with LUAD clinical stage I, II, and III. TAM2 was significantly high infiltrated at invasive margin in early-stage LUAD (stage I) patients. CD68, red; CD206, green; and nuclei, DAPI, blue. CD68^+^/CD206^+^ indicated TAM2. Scale bars: 200 μm. **B** Relative expression of *LINC01977* detected by qPCR from different clinical stage of LUAD in our cohort. **C** For all patients in our cohort, shown is the number of TAM2 around invasive margin (y-axis) versus the different LUAD clinical stages (x-axis). **D** Correlation analysis of *LINC01977* expression by qRT-PCR detection and infiltrated TAM2 numbers in our cohort. (**E**) ATAC-seq analysis of super-enhancer region in different expression of TGF-β in TCGA-LUAD dataset. **F** In TCGA-LUAD dataset, patients with high protein expression of SMAD3 were also with high expression of *LINC01977*. **G** Kaplan–Meier analysis of the DFS in LUAD clinical stage I + II patients with different TGF-β signaling pathway hallmarks levels, as determined using TCGA-LUAD dataset. **H–J** Kaplan–Meier analysis of the DFS in LUAD clinical stage I (**H**), II (**I**) and III + IV (**J**) patients with different *LINC01977* expression levels, as determined using TCGA-LUAD dataset. **K** Graphic abstract. Figure in K was created using BioRender. Data in A and F are representative of three independent experiments and presented as mean ± S.D., *n* = 3 biologically independent samples, and the *P* value was determined by a two-tailed unpaired Student’s *t* test and one-way ANOVA, respectively

To investigate the correlation between *LINC01977* and TAM2 infiltration, the most abundant stromal cell population in LUAD, we assessed the expression of *LINC01977* and TAM2 using serial sections in a cohort of LUAD tissues (*n* = 186) coupled with qRT-PCR and immunofluorescence staining of CD68^+^/CD206^+^, respectively (Fig. 7A). Interestingly, high *LINC01977* expression was observed in early-stage LUAD, and TAM2 were significantly enriched at the invasive margin (Fig. 7B, C). Moreover, these results were validated in TCGA-LUAD dataset (Additional file 1: Figure S5K). Correlation analysis demonstrated that *LINC01977* was positively correlated with TAM2 infiltration in early-stage LUAD (Fig. 7D). As TAM2-induced TGF-β signaling pathway activation, higher chromatin accessibility in the SE region was observed in LUAD patients with high expression of TGF-β by ATAC-seq analysis from TCGA-LUAD (Fig. 7E). Additionally, the *LINC01977* mRNA level in LUAD patients with high SMAD3 protein expression was similarly elevated in TCGA-LUAD dataset (Fig. 7F). Moreover, high-TAM2 infiltration-induced TGF-β signaling pathway activation was associated with poor prognosis in early-stage LUAD, which was also validated in TCGA-LUAD dataset (Fig. 7G). Taken collectively, these results indicated that TAM2 were highly infiltrated at the invasive margin, which was correlated with the high expression of *LINC01977*.

To explore the correlation between *LINC01977* expression and the clinical-pathological date in LUAD, we performed disease-free survival (DFS) analysis in the TCGA-LUAD dataset, which revealed that high expression of *LINC01977* was significantly associated with poor prognosis in early-stage LUAD (Stage I) (Fig. 7H), whereas the predictive power decreased with advanced clinical stage (Fig. 7I, J). Collectively, we speculated that *LINC01977* functioned as a biomarker of poor prognosis in early-stage LUAD.

## Discussion

Numerous epigenetic studies have reported that aberrantly activated SEs play important roles in tumorigenesis [38]. Recent studies have reported that aberrantly activated cell type-specific SEs exert oncogenic effects [39]. Thus, we focused on early-stage LUAD malignant progression and explored how SE-associated epigenetic alterations dictate the response and adaptive behavior of LUAD cells. Here, we identified a SE-lncRNA *LINC01977*, which is driven by corresponding SE, that was also identified to be a cancer-testis gene. Interestingly, our results revealed that SE-induced *LINC01977* transcriptional activation was the specific intrinsic response to the adaptation of the tumor microenvironment with high-TAM2 infiltration in early-stage LUAD. Mechanistically, *LINC01977* was involved in the canonical TGF-β/SMAD3 signaling pathway by binding and tethering SMAD3 in the nucleus, thereby forming the SMAD3/CBP/P300 transcriptional activation complex that up-regulated ZEB1. In both the TCGA-LUAD dataset and our cohort, we observed high-TAM2 infiltration at the invasive margin and high expression of *LINC01977* in early-stage LUAD. Similarly, TAM2-induced secretion of TGF-β and signaling pathway activation were also associated with poor prognosis in early-stage LUAD. Thus, our observations provide an intrinsic epigenetic mechanism in early-stage LUAD cells for adapting and reaping the benefits of the extrinsic tumor microenvironment (Fig. 7K).

SEs are tissue- and cell-specific regulatory regions of DNA consisting of clusters of activated enhancers [22]. In our previous study, we identified aberrantly activated SEs that promoted LUAD malignant progression by establishing core transcriptional circuitry with master transcription factors. Recently, increasing evidence has suggested that functional lncRNAs were dysregulated by aberrantly activated SEs in cancer, which are known as SE-lncRNAs [13, 14, 40]. Although SE-lncRNAs have been the focus of intense interest, it remains largely unexplored whether SE-lncRNAs are important for tumorigenesis or epigenetic plasticity associated with malignant tumor progression in LUAD. Based on the growing appreciation of epigenetic regulation by SEs, we set out to explore dysregulated SE-lncRNAs in LUAD. Herein, we identified a SE-lncRNA, *LINC01977*, also defined as a cancer-testis gene in LUAD. Moreover, in situ tissue microarray by RNAscope and mRNA level quantification by qRT-PCR revealed that *LINC01977* was highly expressed in LUAD tissues. According to the H3K27Ac and H3K4me1 ChIP-seq data from ENCODE database, we identified cell type specific and aberrantly activated SE that regulated *LINC01977* in the A549 cell line. Similar to other SE-lncRNA studies, Hi-C analysis and luciferase reporter assays further validated this finding. Taken collectively, these observations suggested that *LINC01977* was an SE-associated cancer-testis lncRNA in LUAD.

The TGF-β signaling pathway controls multiple cell fate decisions to promote tissue homeostasis and malignant progression [41]. TGF-β-induced EMT is associated with metastasis in a variety of cancers, including LUAD [42]. However, the profound impact of the TGF-β/SMAD3 signaling pathway during cancer metastasis has not been addressed. In the present study, we demonstrate that *LINC01977* facilitates the nuclear translocation of SMAD3 and provide evidence of the establishment of the SMAD3/CBP/P300 transcriptional activation complex in LUAD at the epigenetic level. Blocking the interaction between *LINC01977* and SMAD3 by *LINC01977*-ASO specifically inhibited TGF-β/SMAD3 signal transduction, which was manifested as an inhibition of SMAD3 phosphorylation and subsequent nuclear translocation. As an indispensable transcription factor in TGF-β-induced transcriptional regulation, the SMAD3-binding motif was observed in both the SE and *LINC01977* promoter, indicating that *LINC01977* not only assisted TGF-β/SMAD3 signal transduction but also elevated its expression. These results reveal the mechanism by which aberrantly activated SE-*LINC01977* harnesses the cancer cell’s intrinsic canonical machinery addicted to TGF-β/SMAD3 pathway in LUAD.

Until now, in clinical trials, therapies targeting TGF-β in NSCLC have mostly focused on antibodies [43]. Although inhibiting the TGF-β signaling pathway is considered to possess therapeutic potential in NSCLC, the clinical efficacy is disappointing. The results of our study indicate that *LINC01977* expression is very low in human bronchial epithelial cells and immune cells in the tumor microenvironment, while it is highly expressed in A549 cells, particularly upon TGF-β stimulation. *LINC01977* bound to the MH2 functional domain of SMAD3 and tethered in the nucleus to facilitate SMAD3 accumulation in the nucleus. Moreover, *LINC01977* enhanced the interaction between SMAD3 and co-activator CBP/P300 to epigenetically up-regulate ZEB1, thereby promoting metastasis. Additionally, the combination of *LINC01977*-ASO and SGC-CBP30 was significantly better than monotherapy in prolonging the survival of mice. Unsurprisingly, CBP/P300 and induced H3K27Ac were observed in the SE and *LINC01977* promoter, which was similar to a feedback loop, partly explaining the TGF-β-induced *LINC01977* elevation. Hence, these results establish that the response of cancer cells upon extrinsic TGF-β stimulation was not unique, but rather it took advantage of the intrinsically activated SE to adopt the TGF-β/SMAD3 signaling pathway for regulating the tumor microenvironment and metastasis in LUAD.

Increasing evidence indicates that macrophages control the tumor microenvironment of a variety of cancers, in that they secrete cytokines and chemokines for remodeling the microenvironment [44]. TAM2, an important source of TGF-β, was identified as a pro-tumoral subtype [27]. In the present study, we utilized a co-culture model to mimic the high infiltration of TAM2 and the subsequent high level of TGF-β. Our study revealed that TAM2-induced enhancement of malignant progression was dependent on *LINC01977* and TGF-β. Furthermore, high *LINC01977* expression and high-TAM2 abundance were simultaneously observed at the invasive margin in early-stage LUAD, which was significantly associated with poor prognosis. Taken collectively, these results reveal that the up-regulation of SE-*LINC01977* in early-stage LUAD may be a risk factor for tumor metastasis and may serve as a therapeutic target.

In this study, we also reveal that the active SE upstream of *LINC01977* did not only hijack *LINC01977* to synergistically enhance the canonical TGF-β/SMAD3 signaling pathway, but also responded to the rich TGF-β microenvironment secreted by the infiltrated TAM2. SE hijacking *LINC01977* promote TGF-β/SMAD3-mediated crosstalk between the intrinsic characteristics of cancer and the extrinsic characteristics of the tumor microenvironment. A previous study has reported that cancer-associated fibroblasts (CAFs) display distinct changes in DNA methylation, particularly at enhancers and promoters, compared to nonmalignant fibroblasts [45]. Ovarian cancer maintains high expression of miR-101 and miR-26a, which constrained expression of EZH2 in T cells, to dampen the function of CD8^+^ T cells [46]. The major determinant of dynamic crosstalk between the cancer epithelium and the surrounding microenvironment should be explored in the further.

## Conclusions

In summary, our study showed that SE-*LINC01977* was a promising predictor of early-stage LUAD metastasis and poor disease-free survival. Mechanistically, *LINC01977* was involved in the TGF-β/SMAD3 signaling pathway by binding and tethering SMAD3 in the nucleus to form the SMAD3/CBP/P300 transcriptional activation complex for the epigenetic up-regulation of ZEB1. Additionally, high *LINC01977* expression was SE-induced and enhanced by TGF-β via a feedback mechanism. Our study uncovered the intrinsic response of cancer cells to extrinsic high-TAM2 infiltration was not only due to the activation of the TGF-β signaling pathway, but rather took full advantage of aberrantly activated SE to reap the benefits of the tumor microenvironment for metastasis in early-stage LUAD.

## Supplementary Information

**Additional file 1:** Supplementary Figures.

**Additional file 2:** Supplementary Tables.

## Data Availability

All data used in this work can be acquired from the TCGA database (http://cancergenome.nih.gov/), ENCODE database (http://encodeproject.org/), CCLE database (http://sites.broadinstitute.org/ccle), and ImmLnc database (http://bio-bigdata.hrbmu.edu.cn/ImmLnc/). The lncRNA sequencing data of LUAD samples have been deposited in the Gene Expression Omnibus (GEO) under accession number GSE196584. The processed data are available from the corresponding author upon reasonable request.

## Abbreviations

lncRNAs: Long non-coding RNAs
*LINC01977*: Long intergenic non-protein coding RNA 1977
SE: Super-enhancer
LUAD: Lung adenocarcinoma
NSCLC: Non-small cell lung cancer
ChIP-seq: Chromatin immunoprecipitation followed by next-generation sequencing
IF: Immunofluorescence
FISH: Fluorescence in situ hybridization
SMAD3: SMAD family member 3
CBP: CREB-binding protein
P300: E1A-binding protein p300
RNA-seq: RNA sequencing
ZEB1: Zinc finger E-box-binding homeobox 1
TME: Tumor microenvironment
TAM2: M2-like tumor-associated macrophage
