# Supplementary material for: Super-enhancer hijacking LINC01977 promotes malignancy of early-stage lung adenocarcinoma addicted to the canonical TGF-β/SMAD3 pathway
Source: J Hematol Oncol. 2022 Aug 18;15:114. doi: 10.1186/s13045-022-01331-2 (PMC9389757; doi:10.1186/s13045-022-01331-2)
Supplement: Supplementary file 1 — Additional file 1: Supplementary Figures. [file 13045_2022_1331_MOESM1_ESM.pdf]

Super-enhancer hijacking *LINC01977* promotes malignancy of early-stage lung adenocarcinoma addicted to the canonical TGF-β/SMAD3 pathway

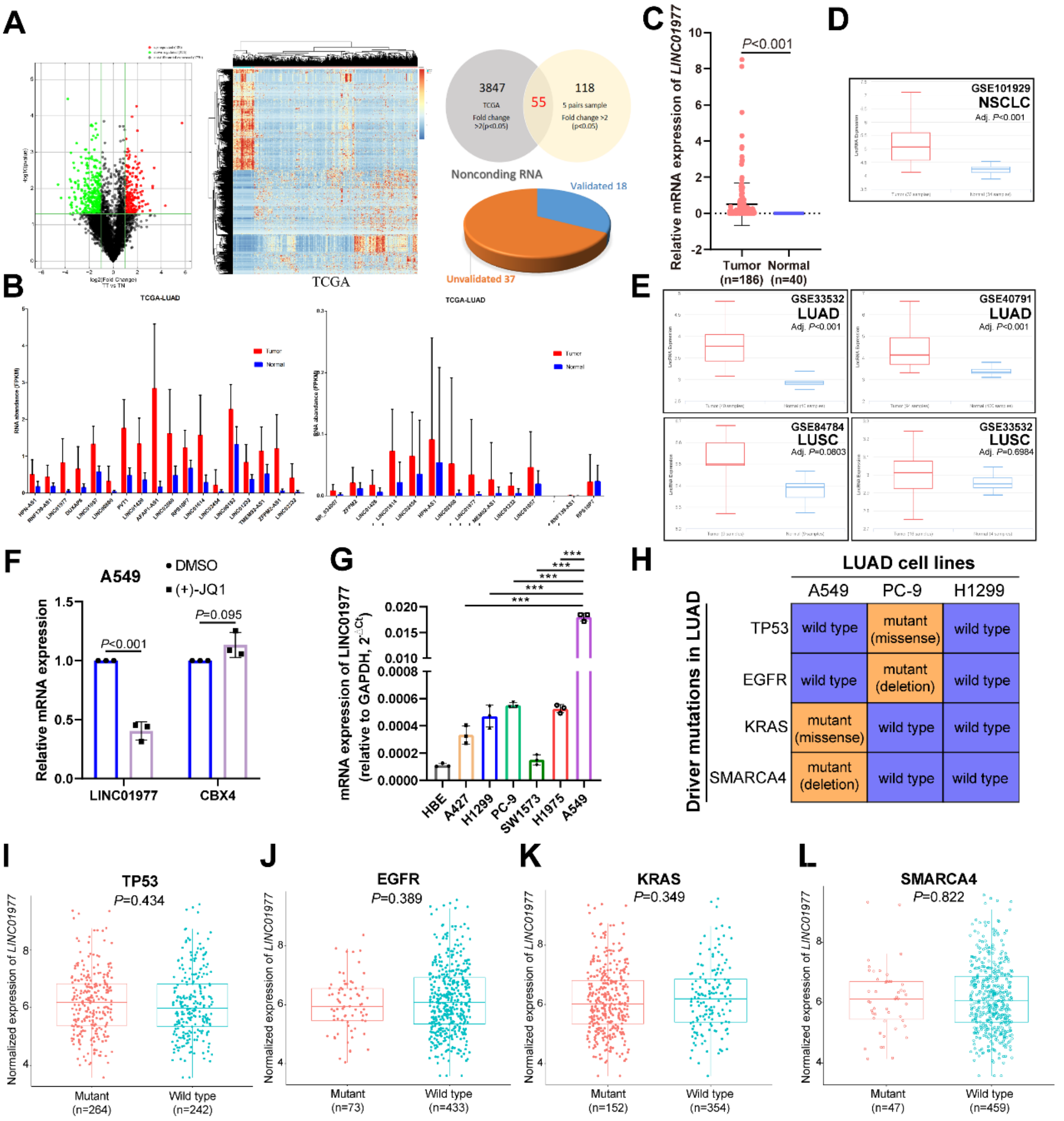

Supplementary Figure 1.

(**A**) Volcano plot of gene expression microarray results. The x-axis shows fold change between normal and LUAD samples and the y-axis shows one-way ANOVA *P* value; points corresponding to one-fold change cutoff and *P*<0.05 are colored red

(increasing) and green (decreasing), respectively (left panel). Heatmap representing the relative expression level of the differentially expressed genes from microarray (middle panel). Flow chart of gene selection (right panel). (**B**) Expression of candidate lncRNAs in LUAD tumors and normal lung tissue from the TCGA database. (**C**) qRT-PCR detection of *LINC01977* expression in LUAD (validation set n=186, 40, respectively) and normal tissues. (**D**) Expression analysis of *LINC01977* in NSCLC from Gene Expression Omnibus (GEO) database www.ncbi.nlm.nih.gov/geo, accession number: GSE101929. (**E**) Expression analysis of *LINC01977* in LUAD (upper panel, accession number: GSE33532, GSE40791) and LUSC (lower panel, accession number: GSE84784, GSE33532) from GEO database. (**F**) qRT-PCR detection of *LINC01977* and *CBX4* expression treated with (+)-JQ1 (5 μM) for 1 hour. (**G**) qRT-PCR detection of *LINC01977* expression in LUAD cancer cell lines. (**H**) Mutation status of LUAD cell line. Correlation analysis between mutation status and LINC01977 expression in TCGA-LUAD dataset, including TP53 (**I**)**,** EGFR (**J**), KRAS (**K**) and SMARCA4 (**L**). Data in F and G are representative of three independent experiments and presented as mean ± S.D., n=3 biologically independent samples, the *P* value was determined by a two-tailed unpaired Student's *t* test and one-way ANOVA, respectively. *$P \leq 0.05$; **$P \leq 0.01$; ***$P \leq 0.001$; n.s. not significantly.

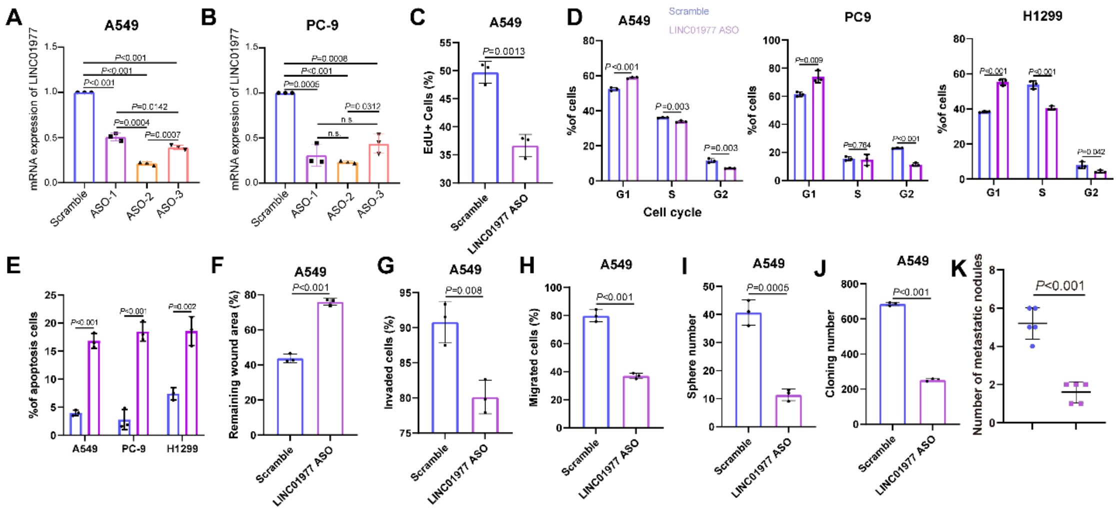

Supplementary Figure 2.

The efficiency of ASO knockdown. A549 (**A**) and PC-9 (**B**) cells were transfected with the indicated ASO, and the expression of *LINC01977* was analyzed by qRT-PCR. (**C**) Quantitative analysis of EdU assays in Figure 2A. (**D**) Quantitative analysis of cell cycle assay using flow cytometry in LUAD cell lines in Figure 2B. (**E**) Quantitative analysis of cell apoptosis assay using flow cytometry in LUAD cell lines in Figure 2C. (**F**) Quantitative analysis of wound healing assay in Figure 2D. (**G**) Quantitative analysis of invasion assay in Figure 2D. (**H**) Quantitative analysis of migration assay in Figure 2D. (**I**) Quantitative analysis of sphere-forming assay in Figure 2D. (**J**) Quantitative analysis of colony-forming assay in Figure 2D. (**K**) Lung metastatic nodules were assessed. Data point in represent (mean ± SEM) are shown from n=5 biologically independent samples by two-sided unpaired t test. Data in A-J are representative of three independent experiments and presented as mean ± S.D., n=3 biologically independent samples, the *P* value was determined by a two-tailed unpaired Student's *t* test and one-way ANOVA, respectively.

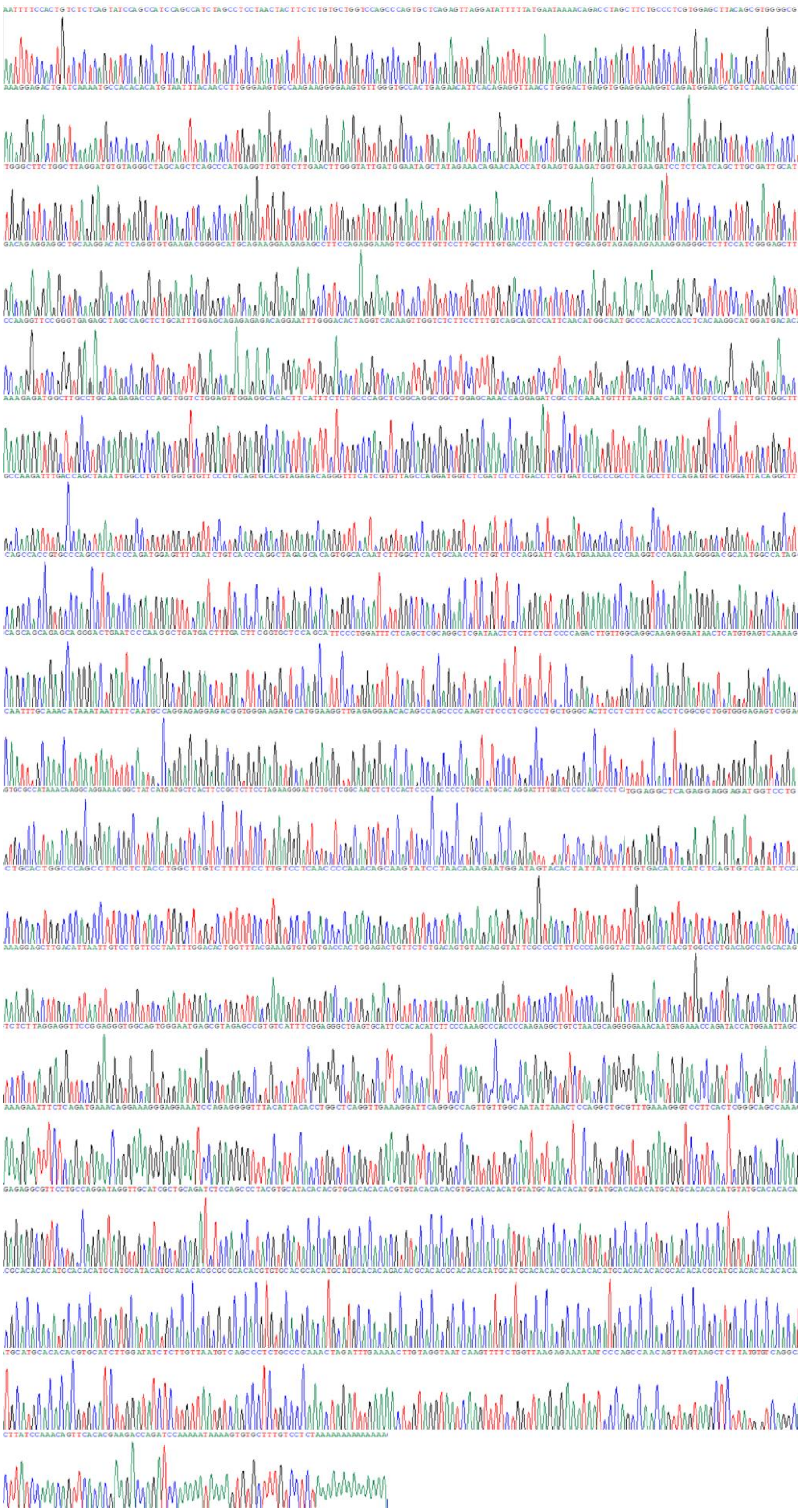

Supplementary Figure 3.

Full length sequence of LINC01977 was shown.

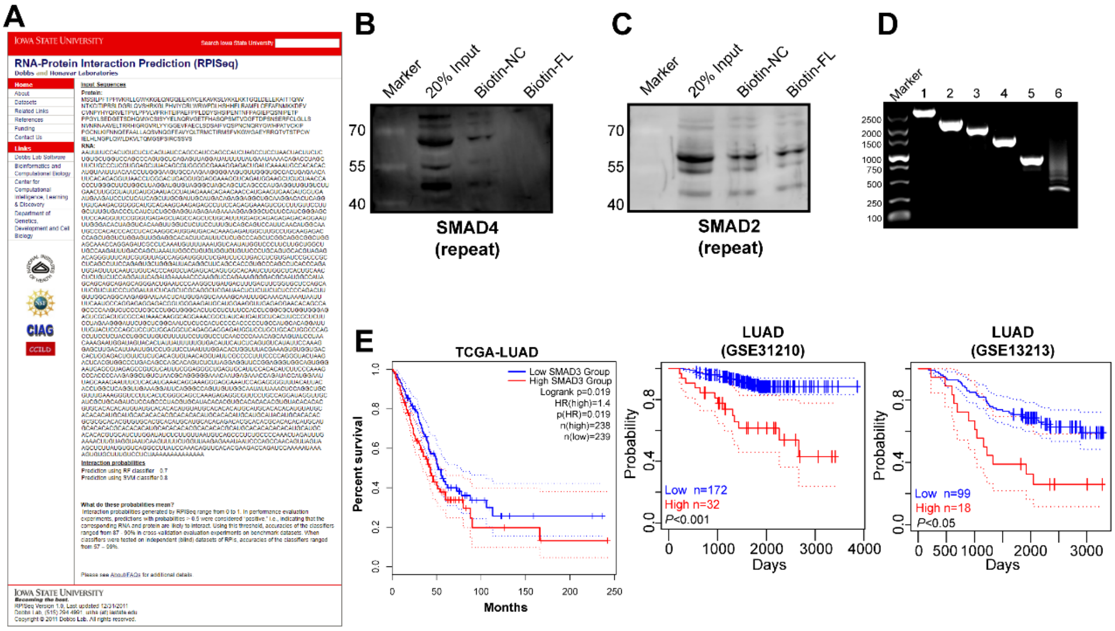

Supplementary Figure 4.

(**A**) *LINC01977*-SMAD3 interaction predicted by catRAPID. (**B**) RNA pull-down assays were performed using in vitro transcribed biotinylated full length *LINC01977* and antisense (as negative control, Biotin-NC). Western blot was used to evaluate the interaction between *LINC01977* and SMAD4 (**B**), and also interaction between *LINC01977* and SMAD2 (**C**). Repeat assays of Figure 3K. (**D**) Representation images of the full length *LINC01977* and the truncated RNAs. (**E**) Kaplan-Meier analysis of the overall survival (OS) in LUAD patients with different SMAD3 expression levels, as determined using TCGA-LUAD dataset (left panel), GSE31210 (middle panel) and GSE13213 (right panel) from GEO database.

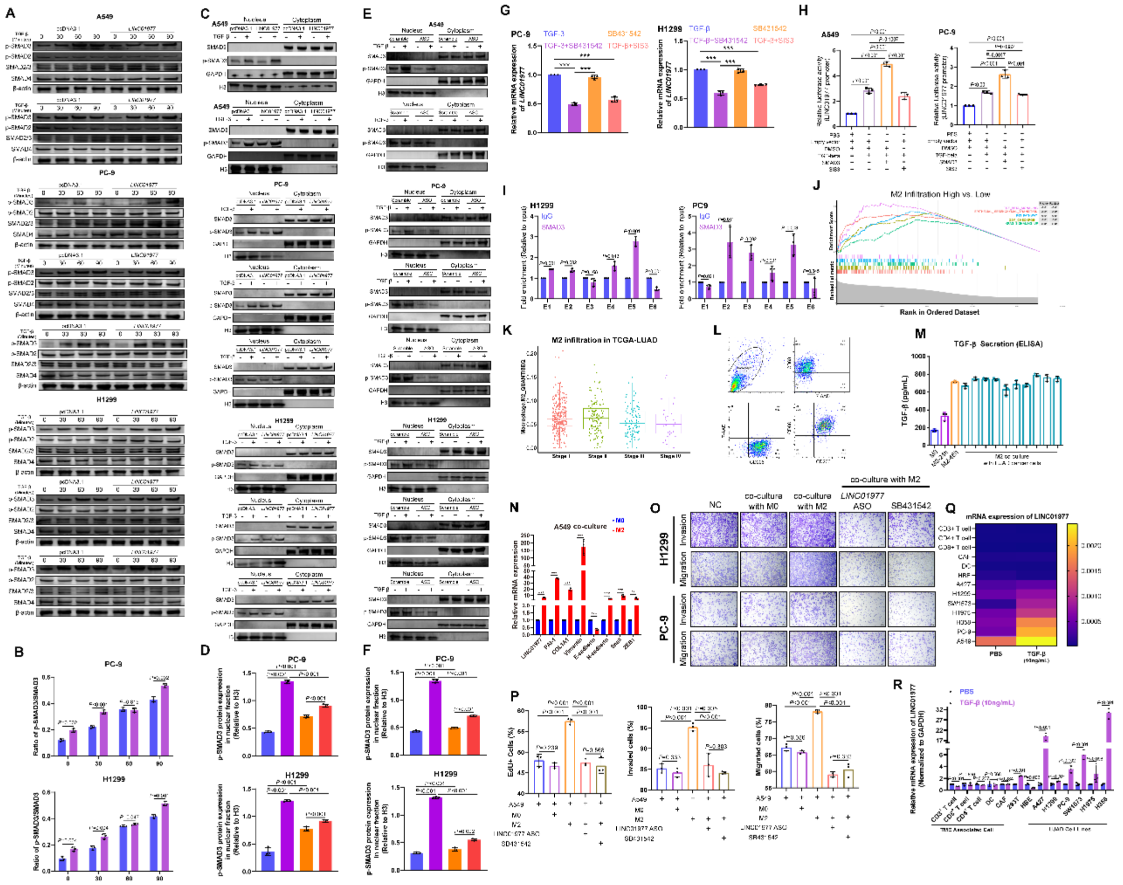

Supplementary Figure 5.

(**A**) Western blot detection of phosphorylation SMAD3 Ser423/425 in LUAD cells. Repeat assays of Figure 4A. (**B**) Quantitation of expression of p-SMAD3 in PC-9 (up panel) and H1299 (down panel) cells. (**C**) Western blot detection of phosphorylation SMAD3 Ser423/425 in nucleoplasm and cytoplasm fractions in LUAD cells. Repeat assays of Figure 4C. (**D**) Quantitation of proteins expression level in PC-9 (up panel) and H1299 (down panel) cells. (**E**) Western blot detection of phosphorylation SMAD3 Ser423/425 in nucleoplasm and cytoplasm fractions in LUAD cells. Repeat assays of Figure 4D. (**F**) Quantitation of proteins expression level in PC-9 (up panel) and H1299 (down panel) cells. (**G**) qRT-PCR detection of *LINC01977* expression in PC-9 (left panel) and H1299 cells (right panel) treated with TGF-β (10 ng/mL), TGF-β inhibitor SB431542 (10 μM) and p-SMAD3 inhibitor SIS3 (5 μM) as indicated. Repeat assays of Figure 4H. (**H**) Dual-luciferase reporter assays were performed in LUAD cells to detect impact of TGF-β and p-SMAD3 on LINC01977 transcription. (**I**) ChIP assays suggested that SMAD3 bound to the *LINC01977* SE in H1299 (left panel) and PC-9

(right panel). Repeat assays of Figure 4K. (**J**) Pathway enrichment analysis suggested that highly infiltration of M2-like macrophage was positively correlated with activation of TGF-β signaling pathway and epithelial mesenchymal transition in LUAD. The pathway analysis was performed with GSEA method, which was based on an empirical permutation test procedure. (**K**) Infiltration of M2-like macrophages in different clinical stage of LUAD was validated in TCGA-LUAD dataset. (**L**) Validation of THP-1 derived M2-like macrophage in co-culture model by flow cytometry. (**M**) The total TGF-β concentration of the supernatants was measured by the ELISA assays. (**N**) qRT-PCR detection of *LINC01977* expression and downstream EMT progress associated marker gene in M2 co-cultured A549 cells. (**O**) LINC01977 dependency invasion and migration assays in M2 co-culture model in H1299 (up-panel) and PC-9 (down-panel). Repeat assays of Figure 4O. (**P**) Quantitative analysis of EdU (left panel), invasion (middle panel) and migration (right panel) assays in Figure 4O. (**Q**) Heatmap shows alteration of *LINC01977* expression according to qRT-PCR results. (**R**) qRT-PCR detection of *LINC01977* expression in TME associated cells and LUAD cell lines treated with PBS or TGF-β (10 ng/mL). Data in B, D, F-I and M-R are representative of three independent experiments and presented as mean ± S.D., n=3 biologically independent samples, the *P* value was determined by a two-tailed unpaired Student's *t* test and one-way ANOVA, respectively. **P*≤0.05; ***P*≤0.01; ****P*≤0.001; n.s. not significantly.

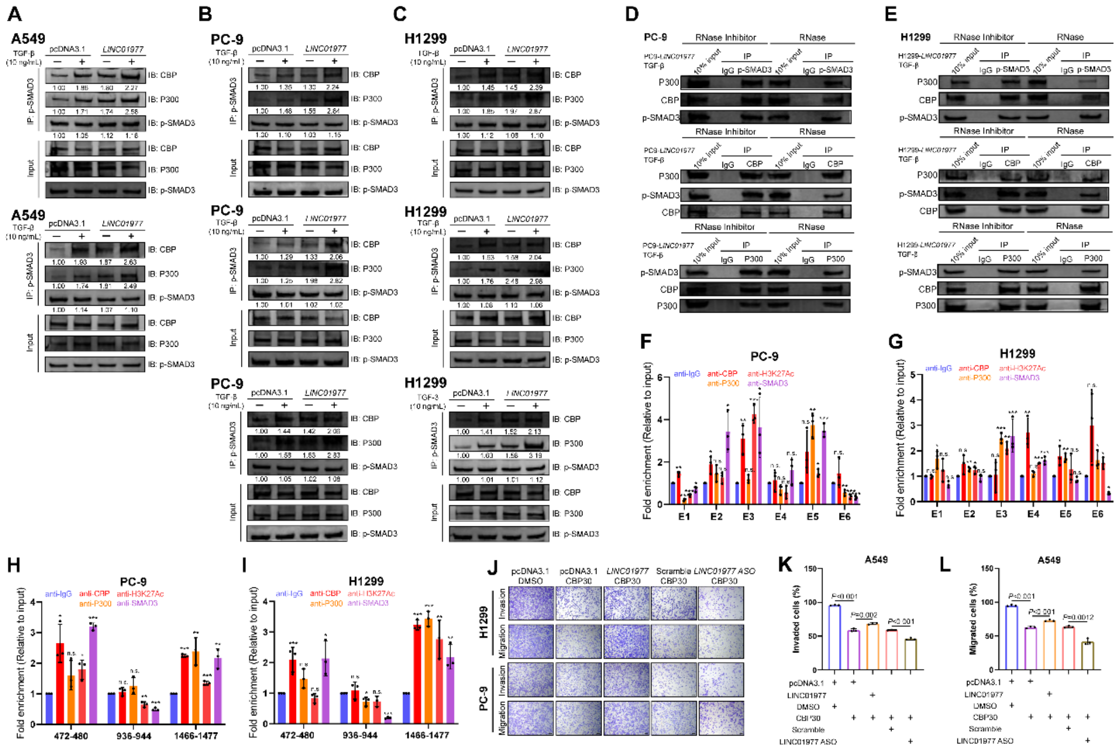

Supplementary Figure 6.

(**A-C**) Co-IP assays were performed in A549 (**A**), PC-9 (**B**) and H1299 (**C**) cells. Repeat assays of Figure 5D. (**D-E**) LINC01977 dependency IP assays were performed in PC-9 (**D**) and H1299 (**E**) cells. (**F, G**) ChIP assays suggested that *LINC01977* SE were co-occupied by CBP, P300, H3K27ac and SMAD3 in PC-9 (**F**) and H1299 (**G**) cells. (**H, I**) ChIP assays suggested that *LINC01977* promoter were co-occupied by CBP, P300, H3K27ac and SMAD3 in PC-9 (**H**) and H1299 (**I**) cells. (**J**) Invasion and migration assays were performed in loss-of LINC01977 and/or loss-of CBP/P300 in PC-9 and H1299 cells. Repeat assays of Figure 5I. (**K**) Quantitative analysis of invasion assay in Figure 5I. (**L**) Quantitative analysis of migration assay in Figure 5I. Data in F-I are representative of three independent experiments and presented as mean ± S.D., n=3 biologically independent samples, the *P* value was determined by a two-tailed unpaired Student’s *t* test and one-way ANOVA, respectively.

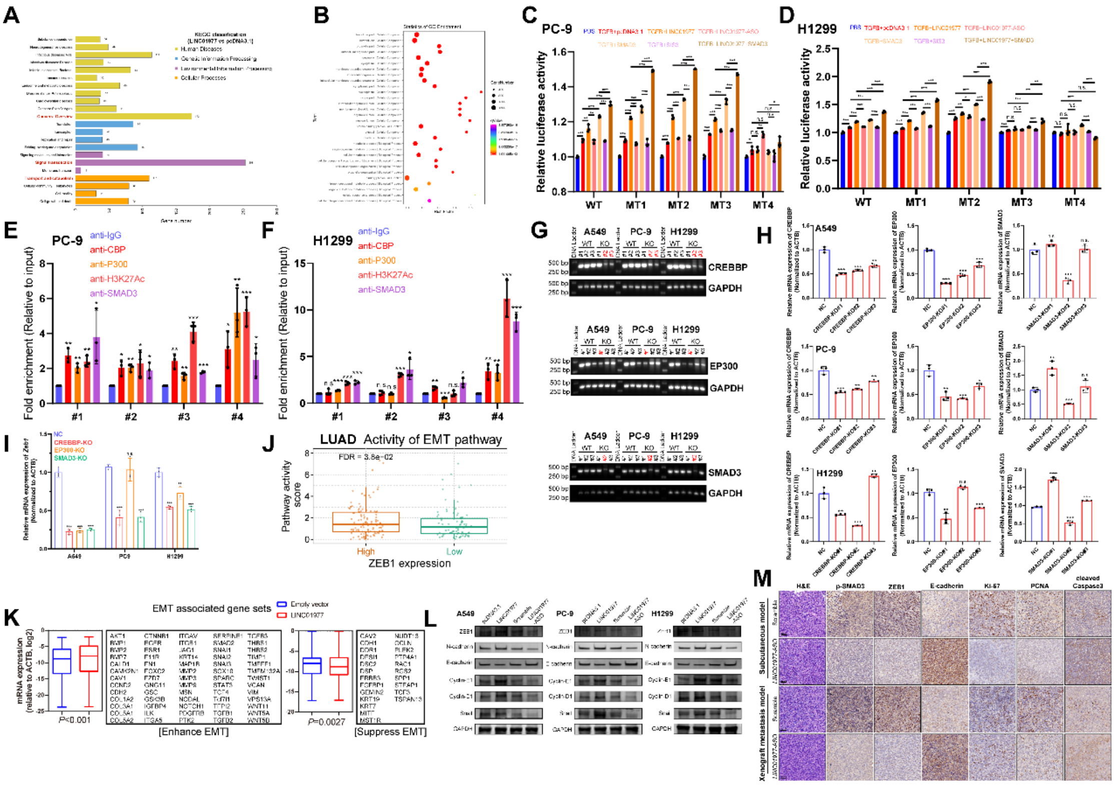

Supplementary Figure 7.

(**A**) KEGG pathway analysis of *LINC01977* overexpression induced upregulated genes from RNA-seq in A549 cells. (**B**) GO analysis based on *LINC01977* overexpression RNA-seq data. (**C-D**) Mutant binding site #4 (MT4) diminished the transcriptional activity of *ZEB1* in PC-9 (**C**) and H1299 (**D**) cells. Transcriptional activity of ZEB1 was elevated by treated with TGF-β (10 ng/mL) and promoted by overexpression of *LINC01977* or SMAD3, also decreased by absent of *LINC01977* or SMAD3. (**E-F**) ChIP assays suggested that co-occupancy of #4 sub-region of ZEB1 promoter by CBP, P300, SMAD3, H3K27ac and SMAD3 was promoted by *LINC01977* overexpression in PC-9 (**E**) and H1299 (**F**) cells. (**G, H**) Efficiency of gene editing by Crispr/Cas9 system were validated by PCR amplification with DNA electrophoresis gel (**G**) and qRT-PCR (**H**). (**I**) qRT-PCR detection of ZEB1 expression in LINC01977-overexpressed LUAD cells with CBP, P300 and SMAD3 knockout by Crispr/Cas9 system. (**J**) High expression of ZEB1 is associated with EMT pathway activity. (**K**) PCR array detected alteration of EMT associated genes in A549 cells transfected with empty vector (pcDNA3.1) or *LINC01977*. (**L**) EMT and cell-cycle related proteins expression in gain- or loss-of *LINC01977* LUAD cells. (**M**) IHC detection of

proliferation and apoptosis marker proteins on tumor tissues obtained from subcutaneous model and xenograft metastasis model. Data in C-F, H, I and K are representative of three independent experiments and presented as mean ± S.D., n=3 biologically independent samples, the *P* value was determined by a two-tailed unpaired Student's *t* test and one-way ANOVA, respectively.
