# Supplementary material for: Super-enhancer hijacking LINC01977 promotes malignancy of early-stage lung adenocarcinoma addicted to the canonical TGF-β/SMAD3 pathway
Source: J Hematol Oncol. 2022 Aug 18;15:114. doi: 10.1186/s13045-022-01331-2 (PMC9389757; doi:10.1186/s13045-022-01331-2)
Supplement: Supplementary file 2 — Additional file 2: Supplementary Tables. [file 13045_2022_1331_MOESM2_ESM.pdf]

Supplementary Table 1

Clinicopathological characteristics of LUAD patients for SE associated lncRNA microarray										
**Patient**	**Age (year)**	**Gender**	**Tobacco use**	**Alcohol use**	**Postoperative Therapies**	**Tumor size**	**Tumor location**	**N stage**	**Clinical stage**	**Prognosis**
#1	54	female	No	No	No	1.5×1×1cm	Right; middle	N2	IIIA	N/A
#2	65	female	No	No	No	3×3×3cm	Left; lower	N2	IIIA	Brain metastasis
#3	62	female	No	No	Targeted therapy	3×3×3cm	Right; lower	N2	IIIA	Bone metastasis
#4	66	female	No	No	Radiotherapy+Chemotherapy	3×3×1cm	Right; lower	N2	IIIA	Bone metastasis
#5	61	female	No	No	Radiotherapy+Chemotherapy	3×1.5×1.5cm	Left; lower	N2	IIIA	Recurrent

Supplementary Table 2

Relationship between *LINC01977* expression levels and clinicopathological parameters of LUAD				
**Variable**	**No. of patients**	***LINC01977* expression**		***P* value**
		**High expression**	**Low expression**	
**Age (yr)**				
＞60	53	36	17	0.056
≤ 60	45	22	23	
**Sex**				
Female	43	28	15	0.291
Male	55	30	25	
**Clinical stage**				
Early (I)	33	21	12	**0.039**
Advanced (II-IV)	65	27	38	
**pT status**				
T1-T2	72	42	30	0.776
T3-T4	26	16	10	
**Lymph node metastasis**				
No metastasis (N0)	54	29	25	0.221
Metastasis (N1-N3)	44	29	15	

Supplementary Table 3

Band 50-55kDa LC/MS rank TOP100

Rank	Accession	Sum PEP Score	Coverage	Unique peptides	Score Sequest HT
1	P84022	1581.538369	95.17647059	60	4959.066099
2	P05787	1467.599725	94.82401656	66	4697.421585
3	P06733	1022.505164	91.24423963	49	4057.320155
4	P08729	606.6390208	85.5010661	43	1233.621121
5	P05783	771.4372129	84.88372093	40	896.1322623
6	P43686	298.5300061	83.97129187	30	310.8965957
7	P50395	499.7216777	81.57303371	40	482.51165
8	P00352	976.3556712	80.03992016	54	4410.315665
9	Q71U36	465.6282355	77.38359202	1	1196.528094
10	Q9BQE3	422.0409281	77.28285078	3	1319.375438
11	Q07960	151.0121453	75.62642369	24	135.925935
12	P07437	700.7892453	75.45045045	6	2199.131603
13	P68371	675.644767	75.28089888	2	1955.705238
14	P06576	694.2272743	74.48015123	29	1073.580579
15	P04350	514.8481644	71.84684685	4	1477.078957
16	Q9Y230	228.9457727	70.84233261	32	248.3533123
17	P43490	358.3485188	68.83910387	40	448.7293661
18	P26641	195.8826669	68.42105263	31	318.9674957
19	P68104	366.6728514	68.18181818	16	1012.378626
20	Q13885	612.8343401	68.08988764	5	1779.80046
21	Q9BUF5	328.5890574	67.48878924	14	913.1560061
22	Q13509	537.4840734	67.33333333	11	1232.049487
23	Q13561	120.8725664	65.33665835	17	108.7333139
24	P08670	145.9376298	65.02145923	25	158.7312125
25	P67809	270.5204192	64.81481481	15	308.2512472
26	P14618	410.8626275	64.40677966	35	462.6313863
27	P68366	405.7176081	64.28571429	9	1042.744968
28	P25705	409.086521	64.01446655	38	667.2947152
29	P50454	237.6768535	63.87559809	24	205.6634455
30	P11413	213.8892378	63.49514563	32	227.0782813
31	Q9Y265	247.0443594	63.37719298	28	242.6316487
32	P17980	227.3181357	62.87015945	25	213.5278629
33	P00390	259.6424879	62.83524904	21	239.7500737
34	P34897	158.5821594	62.5	20	163.1026411
35	Q96KP4	209.7828911	61.47368421	27	184.2945125
36	P07099	283.0689945	61.0989011	36	328.7398981
37	P05455	298.2244595	60.78431373	33	341.5806321
38	O00232	175.4943917	60.74561404	31	174.4490408
39	P60709	133.9092579	60.26666667	9	133.9913621
40	Q16851	193.1511364	60.03937008	27	212.5914408

41	P07237	167.9285824	59.64566929	30	141.3539716
42	P31943	255.3024444	58.12917595	11	394.2249269
43	P29401	397.350523	58.105939	33	488.8759105
44	P0DPH8	403.8282418	58	1	899.1663804
45	O14745	229.2535312	57.54189944	17	182.6606115
46	P05091	246.5583595	57.05996132	28	587.6586207
47	P51648	164.2247418	56.90721649	25	195.5416422
48	P61158	135.3239151	55.98086124	20	142.0798564
49	Q9HDC9	192.6886156	55.76923077	20	205.2852856
50	Q16401	141.4431492	55.75396825	19	115.6625829
51	Q16658	107.8498187	54.96957404	20	105.1846702
52	P35998	157.0643385	54.27251732	23	177.7481421
53	P28838	118.2976828	54.14258189	23	114.5122209
54	P30838	229.1924475	53.86313466	28	427.0858424
55	P38919	155.7968916	53.77128954	22	161.5511703
56	P36578	263.9550258	53.62997658	31	332.1872935
57	P26196	141.305102	53.00207039	18	115.6942887
58	P52209	313.5134955	52.58799172	29	462.5774032
59	P22570	121.9887003	52.54582485	21	117.7349184
60	P09104	107.1313298	52.07373272	11	183.706032
61	P39656	128.9165862	51.97368421	13	134.9735388
62	Q15084	227.4570122	51.36363636	22	264.0062671
63	P48637	122.5985433	51.05485232	22	129.4728945
64	P49419	120.0953226	50.83487941	21	113.1074994
65	P26599	140.0634251	50.65913371	15	132.7507082
66	P52597	133.6086641	49.87951807	13	179.826232
67	P30101	156.0647737	49.5049505	21	168.5456184
68	P61011	122.008846	49.4047619	22	115.2772222
69	O00303	98.59998454	49.29971989	12	99.94163728
70	O43852	68.34183326	49.20634921	12	69.21605921
71	Q05639	185.1140958	49.02807775	7	621.6798221
72	Q16181	221.5184502	48.51258581	24	192.4682437
73	P00367	238.6284628	48.38709677	10	260.6780126
74	Q9NP81	121.3947004	48.06949807	19	107.2598009
75	P14868	124.6744556	47.50499002	20	107.4988172
76	P06744	143.8662924	47.49103943	18	135.6718684
77	Q9BY77	68.16267214	47.26840855	16	69.08964586
78	P78371	84.10965482	47.10280374	19	73.56762373
79	P63151	102.3007249	46.30872483	12	91.21495628
80	Q9UNH7	90.04749135	46.30541872	16	70.56385732
81	Q8NBS9	79.90121087	46.2962963	15	72.10716057
82	P16989	111.6714183	46.23655914	7	112.079877
83	Q16719	117.645636	46.02150538	14	104.4194913
84	P30837	188.5006967	45.84139265	21	311.6115426

85	P12081	114.1357245	45.77603143	15	104.0805347
86	Q16543	126.0619245	45.23809524	19	99.02100313
87	P55795	129.3096152	44.98886414	7	203.2322404
88	P61201	78.56425251	44.69525959	18	72.91095686
89	P20042	91.33526609	44.14414414	18	77.35656643
90	O60701	108.9941424	44.12955466	19	102.2766018
91	Q96CS3	84.17457461	44.04494382	16	73.67514265
92	P41091	142.7559189	43.43220339	18	138.9869863
93	Q16555	105.1121487	42.83216783	15	92.10396981
94	Q86U42	43.5729608	42.81045752	7	39.725945
95	Q12849	80.34523384	42.70833333	11	67.71130705
96	P55036	94.34220477	42.70557029	12	96.68012142
97	P30566	120.1280722	42.3553719	15	115.1415908
98	Q9NVA2	71.89638486	41.95804196	8	63.50963116
99	P31153	105.0859204	41.7721519	18	131.3928148
100	P23381	55.47472531	41.61358811	13	47.27001011

Band >180kDa LC/MS rank TOP100

Rank	Accession	Sum PEP Score	Coverage	Unique peptides	Score Sequest HT
1	Q92793	1803.856985	60.49140049	291	1812.831102
2	Q15149	1758.483976	62.10503843	288	1639.385664
3	Q14204	1709.319109	55.31640121	240	1617.377473
4	Q09666	1641.43132	73.03904924	260	1376.559812
5	O75369	961.3061178	64.14296695	115	898.7437736
6	Q13813	946.972705	60.67961165	133	790.1794667
7	P78527	945.9886118	43.33817829	173	919.1473345
8	P21333	929.2240149	57.83906309	111	905.261104
9	Q00610	855.6265952	53.97014925	64	916.5940317
10	Q01082	839.1267628	55.24534687	114	657.6525495
11	P46821	749.4049395	46.636953	78	627.3870324
12	P14618	617.6134858	73.06967985	41	1221.2778
13	P49327	611.4623591	45.16129032	85	560.3857783
14	P68104	519.548195	66.45021645	16	1700.154776
15	O75643	491.536421	43.58614232	79	424.2212335
16	Q63HN8	451.6718237	24.65911273	103	379.8824241
17	Q7Z6Z7	444.6999196	31.48148148	89	396.4861609
18	Q5T4S7	433.4121175	21.76345746	74	330.3679854
19	P27708	424.1689803	43.37078652	66	375.6921833
20	P16615	419.9469428	42.61036468	46	445.7852045
21	P35579	398.8981198	42.19387755	60	287.49968
22	P12270	384.5969754	38.46804909	69	305.5215521
23	Q9UPN3	384.4338541	17.74499188	89	300.2841051
24	P33527	382.0963012	43.82756368	52	335.9909056
25	P29401	363.7877843	59.87158909	31	515.9851829
26	Q08211	355.5013404	46.69291339	55	347.4090531
27	Q8IVF2	351.5707354	37.03192407	76	276.8472172
28	Q13085	342.951355	36.53026428	64	291.0387753
29	Q6P2Q9	338.5325621	36.40256959	68	264.9832737
30	P04264	329.2455713	57.91925466	34	285.8656027
31	Q709C8	320.5093516	28.16413536	76	253.4285573
32	P04844	298.1986369	46.27575277	25	276.9590974
33	P39023	296.7105826	58.06451613	28	285.3654655
34	Q9Y490	294.9980488	31.44431326	48	200.4540907
35	P20020	292.9605376	32.70491803	21	270.1534002
36	P53621	291.6058824	54.82026144	53	257.0299079
37	P13645	278.3914675	58.04794521	25	238.8555722
38	P05023	274.9791681	42.71749756	27	235.2273804
39	P02751	269.995107	28.58340319	42	224.844698
40	Q9Y5B9	269.7178979	43.07545368	41	247.6863607
41	Q14789	262.7641224	29.30346732	66	229.7035779

42	Q15393	254.4714414	38.29087921	39	254.3007993
43	P46940	253.5309009	38.8050694	49	205.2569982
44	P49792	241.0372113	25.46526055	44	195.0334867
45	Q05639	240.225189	61.77105832	9	848.1393415
46	Q92616	235.4920469	25.53350805	51	185.7995476
47	Q16719	221.3494651	58.70967742	19	261.9317133
48	Q9HC35	208.7915805	46.78899083	39	180.9755973
49	P00352	208.5533743	61.67664671	25	251.0773394
50	Q16531	192.5504976	35.1754386	35	163.8218123
51	P35606	188.1612872	45.14348786	33	164.8376588
52	P55011	187.8240031	28.71287129	27	162.4606856
53	O15439	186.4102177	30.64150943	31	126.1083587
54	P06748	186.1273361	39.79591837	13	175.9117537
55	P17987	175.6646584	58.09352518	22	154.749806
56	Q01813	174.3137255	42.98469388	28	148.3747308
57	P04406	170.1325714	70.44776119	19	165.8533708
58	P46777	170.1123837	64.30976431	24	208.4349533
59	P46939	168.323027	17.06961841	39	129.9866006
60	Q8TEM1	166.3293766	22.36354001	31	148.9230709
61	P12236	164.8074991	60.40268456	4	265.0807129
62	P08238	163.9660311	37.01657459	13	130.8950449
63	P35908	163.10335	50.23474178	22	158.6334952
64	Q8N766	162.3398586	30.51359517	25	142.2479825
65	O95573	161.1624922	45.97222222	23	124.8825266
66	P35527	160.1516534	44.14125201	19	144.7794143
67	P11413	157.0714877	52.42718447	26	159.6546441
68	Q16881	156.9974777	35.43913713	18	111.4449326
69	Q14839	156.5011974	27.61506276	29	141.7653925
70	P53675	153.0253025	11.15853659	1	166.4952459
71	Q14914	152.8980084	72.6443769	21	145.8187681
72	Q5JPE7	150.3729567	34.17521705	1	111.4677333
73	Q14690	149.1937778	22.7151256	34	127.925053
74	Q15155	148.0211232	35.02454992	1	109.3912809
75	Q92887	144.732706	25.63106796	28	126.2644082
76	Q9UQ35	144.7184182	12.71802326	23	126.1715105
77	P05141	143.6968699	55.03355705	7	247.848453
78	O14975	142.0421688	42.25806452	22	121.6202908
79	O15438	140.7521216	19.05697446	21	106.3919255
80	P60842	140.7368077	51.97044335	16	126.624578
81	P23634	140.5954545	14.74617244	2	133.1459945
82	Q8WXH0	136.934572	8.104575163	42	108.5068802
83	O75083	135.8228682	49.5049505	23	117.1415695
84	O60271	129.7328041	28.91748675	29	115.0455855
85	P15924	128.933626	15.98746082	35	117.8888229

86	P33992	127.9052756	41.55313351	22	106.8069786
87	P12235	127.4048642	46.97986577	2	205.7315415
88	P23141	126.8138527	46.91358025	20	108.6303782
89	Q9NR30	126.7913906	40.74074074	25	114.4817839
90	Q9NR09	126.3635232	10.62384188	34	93.89195669
91	Q93008	125.5239456	16.22568093	36	94.49911678
92	P26038	124.7426353	53.72616984	32	105.6293902
93	Q99460	122.6980175	32.84365163	25	96.68458593
94	Q9NQC3	122.0192493	13.75838926	10	122.6737167
95	P17858	119.1084712	29.74358974	15	94.02234197
96	P14868	118.6864876	49.7005988	24	112.5047536
97	Q14764	118.0773501	33.03471445	20	89.71740127
98	P69905	117.9239084	64.08450704	8	197.5144657
99	Q02952	116.5288145	22.671156	22	81.14891481
100	P07900	113.8727792	31.83060109	11	100.9044868

Supplementary Table 4

RNA-seq differentially genes (LINC01977 vs pcDNA3.1)

Symbol	Ensembl	logFC	*P* value
LINC01977	ENSG00000262772	13.6774669	0
MUC5B	ENSG00000117983	-4.431091641	3.411E-236
HNRNPH2	ENSG00000126945	5.60782489	5.062E-202
NOTCH3	ENSG00000074181	-3.183899457	1.077E-152
EGR1	ENSG00000120738	3.65163124	1.122E-141
TM4SF20	ENSG00000168955	-3.044779989	5.99E-131
PDK4	ENSG00000004799	-3.176174961	1.313E-122
CDH1	ENSG00000039068	-2.969539032	5.013E-122
MUC5AC	ENSG00000215182	-2.498009647	2.503E-107
FCGBP	ENSG00000275395	-2.608217303	4.121E-101
CPS1	ENSG00000021826	-2.530582301	5.945E-100
FOSL1	ENSG00000175592	2.83177313	2.053E-99
TM4SF4	ENSG00000169903	-2.571348657	4.873E-99
GADD45A	ENSG00000116717	2.91660856	4.2225E-98
ALOXE3	ENSG00000179148	4.52348671	1.0365E-94
CP	ENSG00000047457	-2.483203885	1.7324E-93
STC2	ENSG00000113739	2.55708222	9.9624E-88
LAMC2	ENSG00000058085	2.47223649	1.1252E-85
ASB1	ENSG00000065802	2.75010994	1.4017E-81
CPLX2	ENSG00000145920	-2.160347124	1.6997E-81
CYP24A1	ENSG00000019186	-2.129855049	8.0333E-79
DDIT3	ENSG00000175197	2.60898445	2.4786E-77
NEB	ENSG00000183091	-4.067231959	1.4139E-75
GPX2	ENSG00000176153	-2.290303262	2.073E-74
GREM1	ENSG00000166923	3.02373945	4.4908E-73
ERBB3	ENSG00000065361	-3.200815862	5.0304E-71
RHOV	ENSG00000104140	-3.626031533	8.4655E-71
SAMD11	ENSG00000187634	-3.172624897	1.157E-69
SESN2	ENSG00000130766	2.37309572	8.3989E-68
CFH	ENSG00000000971	-2.416682684	8.8358E-68
SLC45A4	ENSG00000022567	-2.088263827	8.1545E-67
JUP	ENSG00000173801	-1.969555206	2.7916E-64
NTS	ENSG00000133636	-2.265669376	1.1163E-62
PPP1R15A	ENSG00000087074	2.02826209	1.1801E-61
ETS1	ENSG00000134954	2.08924925	2.4076E-61
SLC7A7	ENSG00000155465	-2.182557720	2.419E-61
SLC23A2	ENSG00000089057	-1.907767967	1.216E-60
SPDEF	ENSG00000124664	-3.360250180	1.3338E-60
UBE2F-SCLY	ENSG00000258984	-7.543791100	2.4895E-59
MTUS1	ENSG00000129422	-1.853393718	4.3372E-57
HSPA5	ENSG00000044574	1.8878731	1.1766E-55
CYP1A1	ENSG00000140465	4.29256064	1.3526E-55
RPL36A-HNR	ENSG00000257529	-3.900173459	1.3808E-55
HSPA6	ENSG00000173110	4.00508562	8.8303E-55
SNAP25	ENSG00000132639	-2.177557677	1.2817E-54
ANXA13	ENSG00000104537	-3.230281662	5.4767E-54
AL163636.2	ENSG00000259171	-4.695422044	2.9048E-53
FN1	ENSG00000115414	-1.800929423	3.9994E-53
ATF3	ENSG00000162772	2.03062987	6.8252E-53
TNFRSF12A	ENSG00000006327	1.87646604	3.1571E-52
TNS4	ENSG00000131746	-1.783615482	1.235E-51
C5	ENSG00000106804	-2.271054922	2.5044E-51

ANKRD1	ENSG00000148677	4.01258798	3.3748E-51
JUN	ENSG00000177606	1.83896711	7.6005E-51
ALDH1A1	ENSG00000165092	-1.632738240	3.0612E-49
SMOC1	ENSG00000198732	-1.940965909	1.5142E-48
BCAM	ENSG00000187244	-2.600325243	1.9071E-48
CELSR2	ENSG00000143126	-2.197632852	2.7054E-48
TOMM6	ENSG00000214736	3.4035577	2.2018E-46
DLX2	ENSG00000115844	4.12570116	5.8642E-46
THRA	ENSG00000126351	-1.953439492	6.8172E-46
KLF6	ENSG00000067082	1.74967663	1.9875E-45
DHCR24	ENSG00000116133	-1.666215324	4.2178E-45
FAM129A	ENSG00000135842	1.98110562	5.0732E-45
VCAN	ENSG00000038427	-1.800813425	7.3043E-45
CLCF1	ENSG00000175505	1.79841786	2.6279E-44
SLC12A2	ENSG00000064651	-1.740990686	3.8445E-44
HK2	ENSG00000159399	3.21074832	5.8525E-44
LINC00473	ENSG00000223414	-2.153077806	1.4726E-43
DOK4	ENSG00000125170	-1.867324651	1.8852E-43
AEN	ENSG00000181026	1.68347355	4.26E-43
MEGF9	ENSG00000106780	-1.619442891	5.3802E-43
MYORG	ENSG00000164976	-1.996205600	5.5264E-43
TJP3	ENSG00000105289	-3.897938674	1.5593E-42
SERTAD1	ENSG00000197019	1.97500077	2.4896E-42
MRC2	ENSG00000011028	-1.617258782	2.6542E-42
FGFR4	ENSG00000160867	-1.855845025	5.8677E-42
CDC25B	ENSG00000101224	-1.547136660	6.0207E-42
FRMD6	ENSG00000139926	2.06769046	8.09E-42
ZEB1	ENSG00000148516	4.3517946	4.14E-43
FGB	ENSG00000171564	-3.586195793	1.4944E-41
KIAA1551	ENSG00000174718	1.84297599	5.2915E-41
ABL2	ENSG00000143322	1.66855268	1.0079E-40
TRIML2	ENSG00000179046	1.76544189	1.1006E-40
C3	ENSG00000125730	-1.611061945	6.5838E-40
FILIP1	ENSG00000118407	-2.465071644	1.2619E-39
ABCG1	ENSG00000160179	-2.260062008	6.0424E-39
FGA	ENSG00000171560	-3.555221541	7.2182E-39
KRT80	ENSG00000167767	1.63827035	8.8235E-39
TCIM	ENSG00000176907	-1.759564776	1.0146E-38
HR	ENSG00000168453	-1.629325876	1.3891E-38
GEM	ENSG00000164949	1.88814759	1.657E-38
ANKS4B	ENSG00000175311	-3.478182972	3.3776E-38
HNF4A	ENSG00000101076	-1.949739832	1.0992E-37
TBX3	ENSG00000135111	2.0362148	1.1735E-37
DKK1	ENSG00000107984	-1.553763253	2.0136E-37
OAS1	ENSG00000089127	-1.813416743	2.641E-37
DNAL1	ENSG00000119661	2.43422434	5.4325E-37
TNFRSF10D	ENSG00000173530	1.62813927	1.0912E-36
HGD	ENSG00000113924	-2.461249929	1.1505E-36
PPIF	ENSG00000108179	1.60834777	1.9878E-36
KDM6B	ENSG00000132510	1.59065945	2.2488E-36
DUSP8	ENSG00000184545	2.06671995	2.5407E-36
PLK2	ENSG00000145632	1.60862466	3.164E-36
KIF21B	ENSG00000116852	-1.675252620	4.065E-36
DNAJB2	ENSG00000135924	1.73830311	5.7509E-36
LPIN1	ENSG00000134324	1.65274051	6.8519E-36
SEMA4G	ENSG00000095539	-2.089289088	7.6862E-36
UGT1A7	ENSG00000244122	-2.433904170	2.0197E-35
TSC22D3	ENSG00000157514	1.92165934	3.3466E-35

RTN4RL2	ENSG00000186907	-1.41592927(	3.362E-35
IL1RL1	ENSG00000115602	5.63819217	4.3699E-35
LRP5	ENSG00000162337	-1.44295176(	6.6034E-35
CORO2A	ENSG00000106789	-2.36776026(	9.7993E-35
PPM1D	ENSG00000170836	1.71048699	1.3565E-34
MMP15	ENSG00000102996	-1.93521449(	3.3207E-34
IGFBP4	ENSG00000141753	-1.34657827(	7.6945E-34
SPTSSA	ENSG00000165389	-1.47983983(	1.1794E-33
TTYH3	ENSG00000136295	-1.45484365(	1.5199E-33
ADAMTS9	ENSG00000163638	-1.78092423(	1.99E-33
PTPRU	ENSG00000060656	-1.75757129(	2.9798E-33
HEXIM1	ENSG00000186834	-1.40849071(	3.9322E-33
DNAJB9	ENSG00000128590	1.56981225	4.4079E-33
CSRNP1	ENSG00000144655	1.53399184	4.6926E-33
MYO18A	ENSG00000196535	-1.43224022(	5.0621E-33
OLFML2A	ENSG00000185585	-1.89991990(	1.0478E-32
ZNF274	ENSG00000171606	1.76872905	1.1339E-32
DIXDC1	ENSG00000150764	-2.51682369(	1.6239E-32
KIF12	ENSG00000136883	-3.34080671(	1.6703E-32
KLHL21	ENSG00000162413	1.58696916	1.9116E-32
CNTN1	ENSG00000018236	-1.51679934(	2.1364E-32
TNFAIP3	ENSG00000118503	1.44311459	4.89E-32
RASSF9	ENSG00000198774	-2.22705245(	4.911E-32
CDH17	ENSG00000079112	-1.63696681(	6.3491E-32
SLC40A1	ENSG00000138449	-2.19303561(	1.1223E-31
NAV3	ENSG00000067798	1.72915385	1.7154E-31
GTF2IP4	ENSG00000233369	-1.43027676(	1.9889E-31
ALDH5A1	ENSG00000112294	-2.62989199(	2.2739E-31
TMEM37	ENSG00000171227	-3.57622799(	2.7054E-31
SNHG12	ENSG00000197989	1.90068611	3.4653E-31
SMAD6	ENSG00000137834	-1.52826541(	3.8649E-31
SELENOS	ENSG00000131871	1.4802873	4.1908E-31
SMURF2	ENSG00000108854	1.52063457	5.3282E-31
TSC22D2	ENSG00000196428	1.89865368	5.9404E-31
KRT18	ENSG00000111057	-1.29232970(	6.721E-31
RNASE4	ENSG00000258818	-1.70897245(	1.0258E-30
PDE3A	ENSG00000172572	-2.46937584(	1.1919E-30
RELB	ENSG00000104856	1.49492124	1.5561E-30
FOSB	ENSG00000125740	3.31670172	1.8994E-30
PROS1	ENSG00000184500	-1.71121276(	2.2066E-30
RND3	ENSG00000115963	1.57942968	2.3504E-30
CD38	ENSG00000004468	-1.49574519(	4.3652E-30
FXYD2	ENSG00000137731	-1.31727623(	4.5784E-30
NTRK3	ENSG00000140538	-2.55860592(	4.7274E-30
EHF	ENSG00000135373	-2.17304430(	4.7967E-30
DTX4	ENSG00000110042	-3.01438890(	4.9221E-30
TNS3	ENSG00000136205	-1.29147287(	5.2775E-30
C11orf68	ENSG00000175573	1.6437674	5.4307E-30
PTGES	ENSG00000148344	-1.41436441(	5.4632E-30
PAPSS2	ENSG00000198682	-1.47672919(	6.5549E-30
ZNF554	ENSG00000172006	3.00188857	8.5592E-30
PIK3C2B	ENSG00000133056	-1.62295394(	8.781E-30
MARCH4	ENSG00000144583	1.55285275	1.0297E-29
FEM1C	ENSG00000145780	1.51984635	1.0824E-29
MAFG	ENSG00000197063	1.42563991	1.1016E-29
AKR1C1	ENSG00000187134	-1.25898909(	1.3095E-29
HIP1	ENSG00000127946	-1.41540630(	1.4141E-29
PLA2G4A	ENSG00000116711	-1.66227469(	1.6848E-29

CACNA1D	ENSG00000157388	-1.99335684	2.3739E-29
PLCH1	ENSG00000114805	-2.06334662	2.6002E-29
SERPINE1	ENSG00000106366	1.32689268	3.0114E-29
IGSF3	ENSG00000143061	-1.35824512	3.0894E-29
THSD7A	ENSG00000005108	-1.53228828	4.0215E-29
ELMO1	ENSG00000155849	-1.80645524	7.7084E-29
RAB3D	ENSG00000105514	-1.73909850	1.6151E-28
TUBB4A	ENSG00000104833	-2.27029474	1.639E-28
JMJD1C	ENSG00000171988	1.40029268	1.683E-28
ARRB1	ENSG00000137486	-2.22035598	1.9245E-28
TNS2	ENSG00000111077	-1.86751039	2.6062E-28
ALDH2	ENSG00000111275	-1.29815038	2.9294E-28
DHRS2	ENSG00000100867	2.04543548	3.0508E-28
MANF	ENSG00000145050	1.4270226	3.2109E-28
OAS2	ENSG00000111335	-2.10027851	3.3023E-28
USH1C	ENSG00000006611	-2.74371089	3.3756E-28
C10orf91	ENSG00000180066	-3.35961610	4.7307E-28
ASS1	ENSG00000130707	-1.51007548	4.7589E-28
GABRE	ENSG00000102287	-1.37797229	6.7691E-28
ADSSL1	ENSG00000185100	-2.62585834	1.0882E-27
PDLIM5	ENSG00000163110	-1.30777478	1.2071E-27
TMSB4X	ENSG00000205542	-1.20968852	1.2552E-27
PNKD	ENSG00000127838	-1.47825734	1.3109E-27
ARVCF	ENSG00000099889	2.04559058	1.4125E-27
AP5Z1	ENSG00000242802	1.50163642	2.5414E-27
AMPD3	ENSG00000133805	2.77671683	2.892E-27
AKR1C3	ENSG00000196139	-1.19946466	4.4596E-27
SLC23A1	ENSG00000170482	-1.94664637	5.6566E-27
MDM2	ENSG00000135679	1.32043499	5.8503E-27
TAF1D	ENSG00000166012	1.36262426	9.4888E-27
TSPYL2	ENSG00000184205	1.78013235	1.5678E-26
LGR4	ENSG00000205213	-1.20603027	1.6668E-26
SEMA6B	ENSG00000167680	-2.29156066	2.134E-26
KCNJ16	ENSG00000153822	-3.04983534	2.5655E-26
PON3	ENSG00000105852	-1.71369980	2.6288E-26
ASNS	ENSG00000070669	1.37180199	2.6582E-26
ULK1	ENSG00000177169	1.29084345	3.2132E-26
AKR1C2	ENSG00000151632	-1.17060872	4.0973E-26
SLC51B	ENSG00000186198	-3.11011009	7.1391E-26
AC022613.1	ENSG00000256802	-1.63975204	8.9772E-26
SMG1P2	ENSG00000205534	1.78630752	1.0325E-25
PBX1	ENSG00000185630	-1.40432737	1.1782E-25
FGFR3	ENSG00000068078	-2.45110665	1.3235E-25
DDIT4L	ENSG00000145358	-2.33644495	1.6975E-25
SULT2B1	ENSG00000088002	-2.96783863	1.9031E-25
MCM6	ENSG00000076003	-1.31638416	1.9324E-25
ANXA4	ENSG00000196975	-1.18741806	2.2613E-25
MYOCD	ENSG00000141052	-1.73861324	2.2695E-25
F5	ENSG00000198734	-1.81891210	2.4775E-25
RABGGTB	ENSG00000137955	1.35761583	2.5202E-25
SLC9A3R2	ENSG00000065054	-1.49825748	2.7908E-25
EREG	ENSG00000124882	1.22630052	2.8848E-25
CYR61	ENSG00000142871	1.44856182	2.9786E-25
EVA1C	ENSG00000166979	-1.88714559	3.1718E-25
DNAH17	ENSG00000187775	1.98507471	3.3038E-25
FAM83G	ENSG00000188522	1.28471719	3.355E-25
KIAA1147	ENSG00000257093	-1.34413089	4.039E-25
THAP1	ENSG00000131931	1.60882486	5.3213E-25

SLC44A1	ENSG00000070214	-1.21538767	5.4355E-25
SLPI	ENSG00000124107	-1.82261461	6.0653E-25
SOWAHC	ENSG00000198142	1.49206772	6.7479E-25
BTG2	ENSG00000159388	1.54092123	7.8607E-25
VPS37B	ENSG00000139722	1.39459762	9.0248E-25
PTGFRN	ENSG00000134247	-1.38114171	9.9344E-25
BTBD11	ENSG00000151136	-1.51108828	1.2548E-24
RASSF1	ENSG00000068028	1.5877057	1.402E-24
AC131160.1	ENSG00000283765	8.22684845	1.6781E-24
MTHFD2	ENSG00000065911	1.23193658	1.9882E-24
FLCN	ENSG00000154803	1.31199275	2.4281E-24
HLA-DMB	ENSG00000242574	-4.04403541	2.4834E-24
E2F2	ENSG00000007968	-2.25501973	2.5431E-24
RUNX1	ENSG00000159216	1.4027322	2.5828E-24
SH3PXD2A	ENSG00000107957	-1.50323172	2.9609E-24
RDH10	ENSG00000121039	-1.32016814	4.3965E-24
OCLN	ENSG00000197822	1.36353388	4.45E-24
GLP2R	ENSG00000065325	-1.49319819	5.3902E-24
C6orf48	ENSG00000204387	1.31790928	5.5059E-24
NUP210	ENSG00000132182	-1.19465115	7.1769E-24
NR4A1	ENSG00000123358	-1.75499080	7.738E-24
CXCL5	ENSG00000163735	-1.11615101	7.8515E-24
ZC3H12A	ENSG00000163874	-1.17304051	7.9209E-24
SDK2	ENSG00000069188	-2.13400939	7.9931E-24
PRAME	ENSG00000185686	-1.27141804	8.4766E-24
PCSK9	ENSG00000169174	-1.34117582	1.1809E-23
DAPK1	ENSG00000196730	-1.22115628	1.2251E-23
FNIP1	ENSG00000217128	1.3163131	1.2383E-23
HMGCS1	ENSG00000112972	1.78458496	1.2701E-23
STX3	ENSG00000166900	1.54975277	1.3708E-23
CA11	ENSG00000063180	-2.32106407	1.6156E-23
PCNA	ENSG00000132646	-1.15231123	2.3981E-23
WNT9A	ENSG00000143816	1.94709545	2.5227E-23
PTPN6	ENSG00000111679	-1.69978519	3.0526E-23
CA12	ENSG00000074410	-1.09526283	5.1293E-23
BICDL1	ENSG00000135127	-1.95143578	5.2061E-23
LIN7A	ENSG00000111052	-1.49367495	6.4418E-23
SFN	ENSG00000175793	1.18940601	7.3099E-23
ERGIC1	ENSG00000113719	-1.15205212	9.9436E-23
CTNNAL1	ENSG00000119326	1.27079005	1.0192E-22
SYNE1	ENSG00000131018	1.26810327	1.0206E-22
GPRIN3	ENSG00000185477	-1.14099426	1.195E-22
MAPK6	ENSG00000069956	1.18035067	1.2043E-22
IQSEC2	ENSG00000124313	-1.42950589	1.3234E-22
SEC24D	ENSG00000150961	1.28320761	1.3882E-22
ZBTB43	ENSG00000169155	1.96321488	1.4259E-22
SHROOM3	ENSG00000138771	-1.54872681	1.5111E-22
MSANTD3	ENSG00000066697	1.26933778	1.588E-22
GOLGA4	ENSG00000144674	1.24290201	1.6472E-22
ZNF555	ENSG00000186300	2.07209316	1.7455E-22
CHAC1	ENSG00000128965	2.32447976	1.8101E-22
ZMYM3	ENSG00000147130	-1.39699655	1.8785E-22
KCNJ6	ENSG00000157542	-2.52636077	2.1365E-22
RAB37	ENSG00000172794	-2.42447721	3.1737E-22
NOCT	ENSG00000151014	1.3506802	3.4684E-22
RNF145	ENSG00000145860	-1.18083324	4.1702E-22
PI4K2A	ENSG00000155252	1.30152691	4.5219E-22
CIDEC	ENSG00000187288	-2.98802194	4.9935E-22

MED10	ENSG00000133398	1.30626347	5.4891E-22
IGDCC4	ENSG00000103742	1.59683168	6.5337E-22
LY6K	ENSG00000160886	2.76330699	7.2168E-22
GRAMD1B	ENSG00000023171	-1.25582183	8.4103E-22
ZFAS1	ENSG00000177410	1.21014793	9.7537E-22
ADNP2	ENSG00000101544	1.24470518	9.9864E-22
TFE3	ENSG00000068323	1.18432183	1.0052E-21
C11orf86	ENSG00000173237	-1.51231034	1.0569E-21
TBC1D14	ENSG00000132405	-1.15431956	1.2133E-21
GAS5	ENSG00000234741	1.20114417	1.3087E-21
EPC1	ENSG00000120616	1.36439207	1.3272E-21
CLK1	ENSG00000013441	1.27286513	1.455E-21
TTC39B	ENSG00000155158	-1.49788895	1.46E-21
NRG1	ENSG00000157168	1.18356357	1.5838E-21
GFPT1	ENSG00000198380	1.13224784	1.9261E-21
RSRC2	ENSG00000111011	1.23868291	2.6392E-21
C1S	ENSG00000182326	-1.18744428	2.7941E-21
TPCN1	ENSG00000186815	-1.24056571	2.8108E-21
NNMT	ENSG00000166741	-1.99850039	3.4963E-21
PTPRS	ENSG00000105426	-1.25115277	4.1526E-21
PTK2B	ENSG00000120899	-1.25681219	4.199E-21
PHLDA1	ENSG00000139289	1.1339068	4.2927E-21
ADCY9	ENSG00000162104	-1.30075602	4.3386E-21
DNM1	ENSG00000106976	-1.39740096	4.4258E-21
SLC29A4	ENSG00000164638	-1.89287290	6.3081E-21
CBX5	ENSG00000094916	-1.08908915	6.42E-21
AL133352.1	ENSG00000255339	-2.82053614	1.0027E-20
OSER1	ENSG00000132823	1.28528872	1.0073E-20
GLS	ENSG00000115419	1.12781717	1.0615E-20
S100P	ENSG00000163993	-1.34845309	1.1235E-20
IFNL3	ENSG00000197110	-1.65636233	1.1966E-20
CXCL1	ENSG00000163739	-1.09003550	1.1996E-20
NPY4R	ENSG00000204174	-2.05515938	1.2097E-20
LRP1	ENSG00000123384	-1.13506088	1.5148E-20
GALNT4	ENSG00000257594	-2.33626573	1.5868E-20
FAM83A	ENSG00000147689	-2.44499688	1.7506E-20
EIF2AK3	ENSG00000172071	1.24771925	1.8081E-20
GALM	ENSG00000143891	-1.43298630	1.8427E-20
NPTX1	ENSG00000171246	1.47277058	2.1767E-20
COL4A4	ENSG00000081052	-1.43869725	2.2285E-20
CAMK2N1	ENSG00000162545	-1.30004606	2.2584E-20
IFNL2	ENSG00000183709	-1.54198488	2.5806E-20
TST	ENSG00000128311	-1.36093994	2.7234E-20
SUCNR1	ENSG00000198829	-2.22713022	2.8893E-20
CACNA1G	ENSG00000006283	-1.66530222	3.0393E-20
NEU1	ENSG00000204386	1.31408178	4.228E-20
LRIF1	ENSG00000121931	1.27608727	4.2528E-20
TNS1	ENSG00000079308	-1.56086546	4.918E-20
IL6R	ENSG00000160712	1.49145528	5.3823E-20
HJURP	ENSG00000123485	-1.11156964	5.8319E-20
KCTD5	ENSG00000167977	1.25154449	6.1089E-20
KIAA1191	ENSG00000122203	1.37364482	6.2904E-20
MGST3	ENSG00000143198	-1.09713114	6.919E-20
P2RY6	ENSG00000171631	-2.17317749	6.9742E-20
HBEGF	ENSG00000113070	2.0182392	8.3605E-20
MYRF	ENSG00000124920	-1.42191625	8.4804E-20
FLRT3	ENSG00000125848	-1.61384351	8.7133E-20
SNHG8	ENSG00000269893	1.35881222	8.7452E-20

CXCL8	ENSG00000169429	1.50964861	9.2688E-20
CSGALNACT2	ENSG00000169826	1.18640133	9.796E-20
CES1	ENSG00000198848	-1.60448028	1.0476E-19
TUFT1	ENSG00000143367	1.32368076	1.0729E-19
SCAF8	ENSG00000213079	1.20361092	1.0816E-19
KANK2	ENSG00000197256	-1.21965355	1.2059E-19
ZNF77	ENSG00000175691	2.1834837	1.2219E-19
GPRIN2	ENSG00000204175	-1.32375892	1.2842E-19
NFKB2	ENSG00000077150	1.08671012	1.4492E-19
LAMB1	ENSG00000091136	-1.03432000	1.6865E-19
ZNRF3	ENSG00000183579	-1.30425420	1.7033E-19
SRC	ENSG00000197122	-1.03388361	1.7268E-19
CCDC174	ENSG00000154781	1.39360788	1.7835E-19
UPP1	ENSG00000183696	1.1582168	2.0571E-19
INHBA	ENSG00000122641	2.86485713	2.1575E-19
ATF4	ENSG00000128272	1.07049919	2.2128E-19
EGFR	ENSG00000146648	1.05177475	2.2938E-19
CEBPG	ENSG00000153879	1.0878999	2.3442E-19
MGAM	ENSG00000257335	-2.11596630	2.383E-19
CREBRF	ENSG00000164463	1.50837209	2.6391E-19
CXCL2	ENSG00000081041	1.11247279	3.2299E-19
TMEM2	ENSG00000135048	-1.03428972	3.7037E-19
TGM2	ENSG00000198959	-1.00638255	4.1806E-19
ZNF654	ENSG00000175105	1.31303938	4.3828E-19
C1QTNF6	ENSG00000133466	-2.14173537	4.4194E-19
UFM1	ENSG00000120686	1.15594861	4.6677E-19
YOD1	ENSG00000180667	1.23283308	4.8291E-19
SCD5	ENSG00000145284	-2.02220350	5.1769E-19
LINC01137	ENSG00000233621	-1.43403720	5.3072E-19
SORL1	ENSG00000137642	-1.42731850	5.3997E-19
CCND3	ENSG00000112576	-1.62636373	5.601E-19
P3H4	ENSG00000141696	-1.29217369	5.6773E-19
TIMP2	ENSG00000035862	-1.03685155	7.3887E-19
HSPG2	ENSG00000142798	-1.02074834	7.4468E-19
LRP6	ENSG00000070018	-1.04892657	7.4557E-19
PC	ENSG00000173599	-1.16812582	7.8E-19
AL645608.1	ENSG00000223764	-3.36955431	8.2126E-19
BCHE	ENSG00000114200	-1.42649089	8.827E-19
PLD5	ENSG00000180287	-1.70337909	1.1814E-18
HSPB8	ENSG00000152137	1.36239502	1.2028E-18
SIRPA	ENSG00000198053	-1.10518937	1.2549E-18
HSP90B1	ENSG00000166598	1.02317914	1.2882E-18
CYP51A1	ENSG00000001630	1.08682822	1.4512E-18
EEA1	ENSG00000102189	1.24263937	1.4576E-18
TNFRSF10B	ENSG00000120889	1.03713341	1.487E-18
FAM84B	ENSG00000168672	-1.28412623	1.4888E-18
PLEK2	ENSG00000100558	1.6230143	1.5693E-18
PPFIBP2	ENSG00000166387	-1.49820317	1.5843E-18
DYRK1B	ENSG00000105204	1.2247644	1.6245E-18
CDON	ENSG00000064309	-1.80756759	1.7499E-18
PLCXD1	ENSG00000182378	-1.27036080	1.7561E-18
DNAJC22	ENSG00000178401	-1.41329773	1.8674E-18
PVT1	ENSG00000249859	1.23389354	1.9199E-18
GABPB1	ENSG00000104064	1.26649328	2.0027E-18
LHPP	ENSG00000107902	-1.94408364	2.1087E-18
GARS	ENSG00000106105	1.05920317	2.3014E-18
PLSCR4	ENSG00000114698	-1.89704330	2.4763E-18
ACSF2	ENSG00000167107	-1.89713682	2.5309E-18

EPS8L3	ENSG00000198758	-2.39884916	2.6007E-18
CASTOR3	ENSG00000239521	-1.55467687	2.7083E-18
APLP1	ENSG00000105290	-1.18377326	2.9074E-18
RALGAPA2	ENSG00000188559	-1.42176184	3.0131E-18
SOCS3	ENSG00000184557	1.12933146	3.0236E-18
LANCL1	ENSG00000115365	-1.05491276	3.1597E-18
TLK2	ENSG00000146872	1.13883959	3.3626E-18
GGT4P	ENSG00000280208	1.40307999	3.9043E-18
HNF1B	ENSG00000275410	-1.30148584	4.0326E-18
FLOT2	ENSG00000132589	-1.04367706	4.5798E-18
PTP4A3	ENSG00000184489	-1.75744532	4.7242E-18
PALM	ENSG00000099864	-1.76040523	5.1988E-18
LRP4	ENSG00000134569	-1.66033767	5.2846E-18
CYB5A	ENSG00000166347	-1.39249461	5.3806E-18
RNF6	ENSG00000127870	1.11662912	5.5154E-18
ABTB2	ENSG00000166016	1.09055399	5.8452E-18
SBDS	ENSG00000126524	1.09403206	6.2165E-18
ACSM3	ENSG00000005187	-1.97949193	6.4538E-18
METTL7B	ENSG00000170439	-2.11806162	7.6376E-18
SLC44A2	ENSG00000129353	-1.72125095	7.8578E-18
SIK1	ENSG00000142178	-1.02640168	9.2097E-18
AP003119.3	ENSG00000261578	-1.47349949	9.6112E-18
NEDD4	ENSG00000069869	1.01628984	9.6498E-18
LZTS2	ENSG00000107816	-1.18559901	9.7061E-18
ZNF79	ENSG00000196152	1.60285108	9.8778E-18
PON2	ENSG00000105854	-1.00051644	9.9598E-18
LRFN1	ENSG00000128011	-1.33035368	9.9824E-18
SCP2	ENSG00000116171	-1.15283019	1E-17
PIK3AP1	ENSG00000155629	-1.75397392	1.0219E-17
SPC24	ENSG00000161888	-1.73785872	1.0593E-17
ARC	ENSG00000198576	3.54053478	1.1369E-17
GLI1	ENSG00000111087	-1.65146350	1.223E-17
MSRB1	ENSG00000198736	-1.28299288	1.2309E-17
SDF2L1	ENSG00000128228	1.14494603	1.349E-17
HSPA13	ENSG00000155304	1.06670398	1.3611E-17
NOL4L	ENSG00000197183	-1.36618525	1.4826E-17
EIF1	ENSG00000173812	1.00351239	1.5037E-17
GPR20	ENSG00000204882	-3.25945104	1.5759E-17
GAL3ST1	ENSG00000128242	-1.48179020	1.7847E-17
AKAP17A	ENSG00000197976	1.10787123	1.881E-17
UCP2	ENSG00000175567	-1.23993060	2.1684E-17
ABCA2	ENSG00000107331	-1.08356073	2.2149E-17
SGSM2	ENSG00000141258	-1.20152002	2.2637E-17
ZNF574	ENSG00000105732	1.11796713	2.4363E-17
STK40	ENSG00000196182	1.31456606	2.8092E-17
AC073869.1	ENSG00000152117	1.23921443	2.8442E-17
EPC2	ENSG00000135999	1.26446685	3.4529E-17
SGK1	ENSG00000118515	-1.54529341	3.5543E-17
MSMO1	ENSG00000052802	1.1013432	3.681E-17
NFIL3	ENSG00000165030	1.13842469	3.7187E-17
SH3RF1	ENSG00000154447	1.11620636	3.8988E-17
HIST2H3C	ENSG00000203811	2.28263081	4.0191E-17
FAM13B	ENSG00000031003	1.19345061	4.1531E-17
ZSWIM4	ENSG00000132003	1.23772233	4.2048E-17
FOXRED2	ENSG00000100350	-1.23934407	4.3215E-17
S1PR3	ENSG00000213694	-1.08915004	5.029E-17
LINC02582	ENSG00000261780	-1.01802917	5.2925E-17
ZMYND8	ENSG00000101040	-1.11134469	5.5724E-17

BRD2	ENSG00000204256	1.0505219	6.1353E-17
FMN1	ENSG00000248905	-1.18607119	6.3007E-17
PAX7	ENSG00000009709	-1.29199925	6.6158E-17
GATA6	ENSG00000141448	1.64392494	7.3237E-17
SLC22A18	ENSG00000110628	-1.21060277	7.4944E-17
AC007325.2	ENSG00000277196	-3.48767672	7.6405E-17
SMG1P1	ENSG00000237296	1.13899692	7.9479E-17
SLC39A8	ENSG00000138821	-1.04075132	7.9836E-17
PCF11	ENSG00000165494	1.07992229	8.2833E-17
TNFRSF9	ENSG00000049249	1.53128929	8.3824E-17
TRAF1	ENSG00000056558	1.18659287	9.1739E-17
KLF7	ENSG00000118263	1.06591139	9.4193E-17
API5	ENSG00000166181	1.04337161	9.8013E-17
LURAP1L	ENSG00000153714	1.12587018	1.0006E-16
BCYRN1	ENSG00000236824	1.84581031	1.0031E-16
SCARA3	ENSG00000168077	-1.16457663	1.0379E-16
GOLGB1	ENSG00000173230	1.02238823	1.1368E-16
TMEM92	ENSG00000167105	-1.67222479	1.138E-16
ETS2	ENSG00000157557	1.03448345	1.1768E-16
AC004656.1	ENSG00000260822	-1.54256908	1.1853E-16
ADD3	ENSG00000148700	-2.05156809	1.206E-16
CTSF	ENSG00000174080	-1.49467675	1.2925E-16
SUCO	ENSG00000094975	1.14885756	1.3395E-16
CGN	ENSG00000143375	-1.85894650	1.5773E-16
SOX9	ENSG00000125398	-1.19358098	1.6613E-16
AC106886.5	ENSG00000282034	1.57562217	1.7217E-16
ING3	ENSG00000071243	1.588464	1.8542E-16
DNAJB11	ENSG00000090520	1.02468035	1.9217E-16
F2R	ENSG00000181104	-1.05775448	1.9625E-16
SUSD2	ENSG00000099994	-1.66400874	1.9747E-16
SYNGR1	ENSG00000100321	-1.92828176	2.1786E-16
MBD1	ENSG00000141644	1.17627156	2.2356E-16
BCL10	ENSG00000142867	1.18258953	2.3117E-16
CRY1	ENSG00000008405	1.14765966	2.5834E-16
TC2N	ENSG00000165929	-1.92521026	2.722E-16
SDHAP1	ENSG00000185485	1.26317304	2.8672E-16
GTF2IP1	ENSG00000277053	-1.02230994	2.9256E-16
MINPP1	ENSG00000107789	-1.10407745	3.2405E-16
LINC00973	ENSG00000240476	1.81151026	3.5219E-16
GABPB1-AS1	ENSG00000244879	-1.09607609	4.0068E-16
SENP3	ENSG00000161956	-1.03993252	4.0153E-16
FICD	ENSG00000198855	1.21833184	4.1068E-16
OXTR	ENSG00000180914	1.41013667	4.2366E-16
HHIPL2	ENSG00000143512	-1.15669320	4.6373E-16
TNC	ENSG00000041982	1.85526669	4.7084E-16
DNAJC25	ENSG00000059769	1.27880767	4.8578E-16
CACNA1H	ENSG00000196557	-1.82355315	4.8764E-16
TICAM1	ENSG00000127666	1.24016886	5.6906E-16
SMIM14	ENSG00000163683	-1.07230041	5.9014E-16
ASB13	ENSG00000196372	-1.31001933	6.4861E-16
PDK2	ENSG00000005882	-1.67231470	6.9431E-16
ABCD3	ENSG00000117528	-1.01756122	7.0374E-16
VAV3	ENSG00000134215	-1.47084937	7.6854E-16
MCM5	ENSG00000100297	-1.04888279	8.8913E-16
ELOVL3	ENSG00000119915	-3.35511982	1.0795E-15
RHNO1	ENSG00000171792	-1.21234115	1.1063E-15
ATP6V0D2	ENSG00000147614	5.20407859	1.1099E-15
IGFN1	ENSG00000163395	2.58123675	1.1649E-15

AL365205.1	ENSG00000124593	-2.28694417	1.1944E-15
PLD1	ENSG00000075651	-1.08519273	1.2116E-15
ADAMTS10	ENSG00000142303	-1.66453826	1.7065E-15
F7	ENSG00000057593	-3.52739474	1.7192E-15
OSGIN1	ENSG00000140961	1.06698474	1.9469E-15
LRRC8C	ENSG00000171488	1.1281521	2.0114E-15
ZNF134	ENSG00000213762	1.27165649	2.1337E-15
ZNF408	ENSG00000175213	1.28883193	2.1471E-15
NAV2	ENSG00000166833	1.34537614	2.3611E-15
FAM69B	ENSG00000165716	-1.29705113	2.3992E-15
ZMYM5	ENSG00000132950	1.34874691	2.6422E-15
FBXL19-AS1	ENSG00000260852	1.26094682	2.9907E-15
SMG1P5	ENSG00000183604	1.33624858	3.095E-15
GTPBP2	ENSG00000172432	1.03728863	3.4525E-15
ANG	ENSG00000214274	-1.67631279	3.5821E-15
CARMIL1	ENSG00000079691	-1.07737437	3.6998E-15
DDX10	ENSG00000178105	1.02752223	3.9315E-15
CEP135	ENSG00000174799	1.28887192	4.2519E-15
LBP	ENSG00000129988	-5.51675531	4.3502E-15
NPY4R2	ENSG00000264717	-1.84699155	4.6148E-15
ARID3B	ENSG00000179361	1.33710747	4.7705E-15
B4GAT1	ENSG00000174684	-1.27151512	4.9505E-15
WHAMM	ENSG00000156232	1.11748221	5.1497E-15
TMSB4XP8	ENSG00000187653	-1.33460329	5.1747E-15
TESC	ENSG00000088992	-1.36077042	5.5402E-15
ZFYVE1	ENSG00000165861	1.12387699	5.6649E-15
RNF19B	ENSG00000116514	1.05803519	6.0842E-15
ZNF185	ENSG00000147394	1.46055804	6.1516E-15
LZTFL1	ENSG00000163818	1.31135768	6.4445E-15
SPHK1	ENSG00000176170	1.23589197	7.4757E-15
HMGCR	ENSG00000113161	1.04333348	8.214E-15
SNHG1	ENSG00000255717	1.05682398	9.1702E-15
NTHL1	ENSG00000065057	-1.35865033	9.1768E-15
ULBP1	ENSG00000111981	1.89323374	1.0588E-14
BMP6	ENSG00000153162	-1.13803935	1.2578E-14
EFNA5	ENSG00000184349	-1.00358728	1.2614E-14
HMGA2	ENSG00000149948	1.02996786	1.3222E-14
SLC16A3	ENSG00000141526	-1.35347565	1.3819E-14
CDH4	ENSG00000179242	1.70208321	1.3868E-14
CBS	ENSG00000160200	1.3380764	1.4502E-14
NR4A2	ENSG00000153234	-1.30849029	1.4613E-14
ZSCAN12P1	ENSG00000219891	4.42333076	1.4838E-14
SELENBP1	ENSG00000143416	-3.16788857	1.5246E-14
NOSTRIN	ENSG00000163072	-1.28524244	1.5276E-14
NLGN2	ENSG00000169992	-1.21420923	1.5788E-14
LYSMD3	ENSG00000176018	1.03005968	1.5833E-14
BCAT1	ENSG00000060982	1.27087278	1.5849E-14
CCDC9	ENSG00000105321	1.07260807	1.5992E-14
TRIP11	ENSG00000100815	1.0038741	1.778E-14
N4BP3	ENSG00000145911	1.49051243	1.8048E-14
IKZF5	ENSG00000095574	1.13068738	1.9114E-14
PPP4R4	ENSG00000119698	2.08747478	1.9283E-14
TIPARP	ENSG00000163659	2.62168004	1.9574E-14
SEMA7A	ENSG00000138623	2.15851388	2.1227E-14
ST3GAL2	ENSG00000157350	-1.01550815	2.1236E-14
CHIC2	ENSG00000109220	1.47020646	2.2838E-14
HCN2	ENSG00000099822	-1.24783098	2.5198E-14
PIM1	ENSG00000137193	1.02337877	2.6728E-14

FGL1	ENSG00000104760	-1.76681018	2.7789E-14
DHRS3	ENSG00000162496	-1.65291632	2.8134E-14
REV3L	ENSG00000009413	1.00191616	2.9338E-14
XYLT2	ENSG00000015532	-1.03266565	3.0083E-14
TP53INP1	ENSG00000164938	1.22516365	3.2438E-14
MYO1A	ENSG00000166866	-3.40305378	3.3855E-14
HIST2H3A	ENSG00000203852	2.31774016	3.4278E-14
UPF3B	ENSG00000125351	1.08793233	3.5051E-14
MARCKSL1	ENSG00000175130	-1.01948346	3.6142E-14
RIMS3	ENSG00000117016	-1.74860057	3.6302E-14
HSPA14	ENSG00000284024	1.06584438	3.772E-14
FAM20C	ENSG00000177706	-1.20370614	3.9538E-14
ATP8B1	ENSG00000081923	-1.13585529	4.0526E-14
HHIP	ENSG00000164161	-2.42389096	4.5102E-14
UGT1A1	ENSG00000241635	-3.50527579	4.5536E-14
FHOD1	ENSG00000135723	-1.08734645	4.6414E-14
AC233724.15	ENSG00000283740	-3.30262686	4.7129E-14
LPCAT3	ENSG00000111684	-1.03584509	5.1157E-14
PKDCC	ENSG00000162878	-1.23122985	6.6858E-14
CARHSP1	ENSG00000153048	-1.07865549	7.4153E-14
ABCC9	ENSG00000069431	2.31957053	7.4525E-14
NDRG2	ENSG00000165795	-2.71643483	7.5151E-14
ZKSCAN5	ENSG00000196652	1.09841998	7.5813E-14
TMEM268	ENSG00000157693	1.27838889	7.9413E-14
ZNF436	ENSG00000125945	1.14454642	8.9545E-14
DAAM1	ENSG00000100592	1.21218535	9.0744E-14
MED15	ENSG00000099917	1.00294095	9.834E-14
PTHLH	ENSG00000087494	1.7568422	9.9985E-14
PMS2CL	ENSG00000187953	1.22287152	1.0192E-13
ZNF343	ENSG00000088876	1.21206851	1.0871E-13
SYT12	ENSG00000173227	-2.33196335	1.14E-13
HNMT	ENSG00000150540	-2.06498342	1.1984E-13
RBM20	ENSG00000203867	-1.47521887	1.2749E-13
TLE2	ENSG00000065717	-2.22355674	1.3712E-13
DCLK1	ENSG00000133083	1.77651996	1.5392E-13
STAG3	ENSG00000066923	-1.69886331	1.6384E-13
STARD4	ENSG00000164211	1.20696755	1.7339E-13
ST8SIA4	ENSG00000113532	-2.04146891	1.7994E-13
ADRA2C	ENSG00000184160	1.46448816	1.8454E-13
SERPINB8	ENSG00000166401	1.31142317	1.8851E-13
ITM2C	ENSG00000135916	-1.03969290	2.1062E-13
PASK	ENSG00000115687	-1.36195587	2.1459E-13
CISH	ENSG00000114737	-2.99618766	2.2428E-13
ENTPD7	ENSG00000198018	1.22728639	2.4297E-13
PDE7A	ENSG00000205268	-1.06305206	2.5745E-13
BRPF1	ENSG00000156983	1.04299932	2.5973E-13
BTBD10	ENSG00000148925	1.02415131	2.7079E-13
EDA2R	ENSG00000131080	1.02714362	2.8923E-13
LARP1B	ENSG00000138709	1.08563443	2.9438E-13
ALDH1L2	ENSG00000136010	1.08762889	3.1016E-13
LINC00173	ENSG00000196668	-Inf	3.1115E-13
EXPH5	ENSG00000110723	1.55391101	3.1892E-13
AL390719.1	ENSG00000217801	1.80977	3.5934E-13
SPC25	ENSG00000152253	-1.32071543	3.731E-13
BCAS1	ENSG00000064787	-2.56940843	4.1505E-13
MT-RNR1	ENSG00000211459	1.29346192	4.1734E-13
SYT17	ENSG00000103528	-1.22973917	4.3479E-13
KCTD17	ENSG00000100379	-1.20015571	4.794E-13

AC010616.1	ENSG00000268041	4.26972997	4.8008E-13
CSNK1G1	ENSG00000169118	1.0309524	5.1474E-13
ZNF467	ENSG00000181444	-3.287589457	5.2951E-13
RRN3P1	ENSG00000248124	1.45406809	5.4732E-13
RAB20	ENSG00000139832	-2.470633865	5.509E-13
AP001972.5	ENSG00000279117	-2.079366550	5.5627E-13
SP2	ENSG00000167182	1.59506013	5.6042E-13
RNA5-8SN4	ENSG00000275215	2.2868757	6.1484E-13
MBD6	ENSG00000166987	1.0465171	6.1872E-13
PLEKHM1	ENSG00000225190	1.03237876	6.7475E-13
DGCR6	ENSG00000183628	-1.820314990	6.9735E-13
ZSCAN25	ENSG00000197037	1.21020359	7.5368E-13
HS3ST1	ENSG00000002587	-2.462701680	7.8524E-13
SNTB1	ENSG00000172164	-1.559368794	8.1403E-13
PDRG1	ENSG00000088356	1.06522045	8.6755E-13
SERPINI1	ENSG00000163536	-2.466116855	8.945E-13
HNF1A-AS1	ENSG00000241388	-3.183461830	9.0813E-13
SLC17A3	ENSG00000124564	-2.008198041	9.2666E-13
BBC3	ENSG00000105327	1.30016469	9.547E-13
MAGEE1	ENSG00000198934	-1.842553672	1.0197E-12
PCGF1	ENSG00000115289	1.12570665	1.0264E-12
PEAR1	ENSG00000187800	2.29304399	1.1012E-12
ABHD14B	ENSG00000114779	-1.288043267	1.1442E-12
GPC6	ENSG00000183098	-1.080081709	1.1812E-12
FBLN1	ENSG00000077942	-1.078529924	1.2007E-12
SEMA4D	ENSG00000187764	-1.202680006	1.2176E-12
CORO2B	ENSG00000103647	3.59267045	1.3277E-12
BEX2	ENSG00000133134	1.68053946	1.3664E-12
COL6A2	ENSG00000142173	1.13507369	1.3955E-12
AQP3	ENSG00000165272	-1.541193885	1.4431E-12
SPATA2	ENSG00000158480	1.13131291	1.4764E-12
EPHX1	ENSG00000143819	-1.074978947	1.5102E-12
RHPN1	ENSG00000158106	-1.418309134	1.5355E-12
PLLP	ENSG00000102934	-1.460905109	1.5494E-12
ZNF777	ENSG00000196453	1.02638231	1.5553E-12
GPR37	ENSG00000170775	-2.051075660	1.5781E-12
PLXND1	ENSG00000004399	-1.565759891	1.7637E-12
HS6ST1	ENSG00000136720	-1.016901280	1.7745E-12
PPARGC1B	ENSG00000155846	-1.740679070	1.7917E-12
SLC6A9	ENSG00000196517	1.22750737	1.8163E-12
APOH	ENSG00000091583	-1.966044956	1.9826E-12
SLC45A1	ENSG00000162426	-1.480484449	1.9983E-12
CRELD2	ENSG00000184164	1.09631079	2.227E-12
CCNL1	ENSG00000163660	1.57536675	2.3062E-12
LPCAT4	ENSG00000176454	-1.010628151	2.3822E-12
LRRK2	ENSG00000188906	-1.004275741	2.5319E-12
FJX1	ENSG00000179431	1.0118645	2.6356E-12
GSDMD	ENSG00000104518	-1.182627535	2.7359E-12
LYST	ENSG00000143669	1.09013744	2.8001E-12
EMP2	ENSG00000213853	-1.003502169	2.9659E-12
C3orf38	ENSG00000179021	1.04594721	3.0423E-12
BCL2L11	ENSG00000153094	-1.989944060	3.1468E-12
ACACB	ENSG00000076555	-1.149078590	3.1702E-12
FGG	ENSG00000171557	-3.174807185	3.3569E-12
NKX3-1	ENSG00000167034	1.30243514	3.4394E-12
LRRC20	ENSG00000172731	-1.137772425	3.5333E-12
SEC14L4	ENSG00000133488	-1.321997246	3.636E-12
PDGFRL	ENSG00000104213	-1.913877231	3.7837E-12

TBC1D22B	ENSG00000065491	1.11592511	3.8764E-12
CTGF	ENSG00000118523	1.26246987	3.8833E-12
RBM48	ENSG00000127993	1.21310269	3.9847E-12
THAP2	ENSG00000173451	1.58156679	4.1244E-12
CROCCP2	ENSG00000215908	1.03565891	4.1542E-12
ZNF165	ENSG00000197279	2.76947024	4.3476E-12
FOXQ1	ENSG00000164379	-1.12107695	4.3792E-12
MED26	ENSG00000105085	1.29078167	4.4551E-12
SLC27A2	ENSG00000140284	-1.46618723	4.5986E-12
THEM6	ENSG00000130193	-1.04719087	4.6571E-12
DAGLB	ENSG00000164535	1.05361014	4.6735E-12
AC141586.1	ENSG00000215154	1.38281899	4.8198E-12
FBLN5	ENSG00000140092	-2.59229689	4.9835E-12
ARHGEF26	ENSG00000114790	-1.63871152	5.4636E-12
HRH2	ENSG00000113749	-3.02198294	5.7083E-12
SH2B2	ENSG00000160999	-2.22160387	5.7654E-12
ING1	ENSG00000153487	1.27655864	5.8429E-12
NECTIN1	ENSG00000110400	-1.25425001	5.9907E-12
ZNF329	ENSG00000181894	2.28627764	5.9971E-12
ARHGEF39	ENSG00000137135	-1.35928426	6.1958E-12
CCDC106	ENSG00000173581	-1.45020865	6.3327E-12
AP000757.2	ENSG00000254844	-2.86930954	6.6958E-12
FKBP11	ENSG00000134285	1.06232598	6.6975E-12
ZNF26	ENSG00000198393	1.2013301	7.1562E-12
JAKMIP3	ENSG00000188385	-1.07866927	7.89E-12
ADM2	ENSG00000128165	1.49414981	7.9244E-12
SLITRK5	ENSG00000165300	-1.56202872	7.9521E-12
CYLD	ENSG00000083799	1.27079906	7.9783E-12
DDC	ENSG00000132437	-1.16013849	1.0702E-11
SMAD7	ENSG00000101665	1.04225259	1.0947E-11
SIRT1	ENSG00000096717	1.06101375	1.2013E-11
CGB8	ENSG00000213030	3.83302757	1.2478E-11
NFKBIB	ENSG00000104825	1.1697578	1.2809E-11
CPOX	ENSG00000080819	-1.00427243	1.3819E-11
ABCA7	ENSG00000064687	-1.02858056	1.4327E-11
ADGRL1	ENSG00000072071	-1.51440065	1.4982E-11
CHST1	ENSG00000175264	3.28775535	1.6197E-11
RBFOX3	ENSG00000167281	-1.99207397	1.6327E-11
HILPDA	ENSG00000135245	-1.14515050	1.7028E-11
TRAF6	ENSG00000175104	1.07482961	1.8168E-11
KAT14	ENSG00000149474	-1.17466078	1.8956E-11
DOCK8	ENSG00000107099	-1.73759005	1.9077E-11
RHBDD3	ENSG00000100263	1.01112541	1.9361E-11
TJAP1	ENSG00000137221	1.00756666	1.9997E-11
GAS2L3	ENSG00000139354	1.12470702	2.031E-11
PPARGC1A	ENSG00000109819	-1.81868447	2.1579E-11
UNC93B1	ENSG00000110057	-1.20201195	2.2113E-11
THAP9-AS1	ENSG00000251022	1.14974586	2.38E-11
BDH2	ENSG00000164039	-2.01460143	2.522E-11
IL11	ENSG00000095752	1.10308713	2.5221E-11
PECR	ENSG00000115425	-1.00966282	2.7902E-11
GSTM4	ENSG00000168765	-2.19636873	2.7969E-11
CDCA7	ENSG00000144354	-1.00038882	2.8048E-11
ENO1P1	ENSG00000244457	-2.95006904	2.9971E-11
RIOK3	ENSG00000101782	1.15278013	3.0326E-11
UPK3B	ENSG00000243566	-1.27849887	3.1488E-11
AC100803.2	ENSG00000261655	-4.06261592	3.16E-11
GYG2	ENSG00000056998	-1.96319044	3.3634E-11

YRDC	ENSG00000196449	1.02822305	3.421E-11
LMTK3	ENSG00000142235	-1.511916562	3.6267E-11
ZNF516	ENSG00000101493	-1.337213904	3.689E-11
HAUS3	ENSG00000214367	1.10054087	3.8276E-11
FBXW7	ENSG00000109670	1.11525089	3.9084E-11
RARRES3	ENSG00000133321	-1.479218485	4.123E-11
PDK3	ENSG00000067992	-1.241935032	4.4651E-11
ZNF618	ENSG00000157657	-1.274798448	4.6615E-11
RGS3	ENSG00000138835	-1.156387341	4.8607E-11
RNF157	ENSG00000141576	-1.251807784	5.3535E-11
REL	ENSG00000162924	1.00189922	5.613E-11
AC239868.1	ENSG00000261716	1.09533182	6.1589E-11
OPHN1	ENSG00000079482	-1.173554870	6.1623E-11
AC008560.1	ENSG00000253251	1.72168015	6.4816E-11
C5AR1	ENSG00000197405	-2.328011469	6.5267E-11
CLDN15	ENSG00000106404	1.16705763	6.6854E-11
CHST12	ENSG00000136213	-1.103239247	7.0004E-11
ZNF267	ENSG00000185947	1.02320148	7.3231E-11
KREMEN1	ENSG00000183762	-1.167711012	8.0136E-11
MMP9	ENSG00000100985	5.50729572	8.9578E-11
SPOCK1	ENSG00000152377	1.40543645	9.5746E-11
DNAJC27	ENSG00000115137	1.63044518	1.1012E-10
KCTD7	ENSG00000243335	-1.303252771	1.1221E-10
PLA2R1	ENSG00000153246	-1.499087701	1.3582E-10
ADAM23	ENSG00000114948	-1.054129222	1.3841E-10
BLOC1S1	ENSG00000135441	-1.216975164	1.4743E-10
HAUS4	ENSG00000092036	-1.031540582	1.541E-10
RGS20	ENSG00000147509	1.30975252	1.6174E-10
ADGRB2	ENSG00000121753	-1.090756003	1.6761E-10
RNF43	ENSG00000108375	-1.776702451	1.7238E-10
BIRC7	ENSG00000101197	-1.786457198	1.7872E-10
ITGB6	ENSG00000115221	-1.465317747	1.803E-10
PDPK2P	ENSG00000205918	1.38959126	1.8906E-10
AC016831.7	ENSG00000285106	-1.685416585	2.0553E-10
ZNF703	ENSG00000183779	-1.702773396	2.0771E-10
AP000439.2	ENSG00000255774	-1.447483183	2.2181E-10
CCT6P3	ENSG00000234585	1.41601215	2.4049E-10
PCDHAC2	ENSG00000243232	-1.271196797	2.5189E-10
VSIG10L	ENSG00000186806	-1.287032253	2.5817E-10
MOK	ENSG00000080823	1.12462513	2.6988E-10
THRB	ENSG00000151090	-1.104204492	2.7344E-10
IL1R2	ENSG00000115590	-1.436172719	2.8079E-10
HSD17B7	ENSG00000132196	1.15525096	2.8701E-10
JMJD4	ENSG00000081692	-1.057532223	2.9348E-10
ATXN7	ENSG00000163635	1.10076587	3.1214E-10
TNFRSF1A	ENSG00000067182	-1.193000046	3.1417E-10
PCYOX1L	ENSG00000145882	-1.994973542	3.5195E-10
HADH	ENSG00000138796	-1.078280887	4.4636E-10
CD163L1	ENSG00000177675	2.56716765	5.0834E-10
HIST2H2BE	ENSG00000184678	1.06647058	5.6707E-10
HS3ST6	ENSG00000162040	-1.486643662	5.8736E-10
STAT4	ENSG00000138378	-1.503996618	5.9041E-10
CACNG6	ENSG00000130433	-1.185841310	5.9766E-10
SLC22A1	ENSG00000175003	3.76676609	6.0787E-10
ANO5	ENSG00000171714	-1.006040300	6.6491E-10
CASTOR2	ENSG00000274070	-1.814958007	6.7658E-10
ORC1	ENSG00000085840	-1.043929116	7.1375E-10
NAGA	ENSG00000198951	-1.067001216	7.6154E-10

SGK3	ENSG00000104205	1.15856274	7.8909E-10
DNMT3A	ENSG00000119772	-1.08294666	8.0041E-10
WBP4	ENSG00000120688	1.04062582	8.1047E-10
SH3BGRL2	ENSG00000198478	-1.35959508	8.6513E-10
NFKBIL1	ENSG00000204498	1.0557623	8.9652E-10
CENPS	ENSG00000175279	-1.18180139	9.6482E-10
WNK2	ENSG00000165238	-1.21811662	9.875E-10
GALNT9	ENSG00000182870	2.40618201	1.0145E-09
AP002373.1	ENSG00000255663	6.22426151	1.0621E-09
KRT7-AS	ENSG00000257671	-1.04021137	1.1241E-09
BX890604.1	ENSG00000205664	1.28633666	1.1385E-09
MAPK8IP1	ENSG00000121653	1.39233816	1.1441E-09
PISD	ENSG00000241878	1.02427691	1.1618E-09
HMGN3	ENSG00000118418	-1.02666335	1.258E-09
MIR29B2CHG	ENSG00000203709	-1.33733419	1.2897E-09
CCR7	ENSG00000126353	-2.03043994	1.4426E-09
SYT6	ENSG00000134207	-1.21752916	1.4676E-09
NFXL1	ENSG00000170448	1.00653767	1.4774E-09
CENPL	ENSG00000120334	1.13139714	1.5371E-09
GUCY2EP	ENSG00000204529	-5.72151529	1.5378E-09
MAP3K8	ENSG00000107968	-1.03872307	1.5401E-09
TMEM102	ENSG00000181284	-1.17002774	1.6575E-09
TMTC2	ENSG00000179104	-1.26266649	1.7658E-09
CCDC186	ENSG00000165813	1.12645166	1.8539E-09
RHCG	ENSG00000140519	1.28133271	1.8744E-09
EPHX2	ENSG00000120915	-1.37150078	1.878E-09
MGST2	ENSG00000085871	-1.23281852	1.9105E-09
ZNF704	ENSG00000164684	-1.03319191	1.9432E-09
DUSP10	ENSG00000143507	1.21546092	2.0278E-09
AC243772.2	ENSG00000233030	3.90313453	2.0848E-09
THBD	ENSG00000178726	1.0761744	2.1108E-09
HIST1H2BN	ENSG00000233822	1.66089852	2.1761E-09
CEBPD	ENSG00000221869	-1.02358752	2.195E-09
BRF2	ENSG00000104221	1.08168817	2.2193E-09
AC110079.1	ENSG00000260404	1.04603741	2.2207E-09
FAM46B	ENSG00000158246	2.02637681	2.3903E-09
LINC01106	ENSG00000175772	-1.35610526	2.5016E-09
CREB5	ENSG00000146592	1.09449253	2.5711E-09
MPP2	ENSG00000108852	-1.89122451	2.6664E-09
VWA1	ENSG00000179403	-1.83877917	2.8887E-09
TPD52L1	ENSG00000111907	-1.03468022	2.9013E-09
ZFP14	ENSG00000142065	-1.20946808	2.9353E-09
MAP3K14-AS	ENSG00000267278	-1.59378643	2.9978E-09
SESN1	ENSG00000080546	1.13700289	3.1641E-09
NEXN	ENSG00000162614	2.31990454	3.2015E-09
SSBP4	ENSG00000130511	-1.00512609	3.3656E-09
FER1L4	ENSG00000088340	-1.45311647	3.6997E-09
EXOC3L4	ENSG00000205436	-2.25136929	4.0274E-09
SH2D5	ENSG00000189410	1.88757364	4.1343E-09
HUNK	ENSG00000142149	-1.59843520	4.5835E-09
IBA57	ENSG00000181873	1.25758559	4.5987E-09
AC092118.1	ENSG00000187185	-1.28731611	4.7617E-09
NCF2	ENSG00000116701	1.05078282	5.0585E-09
C19orf66	ENSG00000130813	-1.07016830	5.1896E-09
NKX2-5	ENSG00000183072	1.38888145	5.3109E-09
ANO1	ENSG00000131620	1.90438372	5.4394E-09
BDNF	ENSG00000176697	-1.43111506	6.0366E-09
AL391427.1	ENSG00000224251	-1.87480812	6.4605E-09

SRPX2	ENSG00000102359	-1.32296202(	7.4874E-09
POLK	ENSG00000122008	1.04577827	7.7486E-09
AC007191.1	ENSG00000279407	-1.59045435;	8.273E-09
RF00096	ENSG00000238840	-1.68065209!	8.3652E-09
DGKE	ENSG00000153933	1.21058209	8.7219E-09
SLC46A3	ENSG00000139508	-1.28326935(	8.7551E-09
GACAT2	ENSG00000265962	-1.68314786(	9.57E-09
OSBPL5	ENSG00000021762	-1.18318950؛	9.7173E-09
AC027290.2	ENSG00000280138	1.51189816	9.7512E-09
PPT2	ENSG00000221988	-1.21067230:	9.7699E-09
ADRA1D	ENSG00000171873	-1.85029233؛	1.0078E-08
AGFG2	ENSG00000106351	-1.12773233:	1.0251E-08
ATP7B	ENSG00000123191	-1.15333605:	1.0267E-08
DUSP7	ENSG00000164086	1.0846991	1.0697E-08
ZNF317	ENSG00000130803	1.28573198	1.0827E-08
DHODH	ENSG00000102967	-1.05059956(	1.0874E-08
CLIP4	ENSG00000115295	1.03285316	1.1153E-08
ITPKA	ENSG00000137825	2.16537479	1.1343E-08
RNA5-8SN3	ENSG00000278233	2.28345982	1.2835E-08
AC044849.1	ENSG00000272256	3.60600008	1.3362E-08
RPLP0P2	ENSG00000243742	2.52448003	1.4158E-08
ENTPD2	ENSG00000054179	-3.04847367(	1.4342E-08
IER5L	ENSG00000188483	-1.40175434:	1.4404E-08
INSL4	ENSG00000120211	-1.20145879!	1.4592E-08
AC095055.1	ENSG00000270681	3.0599335	1.5447E-08
SCN1B	ENSG00000105711	-1.18584714(	1.5523E-08
ZNF841	ENSG00000197608	1.35631721	1.5631E-08
HIST2H2AA3	ENSG00000203812	1.07521104	1.5996E-08
MEG3	ENSG00000214548	1.72285877	1.6016E-08
CYP4F12	ENSG00000186204	-1.55162614:	1.6113E-08
MAP6D1	ENSG00000180834	-3.75899913;	1.6155E-08
IFRD1	ENSG00000006652	1.45100643	1.6197E-08
HIST2H2AA4	ENSG00000272196	1.070244	1.675E-08
AC125421.1	ENSG00000263574	Inf	1.7248E-08
ARL14	ENSG00000179674	2.02036431	1.7517E-08
LRRN2	ENSG00000170382	-1.09126236(	1.8158E-08
GMDS	ENSG00000112699	-1.21586103(	1.8568E-08
MYO7A	ENSG00000137474	1.35931258	1.9015E-08
ZSCAN31	ENSG00000235109	1.41483536	1.9345E-08
LCN2	ENSG00000148346	1.20094329	2.022E-08
CFAP69	ENSG00000105792	1.51638829	2.0882E-08
AC027601.1	ENSG00000260005	1.70329068	2.1111E-08
C9orf152	ENSG00000188959	-2.99655985:	2.1487E-08
BAIAP2-AS1	ENSG00000226137	-1.21067417;	2.1511E-08
VIL1	ENSG00000127831	-2.35244494;	2.329E-08
ST6GAL1	ENSG00000073849	-1.19823198;	2.4079E-08
SEMA3A	ENSG00000075213	-1.06983757(	2.6031E-08
VTN	ENSG00000109072	-1.30066713!	2.7226E-08
KCNMA1	ENSG00000156113	1.34579532	2.8074E-08
TGFBR3	ENSG00000069702	-1.03776378(	2.8886E-08
LINC02532	ENSG00000235142	-2.18107238؛	2.9153E-08
AC007325.4	ENSG00000278817	-1.61004603!	3.1496E-08
AC023157.3	ENSG00000276900	1.74935729	3.2101E-08
SCARA5	ENSG00000168079	-2.52016547(	3.2926E-08
PCDH1	ENSG00000156453	1.37638871	3.3E-08
RAB39B	ENSG00000155961	1.12989009	3.4314E-08
RRAS2	ENSG00000133818	1.0145585	3.8779E-08
SLC19A3	ENSG00000135917	-1.12533544:	4.0422E-08

ETV1	ENSG00000006468	-1.17242223	4.1033E-08
ZNF211	ENSG00000121417	1.11579967	4.1743E-08
MIR22HG	ENSG00000186594	1.47526516	4.4698E-08
IGFLR1	ENSG00000126246	-2.11795912	4.7906E-08
GRB7	ENSG00000141738	-1.10718538	4.8545E-08
UNC13A	ENSG00000130477	-1.07990294	4.8994E-08
KRT15	ENSG00000171346	1.94802138	4.989E-08
KRT8	ENSG00000170421	-1.17797732	5.1031E-08
NOVA1	ENSG00000139910	-1.13993313	5.4184E-08
ZNF484	ENSG00000127081	1.06891378	5.6097E-08
KAZN	ENSG00000189337	-1.55244442	5.8019E-08
HRK	ENSG00000135116	2.23106141	5.8373E-08
FMO5	ENSG00000131781	-2.17673514	6.2303E-08
RNA5-8S4	ENSG00000276700	2.31506985	6.3188E-08
LINC01006	ENSG00000182648	1.36951869	6.5212E-08
RNA5-8SN1	ENSG00000275757	2.34266059	6.6863E-08
AC083899.2	ENSG00000233671	4.63157547	7.2093E-08
AKR1C4	ENSG00000198610	-2.47335463	7.3672E-08
GRB14	ENSG00000115290	-1.27478940	7.3836E-08
SAMD4A	ENSG00000020577	1.2475251	7.427E-08
CYP2S1	ENSG00000167600	-1.08446943	7.5367E-08
SMG1P3	ENSG00000180747	1.43554938	9.0294E-08
POLA2	ENSG00000014138	-1.00728959	9.7075E-08
ITIH2	ENSG00000151655	-2.08032907	9.8143E-08
AC124067.4	ENSG00000254290	-3.88212633	9.9623E-08
HIST1H1E	ENSG00000168298	2.7255007	1.0444E-07
SHISAL1	ENSG00000138944	1.27240581	1.0882E-07
MAST3	ENSG00000099308	-1.25768710	1.1013E-07
LHX2	ENSG00000106689	-1.66994199	1.1504E-07
RRAD	ENSG00000166592	2.26748741	1.1537E-07
PHLDA3	ENSG00000174307	1.3776078	1.1647E-07
STK19	ENSG00000204344	1.0776268	1.1673E-07
AL033397.1	ENSG00000231683	-1.13113601	1.1983E-07
DOC2B	ENSG00000272636	-1.83624737	1.1997E-07
NRGN	ENSG00000154146	-2.70861615	1.2908E-07
SIPA1	ENSG00000213445	-1.14759266	1.3016E-07
WIPI1	ENSG00000070540	1.00193836	1.3367E-07
PRG4	ENSG00000116690	-1.70347697	1.369E-07
AL451069.3	ENSG00000234311	-2.93188553	1.3873E-07
FAM24B	ENSG00000213185	1.17116204	1.4041E-07
AC108751.5	ENSG00000244503	-3.13864050	1.4477E-07
HHIP-AS1	ENSG00000248890	-2.43871569	1.4504E-07
TFCP2L1	ENSG00000115112	-1.01351989	1.4856E-07
RBP4	ENSG00000138207	-1.93956918	1.5004E-07
SP6	ENSG00000189120	1.43825763	1.5137E-07
CCDC96	ENSG00000173013	1.98408429	1.6188E-07
ZNF669	ENSG00000188295	1.69519594	1.6311E-07
AOX1	ENSG00000138356	1.10206254	1.6845E-07
MGAT5	ENSG00000152127	-1.59990500	1.7244E-07
RRAGC	ENSG00000116954	1.09593568	1.7396E-07
CD302	ENSG00000241399	-2.03494775	1.8143E-07
RHBDL3	ENSG00000141314	-1.99256736	1.8755E-07
AC097359.2	ENSG00000270194	1.46094984	1.9164E-07
FAM19A5	ENSG00000219438	-1.33685765	1.9363E-07
MAPT	ENSG00000186868	-1.89678660	2.0029E-07
CRYAB	ENSG00000109846	-3.48682466	2.0834E-07
ENPP1	ENSG00000197594	-1.07147929	2.1282E-07
RHOU	ENSG00000116574	-1.00084867	2.1318E-07

ASIC1	ENSG00000110881	-1.52129566	2.1357E-07
ABCC6	ENSG00000091262	-2.09001725	2.2486E-07
ZNF383	ENSG00000188283	1.32880311	2.3811E-07
POLR3GL	ENSG00000121851	-1.09504038	2.4983E-07
AL356585.2	ENSG00000279124	1.57681817	2.5312E-07
KRT8P3	ENSG00000254285	-1.31410231	2.5782E-07
PLEKHH2	ENSG00000152527	-1.20741330	2.6121E-07
SNAP25-AS1	ENSG00000227906	-2.04364424	2.7103E-07
TRIM31	ENSG00000204616	-2.20900676	2.7275E-07
ZFP28	ENSG00000196867	1.06138829	2.7732E-07
ZNF16	ENSG00000170631	1.14279713	2.8477E-07
ADAM19	ENSG00000135074	-1.58759298	3.1804E-07
RNASEL	ENSG00000135828	-1.33045688	3.2012E-07
ZBTB32	ENSG00000011590	-1.17938718	3.2075E-07
BCL6	ENSG00000113916	-1.07507228	3.2401E-07
HYKK	ENSG00000188266	-1.41163643	3.2427E-07
SH3BP1	ENSG00000100092	-1.93238590	3.3945E-07
RNF103-CHM	ENSG00000249884	3.52373107	3.4928E-07
ADAMTS16	ENSG00000145536	1.547529	3.547E-07
HIST1H2BC	ENSG00000180596	1.38413232	3.6498E-07
EFHD1	ENSG00000115468	-2.03060292	3.819E-07
ACOX2	ENSG00000168306	-1.71222475	3.8202E-07
N4BP2	ENSG00000078177	1.13612018	3.9318E-07
UBXN11	ENSG00000158062	1.07503947	3.996E-07
GPR3	ENSG00000181773	1.33632414	4.1401E-07
OLAH	ENSG00000152463	2.44317041	4.3376E-07
ZNF805	ENSG00000204524	1.38941702	4.4975E-07
VASH1	ENSG00000071246	-1.35188441	4.6207E-07
BACE2	ENSG00000182240	-1.05241664	4.6802E-07
SENP3-EIF4A	ENSG00000277957	1.2702012	4.7047E-07
AC010997.3	ENSG00000270087	-Inf	4.845E-07
IL32	ENSG00000008517	1.25018603	5.0124E-07
ACHE	ENSG00000087085	1.31732443	5.1729E-07
KIF26A	ENSG00000066735	-1.13398540	5.1992E-07
HACD2	ENSG00000206527	1.60149739	5.254E-07
NUDT7	ENSG00000140876	-1.59427733	5.2554E-07
TRIM36	ENSG00000152503	1.38984873	5.3111E-07
RF00096	ENSG00000239148	-1.51152067	5.4625E-07
STARD8	ENSG00000130052	-1.97704496	5.4783E-07
PXMP4	ENSG00000101417	-1.06853216	5.5722E-07
MICALL1	ENSG00000100139	1.222693	5.8929E-07
HIST2H4A	ENSG00000270882	1.9003625	6.1134E-07
GNAL	ENSG00000141404	1.39033833	6.1558E-07
ZNF792	ENSG00000180884	-1.86082808	6.1825E-07
MB	ENSG00000198125	-2.22261385	6.2054E-07
RUNX3	ENSG00000020633	-1.01643826	6.3779E-07
RAB3A	ENSG00000105649	-3.01397962	6.7286E-07
PLXNB3	ENSG00000198753	1.35659155	6.8429E-07
GNAZ	ENSG00000128266	-1.36035293	7.0583E-07
GSEC	ENSG00000280832	-2.09416811	7.1305E-07
RRP7BP	ENSG00000182841	-1.17834395	7.3611E-07
AC092327.2	ENSG00000269826	2.01371072	7.5997E-07
VSTM2L	ENSG00000132821	-2.27441247	7.6361E-07
GJB3	ENSG00000188910	2.25309927	7.9272E-07
DCDC2C	ENSG00000214866	3.04343397	8.0635E-07
AC008105.3	ENSG00000267121	-1.62064038	9.2446E-07
CAVIN4	ENSG00000170681	2.12592468	9.6033E-07
FO681492.1	ENSG00000277758	-1.77542379	9.6119E-07

ZNF416	ENSG00000083817	1.08584055	9.6348E-07
FXYD6	ENSG00000137726	-1.87423703(	9.829E-07
JAK3	ENSG00000105639	-1.49722928	9.8499E-07
OCEL1	ENSG00000099330	-1.68096403	1.0147E-06
AC107959.2	ENSG00000246130	2.75783629	1.0154E-06
PDE7B	ENSG00000171408	-1.37695919	1.0416E-06
OSGEPL1	ENSG00000128694	-1.06930487(	1.0518E-06
RP9P	ENSG00000205763	1.02922875	1.109E-06
MACROD1	ENSG00000133315	-1.21945625(	1.1095E-06
NXN	ENSG00000167693	-1.13403083	1.1131E-06
SEPT7P2	ENSG00000214765	1.04227054	1.145E-06
ZNF57	ENSG00000171970	1.37379229	1.1763E-06
KHK	ENSG00000138030	-1.17439168	1.1913E-06
PEA15	ENSG00000162734	1.08321926	1.3137E-06
PLCL2	ENSG00000154822	1.08055179	1.3215E-06
CTNNBIP1	ENSG00000178585	-1.13701633	1.326E-06
ASRGL1	ENSG00000162174	-1.27689152	1.3261E-06
ZBTB21	ENSG00000173276	1.37000958	1.3349E-06
C4BPA	ENSG00000123838	-2.85386806(	1.3407E-06
ANXA9	ENSG00000143412	-2.47950424(	1.4356E-06
ZNF565	ENSG00000196357	1.29523543	1.4588E-06
TLE6	ENSG00000104953	-2.51393736(	1.5941E-06
SLC17A1	ENSG00000124568	-3.48497790	1.6711E-06
GATA3	ENSG00000107485	1.55482559	1.7272E-06
NES	ENSG00000132688	-1.96505348	1.737E-06
CD300LB	ENSG00000178789	4.08058162	1.8066E-06
PDIK1L	ENSG00000175087	1.0777716	1.8375E-06
LRRC4B	ENSG00000131409	-2.02634049(	1.9047E-06
KBTBD11-OT	ENSG00000283239	2.56276176	1.9415E-06
TMEM198	ENSG00000188760	1.43715599	2.0121E-06
BCO1	ENSG00000135697	-1.60033980(	2.0871E-06
C2orf72	ENSG00000204128	-1.16725656(	2.0916E-06
ZNF543	ENSG00000178229	1.19165588	2.1084E-06
RGPD6	ENSG00000183054	1.22102869	2.137E-06
SAMHD1	ENSG00000101347	-1.06219462	2.2048E-06
PDE4B	ENSG00000184588	-1.29074792	2.2155E-06
NGF	ENSG00000134259	2.42802105	2.2183E-06
EGR2	ENSG00000122877	2.27190923	2.2722E-06
FBXO2	ENSG00000116661	-1.86243065	2.5129E-06
MIR194-2HG	ENSG00000229719	-1.72637578	2.5482E-06
AC087388.1	ENSG00000262251	1.32909931	2.6126E-06
VANGL2	ENSG00000162738	-1.08216355	2.6391E-06
SLX1A-SULT1	ENSG00000213599	-1.01414749	2.7997E-06
ZNF350	ENSG00000256683	1.20815206	2.8583E-06
ABLIM3	ENSG00000173210	2.23309298	2.8956E-06
RNA5-8SN3	ENSG00000278189	3.05878462	3.1047E-06
CTSV	ENSG00000136943	-1.22746333(	3.1202E-06
UNC5CL	ENSG00000124602	-2.02784615	3.1319E-06
ZNF222	ENSG00000159885	1.08344149	3.2069E-06
C1R	ENSG00000159403	-1.33827536	3.2331E-06
C10orf111	ENSG00000176236	3.20039267	3.2409E-06
GAMT	ENSG00000130005	-1.05668896	3.3476E-06
AC004918.3	ENSG00000270157	-1.42438489	3.4496E-06
LINC00313	ENSG00000185186	-2.25226785	3.5463E-06
NR2F1	ENSG00000175745	-1.08428229	3.5534E-06
GCSAM	ENSG00000174500	1.64104676	3.8097E-06
FAM171A2	ENSG00000161682	-1.65112030(	3.865E-06
ONECUT2	ENSG00000119547	-1.02937305	4.0684E-06

ZNF502	ENSG00000196653	1.37549723	4.0852E-06
AC120057.3	ENSG00000279641	2.51708562	4.0999E-06
GSTA4	ENSG00000170899	-1.10712283	4.1333E-06
CLYBL	ENSG00000125246	-1.53612686	4.4416E-06
CD37	ENSG00000104894	-2.43361072	4.4633E-06
CHST2	ENSG00000175040	1.87801963	4.626E-06
DHRS9	ENSG00000073737	2.468521	4.7231E-06
CHMP4BP1	ENSG00000258469	3.40058947	4.7536E-06
COX10-AS1	ENSG00000236088	-1.18349962	4.8066E-06
ZNF426	ENSG00000130818	1.16223253	4.9273E-06
ZBTB8A	ENSG00000160062	1.27616733	4.9675E-06
LGI3	ENSG00000168481	-2.37748613	5.3506E-06
AL031009.1	ENSG00000278987	1.94082439	5.4633E-06
RAD54B	ENSG00000197275	-1.00293053	5.5036E-06
PRODH2	ENSG00000250799	-2.94596016	5.5131E-06
CCDC85B	ENSG00000175602	1.03779421	5.5195E-06
LFNG	ENSG00000106003	-1.35962740	5.5372E-06
CRYM-AS1	ENSG00000189149	1.9147328	5.9628E-06
IDI1	ENSG00000067064	1.0434381	6.1942E-06
BIVM	ENSG00000134897	-1.15692523	6.2134E-06
DOC2A	ENSG00000149927	-1.76844236	6.2805E-06
AC012360.3	ENSG00000272994	1.17381251	6.3674E-06
HPDL	ENSG00000186603	-1.44103536	6.53E-06
TRIM34	ENSG00000258659	-1.20864090	6.5636E-06
BMI1	ENSG00000168283	1.13390806	7.1435E-06
NPIPA3	ENSG00000224712	5.49881069	7.28E-06
ZNF460	ENSG00000197714	1.23874354	7.353E-06
TRIM2	ENSG00000109654	-1.17906748	7.3578E-06
URGCP-MRP	ENSG00000270617	Inf	7.5314E-06
AP000523.1	ENSG00000215270	-1.84598983	7.5435E-06
ZBTB42	ENSG00000179627	-1.09117496	7.6127E-06
DEDD2	ENSG00000160570	1.03462868	7.8123E-06
COL17A1	ENSG00000065618	1.50965748	7.956E-06
LINC00346	ENSG00000255874	1.82440919	7.9885E-06
LINC01123	ENSG00000204588	-2.04892455	8.039E-06
AK7	ENSG00000140057	-1.25386086	8.5718E-06
PDZK1	ENSG00000174827	-1.32996996	9.2013E-06
HIST2H2BF	ENSG00000203814	1.47585443	9.2286E-06
GPR35	ENSG00000178623	-1.55632848	9.3093E-06
FOXD1	ENSG00000251493	1.14367412	9.4866E-06
AL353743.1	ENSG00000165121	1.66716442	1.0214E-05
AL441992.1	ENSG00000223478	-1.76464529	1.0558E-05
ZNF569	ENSG00000196437	1.05930124	1.0661E-05
NKAIN4	ENSG00000101198	-1.71659763	1.0973E-05
LACC1	ENSG00000179630	1.10127101	1.1056E-05
TLN2	ENSG00000171914	-1.02117083	1.1173E-05
AC005696.1	ENSG00000262050	-Inf	1.1622E-05
ZNF268	ENSG00000090612	1.00223775	1.1773E-05
MFSD3	ENSG00000167700	-1.35539671	1.1886E-05
SLC9A3R1	ENSG00000109062	-1.55876635	1.1893E-05
RASSF10	ENSG00000189431	-1.51180879	1.1948E-05
GPR146	ENSG00000164849	2.23874967	1.2083E-05
HIST1H4H	ENSG00000158406	1.00031587	1.24E-05
HS3ST3B1	ENSG00000125430	-1.49735911	1.2535E-05
ZNF639	ENSG00000121864	1.08049195	1.2748E-05
AC015912.3	ENSG00000274213	1.33321812	1.2848E-05
KSR2	ENSG00000171435	1.04378716	1.3013E-05
ZNF75A	ENSG00000162086	1.2557698	1.3548E-05

CCNE2	ENSG00000175305	-1.12633557	1.3582E-05
ZNF140	ENSG00000196387	1.42048587	1.3965E-05
NEURL3	ENSG00000163121	1.05672111	1.4097E-05
RNF185	ENSG00000138942	1.52568843	1.4402E-05
AC099509.1	ENSG00000249746	-4.11393623	1.56E-05
AC133681.1	ENSG00000239620	4.22226917	1.5656E-05
LIMS2	ENSG00000072163	1.24251242	1.586E-05
BCL11B	ENSG00000127152	-1.68640339	1.6163E-05
ABHD6	ENSG00000163686	-1.05613444	1.6171E-05
TYRO3P	ENSG00000259581	2.93474886	1.6807E-05
C3orf18	ENSG00000088543	-1.27710181	1.6975E-05
KCNK3	ENSG00000171303	-1.03076894	1.7101E-05
DUSP9	ENSG00000130829	-2.91844485	1.7456E-05
TMEM88	ENSG00000167874	2.93936425	1.7526E-05
ZNF620	ENSG00000177842	1.20772968	1.7766E-05
SNX15	ENSG00000110025	1.16212442	1.8205E-05
SRPX	ENSG00000101955	-1.22752320	1.8708E-05
CEP85L	ENSG00000111860	1.05316414	1.897E-05
LPXN	ENSG00000110031	1.06000735	1.9031E-05
GALNT6	ENSG00000139629	-1.20147561	1.9353E-05
CKMT1B	ENSG00000237289	-1.52960620	2.1637E-05
CCL24	ENSG00000106178	-4.72320789	2.1739E-05
DNAJB5	ENSG00000137094	1.23559926	2.2144E-05
IL22RA1	ENSG00000142677	-1.16397378	2.2471E-05
MRAS	ENSG00000158186	1.41622946	2.259E-05
BEX4	ENSG00000102409	1.54291568	2.2792E-05
MAGEH1	ENSG00000187601	-1.24276147	2.3088E-05
ARG2	ENSG00000081181	2.05404488	2.3239E-05
UGT1A2P	ENSG00000228445	-2.80698982	2.4065E-05
PDXK	ENSG00000160209	-1.03013296	2.411E-05
SDHAP2	ENSG00000215837	1.02953098	2.4306E-05
AL603756.1	ENSG00000271933	1.29899458	2.4606E-05
HYAL4	ENSG00000106302	-2.34232216	2.5557E-05
AC009299.1	ENSG00000225813	1.48854549	2.6401E-05
EBI3	ENSG00000105246	-1.27808966	2.6428E-05
TFEB	ENSG00000112561	-1.05287622	2.8589E-05
CARF	ENSG00000138380	-1.29049411	2.8786E-05
LINC01504	ENSG00000225434	-1.54535117	3.2275E-05
AL031714.1	ENSG00000261505	1.11464813	3.2312E-05
LINC01433	ENSG00000230176	-1.75184452	3.3058E-05
KCNH1	ENSG00000143473	1.25726008	3.392E-05
CTSH	ENSG00000103811	-1.20584036	3.5039E-05
HIVEP1	ENSG00000095951	1.40882214	3.5073E-05
EGR4	ENSG00000135625	1.64843422	3.6355E-05
AC093525.8	ENSG00000279520	1.63277084	3.6792E-05
C11orf71	ENSG00000180425	-1.21922529	3.9881E-05
TMEM165	ENSG00000134851	-1.21410197	4.0514E-05
SNU13	ENSG00000100138	-1.30952605	4.1092E-05
MFSD13A	ENSG00000138111	-1.03934144	4.1695E-05
PDP1	ENSG00000164951	1.17659146	4.3611E-05
TMEM107	ENSG00000179029	-1.05629464	4.4789E-05
FP671120.3	ENSG00000280800	1.08175758	4.5275E-05
NIPAL4	ENSG00000172548	1.04006058	4.6183E-05
PROC	ENSG00000115718	-1.80044794	4.6477E-05
TM4SF5	ENSG00000142484	-3.19683317	4.8441E-05
C21orf58	ENSG00000160298	-1.00948839	4.9853E-05
ANXA10	ENSG00000109511	3.41823726	5.2083E-05
CBX7	ENSG00000100307	-1.13839591	5.2145E-05

IFITM2	ENSG00000185201	-1.18784556	5.2389E-05
AC068946.2	ENSG00000284820	-1.70848035	5.3725E-05
AC008105.2	ENSG00000233483	-2.67027626	5.4576E-05
MAP1LC3B2	ENSG00000258102	1.50702497	5.6498E-05
DEPTOR	ENSG00000155792	-2.05696918	5.7526E-05
AC138150.2	ENSG00000267288	-1.17640871	5.8247E-05
TMPO-AS1	ENSG00000257167	-1.24378108	6.1686E-05
AL139099.1	ENSG00000258377	Inf	6.2266E-05
KCNH3	ENSG00000135519	-1.38168993	6.2929E-05
AL645608.9	ENSG00000273443	1.72309963	6.2996E-05
CFD	ENSG00000197766	-2.04873169	6.4197E-05
HOXB13	ENSG00000159184	-1.40178204	6.4754E-05
BHLHA15	ENSG00000180535	1.53644861	6.4984E-05
SCNN1A	ENSG00000111319	-1.32545718	6.5351E-05
S1PR5	ENSG00000180739	-3.06321566	6.5944E-05
TRAPPC2B	ENSG00000256060	1.59768891	6.8028E-05
AC004520.1	ENSG00000273237	1.85724247	6.8521E-05
ATXN7L2	ENSG00000162650	1.63167496	6.8646E-05
AC073611.1	ENSG00000257605	2.27102291	6.8991E-05
TUB	ENSG00000166402	-1.31996937	6.9628E-05
AL121944.1	ENSG00000272009	2.88100182	6.9803E-05
LSMEM1	ENSG00000181016	1.99700627	7.0263E-05
FER	ENSG00000151422	1.92296819	7.0675E-05
AC008105.1	ENSG00000233175	-1.86848509	7.1033E-05
LCAT	ENSG00000213398	1.18449105	7.434E-05
ARHGEF16	ENSG00000130762	-1.09044460	7.661E-05
TMEM121	ENSG00000184986	-1.83811385	7.7776E-05
EGOT	ENSG00000235947	2.08331321	7.7782E-05
AC019205.1	ENSG00000229852	-1.53940577	7.8434E-05
ZUFSP	ENSG00000153975	1.05539174	8.0131E-05
TSPAN14	ENSG00000108219	-1.81150622	8.2918E-05
FAM53C	ENSG00000120709	1.5525908	8.3153E-05
BTN2A3P	ENSG00000124549	1.19784355	8.5182E-05
IGFBP1	ENSG00000146678	1.28363298	8.8984E-05
TMEM170B	ENSG00000205269	-1.03006941	8.9964E-05
RPL12P14	ENSG00000224321	1.40174663	9.0755E-05
ANKAR	ENSG00000151687	1.29143816	9.1658E-05
ZFYVE28	ENSG00000159733	-1.10685778	9.409E-05
SULT1A4	ENSG00000213648	-1.66532729	9.547E-05
ZNF30	ENSG00000168661	-1.06539073	9.6433E-05
CD83	ENSG00000112149	1.21792806	9.6661E-05
AMACR	ENSG00000242110	-1.21893636	0.00010182
RDH5	ENSG00000135437	-2.01491599	0.00010369
UBE2Q2P1	ENSG00000189136	1.07837705	0.0001116
AC069281.2	ENSG00000274272	1.73515523	0.00011336
BCL2A1	ENSG00000140379	1.24181993	0.00011456
KCNJ2	ENSG00000123700	-1.52646539	0.00011491
AC017116.1	ENSG00000239775	-Inf	0.00011587
RTP4	ENSG00000136514	-3.99774539	0.00012181
NPM1P26	ENSG00000235677	2.21574675	0.00012393
AL645608.3	ENSG00000230699	-1.96347333	0.00012458
PAQR8	ENSG00000170915	-1.16083167	0.00012583
F3	ENSG00000117525	1.27204728	0.00012684
ZFAND2A	ENSG00000178381	1.0673958	0.00012732
AC023632.6	ENSG00000280123	-1.07375208	0.00012832
NPY1R	ENSG00000164128	-1.46003243	0.00012835
AC133644.2	ENSG00000273445	-2.88431055	0.00012846
AC140479.2	ENSG00000230650	-1.22929232	0.00012969

LINC02212	ENSG00000249396	-1.071280782	0.00013121
AC004233.3	ENSG00000272079	1.85918374	0.00013207
DMBX1	ENSG00000197587	-1.964432805	0.00013371
GRIN3B	ENSG00000116032	-1.094607409	0.00013375
CD14	ENSG00000170458	-1.814593778	0.00013494
AL590399.1	ENSG00000204802	1.38655932	0.00013504
HPSE	ENSG00000173083	-1.335930665	0.00013738
ARHGAP24	ENSG00000138639	-1.105648613	0.0001422
PCDHAC1	ENSG00000248383	-1.464057872	0.00014642
CPSF1P1	ENSG00000214076	-1.444962650	0.00014646
AC078880.3	ENSG00000271579	3.99492483	0.00014924
REEP6	ENSG00000115255	-1.211652033	0.00015665
TMEM74B	ENSG00000125895	-1.561618218	0.00015806
AXIN2	ENSG00000168646	-1.289621727	0.00015817
MIR600HG	ENSG00000236901	-1.172929950	0.00016368
PLPPR3	ENSG00000129951	-1.460643046	0.00017425
PRRT1B	ENSG00000283526	-2.810353271	0.00017783
IL18R1	ENSG00000115604	-1.028239369	0.00019103
LINC-PINT	ENSG00000231721	1.35750726	0.00019169
MZF1-AS1	ENSG00000267858	1.02139293	0.00019971
RCBTB2	ENSG00000136161	1.71582952	0.00020128
BX640514.2	ENSG00000273812	5.07942209	0.00021116
PRPS2	ENSG00000101911	-1.127994661	0.000214
PRICKLE4	ENSG00000278224	-2.583835477	0.00021642
CSF2	ENSG00000164400	3.00312515	0.00021906
CEMIP	ENSG00000103888	1.20304127	0.00021946
RNFT2	ENSG00000135119	-1.003762713	0.00021982
TBC1D5	ENSG00000131374	-1.153641263	0.00022015
HLA-DMA	ENSG00000204257	-1.657644313	0.00022527
KRT20	ENSG00000171431	-2.463235685	0.00022736
SLC7A5P1	ENSG00000260727	1.41067992	0.00022821
CCDC149	ENSG00000181982	1.08388549	0.00022997
AC112220.4	ENSG00000271643	1.59039913	0.00023211
AC004890.2	ENSG00000244560	-1.258346229	0.00023517
PYROXD2	ENSG00000119943	-1.015174623	0.00023836
BMP4	ENSG00000125378	-1.207884961	0.00024547
AP000580.1	ENSG00000219529	2.14635929	0.00024883
LINC01881	ENSG00000220804	1.02051588	0.00024984
NRG2	ENSG00000158458	-1.241837990	0.00026045
AC026124.2	ENSG00000276853	3.02658361	0.00026286
LINC01719	ENSG00000233396	-1.640590746	0.00026476
RNA5-8SN5	ENSG00000274917	2.53142007	0.00027435
DPEP1	ENSG00000015413	-2.363890294	0.00027522
FCGRT	ENSG00000104870	-1.515117123	0.00027867
LINC00488	ENSG00000214381	-1.526578018	0.00028716
APC	ENSG00000134982	-1.323505375	0.00028881
PIP5K1A	ENSG00000143398	1.28052995	0.00029073
AC008915.2	ENSG00000260136	1.18939562	0.00029473
RGS17P1	ENSG00000229473	3.36382279	0.00029771
SPNS2	ENSG00000183018	1.09823522	0.00030547
MAFF	ENSG00000185022	1.62285379	0.0003073
ZNF331	ENSG00000130844	1.03184064	0.00030731
HIST1H4E	ENSG00000276966	1.99279197	0.00031924
TMEM164	ENSG00000157600	-1.026461205	0.00032302
AKR1B10P1	ENSG00000213606	-1.165292287	0.00032875
CCDC150P1	ENSG00000256304	1.01332702	0.00032962
CLDN9	ENSG00000213937	2.52706349	0.00033309
TTLL6	ENSG00000170703	-1.404424950	0.00034693

AC062029.1	ENSG00000234028	1.54299418	0.00036589
AC025171.2	ENSG00000215068	-1.17316271	0.0003663
FERMT2	ENSG00000073712	1.04301367	0.00036802
SMG7-AS1	ENSG00000232860	1.19690161	0.00036903
QPRT	ENSG00000103485	-2.33844812	0.00037294
EGFR-AS1	ENSG00000224057	1.76648218	0.00037434
CLEC11A	ENSG00000105472	-1.27528213	0.00038077
AL662890.1	ENSG00000225173	Inf	0.00038187
LINC01468	ENSG00000231131	-3.18652825	0.0003827
S100A2	ENSG00000196754	1.08448898	0.00039497
FGF19	ENSG00000162344	3.75741085	0.00040051
PIK3R5	ENSG00000141506	1.07513773	0.0004027
SPRR2D	ENSG00000163216	4.29000569	0.0004032
ALOX12P2	ENSG00000262943	1.04787301	0.00042922
LINC01521	ENSG00000213888	-1.08182010	0.00043753
JRK	ENSG00000234616	-1.37013338	0.00045501
PTX3	ENSG00000163661	1.02846087	0.00045839
LYRM9	ENSG00000232859	-1.42060534	0.00047505
FRAT1	ENSG00000165879	-1.19881805	0.00048048
AC090181.2	ENSG00000269951	1.54415191	0.0004901
AC100757.1	ENSG00000273679	-1.58076568	0.00049322
LINC02482	ENSG00000251580	-4.09422774	0.00049382
MIR222HG	ENSG00000270069	1.81066983	0.00050429
AP000640.1	ENSG00000254477	4.24699392	0.00050496
PGM5P2	ENSG00000277778	1.88512313	0.00051001
RPSAP52	ENSG00000241749	3.45779624	0.00051173
AC003092.1	ENSG00000236453	3.64071888	0.0005189
MIR193BHG	ENSG00000262454	-1.26154591	0.00052707
AS3MT	ENSG00000214435	-1.19980871	0.00053476
RGCC	ENSG00000102760	-4.51103779	0.00054607
GALK1	ENSG00000108479	-1.07158490	0.00055164
FOXN3	ENSG00000053254	-1.23765354	0.0005551
KLF15	ENSG00000163884	2.77484084	0.00056375
AC023043.1	ENSG00000260552	-2.79399476	0.00056504
SLC4A4	ENSG00000080493	-1.00940371	0.00056624
CCL4L2	ENSG00000276070	1.10516628	0.00056801
AC106886.2	ENSG00000260899	1.6411805	0.00056853
AUH	ENSG00000148090	-1.14983373	0.00056972
SPATA32	ENSG00000184361	-1.84014417	0.00057709
AC092868.1	ENSG00000259674	-1.81434500	0.00057842
AC034213.1	ENSG00000250509	1.13524687	0.00060119
AOC2	ENSG00000131480	1.00294883	0.00060193
EMC3-AS1	ENSG00000180385	2.36631468	0.00064581
ZNF614	ENSG00000142556	1.08714522	0.00065792
IFNL4	ENSG00000272395	-2.59081097	0.00066012
AD000671.2	ENSG00000267120	2.14426609	0.00067101
LINC01347	ENSG00000214837	1.46211344	0.000686
PURPL	ENSG00000250337	1.46202251	0.00068616
ASGR1	ENSG00000141505	-1.59822359	0.00069073
HOXB-AS3	ENSG00000233101	-1.49297298	0.00070922
ACKR3	ENSG00000144476	1.35912546	0.00071875
CRYM	ENSG00000103316	-1.04826307	0.00073024
CYP2U1	ENSG00000155016	-1.20638687	0.00075164
SYDE2	ENSG00000097096	1.0166766	0.00075816
MUC1	ENSG00000185499	-1.06475345	0.00076167
TRIM46	ENSG00000163462	-1.05312298	0.00077192
HBQ1	ENSG00000086506	-1.42328028	0.00078831
HIST1H4C	ENSG00000197061	1.40853343	0.00082602

RASAL2-AS1	ENSG00000224687	1.5380192	0.0008458
SLCO1B7	ENSG00000205754	1.01821002	0.00084667
AC124276.1	ENSG00000254991	3.19783414	0.00085411
HIST1H2BJ	ENSG00000124635	1.01530202	0.00085992
ZNF295-AS1	ENSG00000237232	2.53183729	0.00086074
LAT2	ENSG00000086730	1.06928642	0.00087512
ACTN2	ENSG00000077522	1.42001398	0.00088045
CCDC69	ENSG00000198624	-1.42300570(	0.00089315
MAP1LC3B	ENSG00000140941	1.34929571	0.00090972
AC004067.1	ENSG00000273447	-Inf	0.00092609
LINC01355	ENSG00000261326	-1.03291968	0.00092935
PROB1	ENSG00000228672	-1.22819694	0.00093141
LINC01547	ENSG00000183250	-1.00739498	0.00094269
GLUD1P3	ENSG00000250959	1.79927173	0.00094989
AC004918.1	ENSG00000244701	-1.79008593	0.00096003
IFITM3	ENSG00000142089	-1.43991459	0.00096073
AC011247.2	ENSG00000235586	Inf	0.00097403
MAPK7	ENSG00000166484	1.18687214	0.00098097
ACY3	ENSG00000132744	-2.19647596(	0.00098185
PRKAR2B	ENSG00000005249	-1.26799031	0.00099189
AC005046.1	ENSG00000273055	-1.59014251	0.00100577
RNA5-8S5	ENSG00000277739	2.47748615	0.00100732
EGLN2	ENSG00000269858	1.26698468	0.00101151
BMS1P22	ENSG00000232775	-1.43222596	0.00102769
TTC39C	ENSG00000168234	-1.07836947	0.00104993
DNAAF3	ENSG00000167646	-1.04735679	0.00107015
AC010655.4	ENSG00000273184	-Inf	0.00107401
AC025884.1	ENSG00000258732	1.87313579	0.00108294
ZNF530	ENSG00000183647	1.31065251	0.00109563
IL11RA	ENSG00000137070	-1.08202185	0.00110353
BAAT	ENSG00000136881	-1.29703608	0.00115466
AC145098.2	ENSG00000279821	1.45535535	0.00116536
OSER1-AS1	ENSG00000223891	1.62768518	0.00121911
PAOX	ENSG00000148832	-1.31767346(	0.00125543
CYGB	ENSG00000161544	-1.90782920	0.00127386
AC092718.3	ENSG00000260643	1.4220634	0.00128025
FILIP1L	ENSG00000168386	1.05570281	0.00135256
LINC01942	ENSG00000253428	-2.80107705	0.00138501
RARB	ENSG00000077092	-1.84222568	0.00138721
HACD4	ENSG00000188921	-1.02289565	0.00142195
PRPS1P2	ENSG00000232630	1.44557684	0.00145289
CCDC159	ENSG00000183401	-1.13300335	0.0015035
AC090192.2	ENSG00000253227	1.58412673	0.00152771
NEURL1B	ENSG00000214357	-2.21044715	0.00153127
RAB26	ENSG00000167964	-1.53889467(	0.00154296
RPS26P6	ENSG00000212994	1.44838836	0.00156768
CCL3	ENSG00000277632	1.28362569	0.00157987
AC008687.6	ENSG00000269706	4.75814114	0.00161082
AC009501.1	ENSG00000231609	-1.57931833	0.00161473
FRMD6-AS1	ENSG00000273888	1.49305825	0.00161475
ID1	ENSG00000125968	-2.17092018	0.00162036
KRT83	ENSG00000170523	-2.02012726	0.00162769
P2RY1	ENSG00000169860	-1.05171727	0.00163263
TBC1D3G	ENSG00000260287	1.21753434	0.00164079
AP001273.1	ENSG00000279696	1.04331104	0.00164253
AP003119.1	ENSG00000254632	-1.75336297	0.00168059
CEBPA-AS1	ENSG00000267296	-1.31106744	0.00173529
MNX1-AS1	ENSG00000243479	-1.08486839	0.00180646

AL353625.1	ENSG00000213073	1.68935853	0.00185465
TUBAL3	ENSG00000178462	-1.22055603	0.00186655
PGPEP1	ENSG00000130517	-2.22834604	0.00186818
AC018628.1	ENSG00000279133	1.09815884	0.00188785
PPIAP30	ENSG00000206448	Inf	0.00192023
ZNF721	ENSG00000182903	1.08209513	0.00192478
AL662899.2	ENSG00000263020	1.27203076	0.00194546
AC011466.3	ENSG00000269534	-3.72558260	0.00194755
C2CD4C	ENSG00000183186	-2.44663502	0.00195096
HERPUD1	ENSG00000051108	1.42520751	0.0019695
LINC00261	ENSG00000259974	-1.08343509	0.00197566
CYP2T1P	ENSG00000233622	1.28136428	0.00197719
AC073957.3	ENSG00000273151	1.06301294	0.00200342
ZNF256	ENSG00000152454	1.43826907	0.00204247
H19	ENSG00000130600	-1.28578149	0.00209151
ULK4P2	ENSG00000260128	1.24475164	0.00212966
AC022816.1	ENSG00000230647	3.77213499	0.00225846
RASAL2	ENSG00000075391	1.83167445	0.00227989
CFHR1	ENSG00000244414	-2.19933931	0.00229732
AL139289.1	ENSG00000229431	2.9560307	0.00233105
CLPSL2	ENSG00000196748	-Inf	0.00233481
RDM1	ENSG00000278023	-1.60808313	0.00234718
AC005261.5	ENSG00000279541	1.66265371	0.00237977
AL122035.2	ENSG00000272909	-1.39440245	0.0023981
HIST3H2BB	ENSG00000196890	1.55377594	0.00242296
AC091057.5	ENSG00000284906	1.680232	0.00243973
SERPINB3	ENSG00000057149	-1.63865719	0.00246137
TDRKH-AS1	ENSG00000203288	1.41168745	0.0024891
PPP1R18	ENSG00000146112	1.3576299	0.00260994
MPST	ENSG00000128309	-1.48107670	0.00264862
F2	ENSG00000180210	-1.31274962	0.00265048
LINC02057	ENSG00000249279	1.62185447	0.00265992
FNDC10	ENSG00000228594	-1.29611966	0.00266219
COLCA2	ENSG00000214290	-1.51875613	0.00286325
IQCD	ENSG00000166578	-1.43500507	0.00287915
NPIPP1	ENSG00000188599	1.34493476	0.00289061
ZBTB45P1	ENSG00000225108	-1.01142422	0.00290498
FOXD3	ENSG00000187140	1.32159127	0.00299747
KRT14	ENSG00000186847	-1.24888328	0.00300255
AC026368.1	ENSG00000277840	-2.03194298	0.00306593
S100A9	ENSG00000163220	-2.35960258	0.00306976
AP000924.1	ENSG00000254416	-3.74868182	0.00310197
CPT1C	ENSG00000169169	-1.07787167	0.00315618
RF00100	ENSG00000202198	Inf	0.00316672
TIMD4	ENSG00000145850	1.12377454	0.00317536
AL139393.2	ENSG00000272841	1.67711199	0.00318018
HOXD11	ENSG00000128713	-1.95839317	0.00319709
RIBC2	ENSG00000128408	-1.81648423	0.00322645
SLC36A1	ENSG00000123643	-1.06643753	0.00326236
CHST13	ENSG00000180767	-1.74103215	0.00326948
AC048341.2	ENSG00000275180	-1.04321353	0.0033535
CKMT1A	ENSG00000223572	-1.12018788	0.00342992
TSSC4	ENSG00000184281	1.07389597	0.00344434
AL589743.6	ENSG00000277156	-1.10035308	0.00349401
GAL	ENSG00000069482	1.15398114	0.00352646
CRABP2	ENSG00000143320	1.05094163	0.00361224
AL391056.1	ENSG00000227619	-1.00618661	0.00361729
CATSPERZ	ENSG00000219435	3.27480763	0.00362105

PHBP5	ENSG00000233523	2.28550546	0.00365317
TM4SF19	ENSG00000145107	1.27823711	0.00366578
LINC00431	ENSG00000225760	2.68300789	0.00368509
LINC02298	ENSG00000257556	-1.59375377[illegible]	0.00375695
RNA5-8SN2	ENSG00000273730	2.97406479	0.00378058
AD000671.3	ENSG00000267439	1.60729674	0.00379238
TNNI3	ENSG00000129991	-1.58739486[illegible]	0.00384643
AC122718.1	ENSG00000250461	1.05966963	0.00390692
CCDC9B	ENSG00000188549	-1.88346383[illegible]	0.0039076
HRH1	ENSG00000196639	1.08493851	0.00392866
ADM5	ENSG00000224420	1.30501661	0.00393802
AC008537.3	ENSG00000279108	-1.20871973[illegible]	0.00396871
SPDYE2	ENSG00000205238	-1.40663136[illegible]	0.00404328
AL845472.2	ENSG00000279561	-1.39574188[illegible]	0.00404382
AP000769.1	ENSG00000173727	-1.03309193[illegible]	0.00405327
ZNF615	ENSG00000197619	1.13764743	0.00409159
HEY1	ENSG00000164683	1.2442777	0.00411117
C1QL3	ENSG00000165985	-1.18644822[illegible]	0.00412423
KCNJ2-AS1	ENSG00000267365	-1.82939452[illegible]	0.00419312
AC159540.2	ENSG00000277701	1.41660091	0.00419353
TMEFF1	ENSG00000241697	1.97622245	0.00420479
AC009005.1	ENSG00000267751	-1.29202373[illegible]	0.00420489
SEPT5	ENSG00000184702	1.18293574	0.0042368
CDV3	ENSG00000091527	1.1568953	0.00428011
LINC00858	ENSG00000229404	-1.68054447[illegible]	0.00431903
AC132872.1	ENSG00000260563	-1.11750843[illegible]	0.00432106
AC116351.2	ENSG00000272347	2.46509973	0.00434185
Z95115.1	ENSG00000261188	-1.10662085[illegible]	0.00435575
SNORA78	ENSG00000273587	4.56834441	0.00447523
HIST4H4	ENSG00000197837	1.71079206	0.00449141
NALT1	ENSG00000237886	-1.84214051[illegible]	0.00458456
TMEM75	ENSG00000280055	1.76027518	0.00469903
AC027117.1	ENSG00000253671	-2.33179904[illegible]	0.00473074
PIK3CD-AS2	ENSG00000231789	-1.20145729[illegible]	0.00479421
RAP1AP	ENSG00000258769	Inf	0.00482969
ZNF830	ENSG00000198783	1.19289599	0.00487512
CDKL2	ENSG00000138769	1.20718689	0.00490147
IDH1-AS1	ENSG00000231908	-2.20341441[illegible]	0.00491317
SCG5	ENSG00000166922	1.24673696	0.00493849
VASH2	ENSG00000143494	-1.12365985[illegible]	0.00519791
MANSC1	ENSG00000111261	-1.90575972[illegible]	0.00523903
HIST2H3D	ENSG00000183598	1.89666945	0.00531444
TRAM1L1	ENSG00000174599	-1.42424217[illegible]	0.00533
AL589182.2	ENSG00000278594	-1.73540066[illegible]	0.00533802
GCHFR	ENSG00000137880	-2.85032209[illegible]	0.00534529
TTLL1	ENSG00000100271	-1.21262454[illegible]	0.00534692
ATP6V1B1	ENSG00000116039	-1.35039271[illegible]	0.00538671
ZNF264	ENSG00000083844	1.40869276	0.00539875
CTXN1	ENSG00000178531	-1.40379330[illegible]	0.00543638
GCNA	ENSG00000147174	1.4562277	0.00546719
MKL2	ENSG00000186260	-1.10558913[illegible]	0.00547573
SRRM5	ENSG00000226763	1.29493272	0.00548452
DRC3	ENSG00000171962	1.1053623	0.00560597
VAX1	ENSG00000148704	1.10994894	0.00563256
ELF3-AS1	ENSG00000234678	-1.03832010[illegible]	0.00565553
AC009812.1	ENSG00000251867	1.78327685	0.00565897
CNFN	ENSG00000105427	-1.42365153[illegible]	0.0056979
BASP1-AS1	ENSG00000215196	-1.49695764[illegible]	0.00575506

CXXC5	ENSG00000171604	-1.39718988	0.00576745
AC026803.2	ENSG00000267898	2.60791548	0.0058451
PWP2	ENSG00000241945	-1.25083454	0.00584899
AC008011.2	ENSG00000257042	3.04485571	0.00587531
AL121987.2	ENSG00000227741	1.48265414	0.00592978
HIST1H4K	ENSG00000273542	1.70880271	0.00593455
AC137936.2	ENSG00000283511	1.84841932	0.00595272
PTPRN2	ENSG00000155093	-1.04872880	0.00597751
C11orf96	ENSG00000187479	1.1450955	0.00602966
PPIAP2	ENSG00000227379	Inf	0.00603724
AC020915.1	ENSG00000267216	1.27668903	0.00604704
ZSCAN21	ENSG00000166529	1.01610872	0.00609752
AL161668.1	ENSG00000178107	-4.06285394	0.00613621
LINC01503	ENSG00000233901	-1.04548415	0.00620799
TERT	ENSG00000164362	-1.08526170	0.00625272
CCL3L1	ENSG00000276085	1.52833498	0.00631592
RTN4R	ENSG00000040608	1.25089738	0.00634274
AC009269.4	ENSG00000259744	2.64722253	0.00651157
AL731569.1	ENSG00000227896	-1.21161172	0.00660707
AC244230.2	ENSG00000285402	-2.08694556	0.00665134
TUBB3	ENSG00000258947	1.17187904	0.00671961
ZNF138	ENSG00000197008	-1.19638043	0.00678117
AC008533.1	ENSG00000285366	-3.47007494	0.00681565
TSPAN12	ENSG00000106025	-1.45026361	0.00682281
MARK2P8	ENSG00000239503	3.33463766	0.00684292
CCDC17	ENSG00000159588	1.32515559	0.00689061
AC072061.1	ENSG00000259826	2.11198918	0.00689108
AC016596.1	ENSG00000227908	1.45239893	0.00709305
CLDN4	ENSG00000189143	1.49794311	0.00712863
RPL4P6	ENSG00000230071	1.71791693	0.00727461
IL31RA	ENSG00000164509	1.04602172	0.00734754
SOCS2	ENSG00000120833	1.70679373	0.00738591
DFFBP1	ENSG00000232303	3.05692542	0.00741307
ACTG1P3	ENSG00000215388	2.9033165	0.00752462
AL807757.2	ENSG00000236095	-1.55732106	0.00752806
AC087269.1	ENSG00000254367	-1.90236133	0.00767305
VWA7	ENSG00000204396	-1.09603803	0.0078582
AL080317.3	ENSG00000272356	1.16570831	0.00792746
HSP90B2P	ENSG00000259706	1.46781279	0.00793568
CXCR4	ENSG00000121966	-1.67305732	0.0080977
AC073896.1	ENSG00000144785	-1.71288213	0.00820495
DHFR	ENSG00000228716	-1.04630759	0.00822841
AL354740.1	ENSG00000225339	2.93568466	0.00829122
GDPD1	ENSG00000153982	-1.04471576	0.00842944
TCN1	ENSG00000134827	-2.09425396	0.00850022
CHD2	ENSG00000173575	1.03156194	0.00859048
AL121772.3	ENSG00000276952	-1.14106168	0.00875373
AC108073.2	ENSG00000241011	3.09609908	0.00887858
BST2	ENSG00000130303	-2.16451712	0.00889441
LAGE3P1	ENSG00000225693	-2.29583014	0.00889927
TEN1	ENSG00000257949	1.8536527	0.0089446
SRP14-AS1	ENSG00000248508	-1.08957283	0.00907853
AC004471.1	ENSG00000223461	2.47650752	0.00910262
SPATA20P1	ENSG00000231123	-Inf	0.00933074
CYP4F3	ENSG00000186529	-1.86130155	0.00953482
SNAI2	ENSG00000019549	2.17150644	0.00965394
FAM229B	ENSG00000203778	-1.29932615	0.00967422
AP003392.4	ENSG00000255121	-1.39609897	0.00976549

EGFL8	ENSG00000241404	-1.49986903	0.00997268
SPEF1	ENSG00000101222	1.8211986	0.01030573
AC069224.1	ENSG00000260572	2.10151734	0.01051159
AC005393.1	ENSG00000276445	1.47998979	0.01082006
ARNTL2	ENSG00000029153	1.93834237	0.01088329
GSDMB	ENSG00000073605	-1.02080297	0.01111139
FTLP14	ENSG00000260459	-3.21903078	0.01124836
NSFP1	ENSG00000260075	-Inf	0.01155292
B3GNT5	ENSG00000176597	1.52199472	0.01161734
FOXO3B	ENSG00000240445	1.19774793	0.01164002
AL358113.1	ENSG00000285130	-1.03989672	0.01169616
TSN	ENSG00000211460	-1.30504632	0.01190404
AC012181.1	ENSG00000261114	1.69646913	0.01213964
PDE9A	ENSG00000160191	-1.23189335	0.01214468
AC244197.3	ENSG00000241489	-1.19637182	0.01215452
AC069544.1	ENSG00000272853	-1.46878561	0.01215847
HTR7P1	ENSG00000183935	-1.07182681	0.01224439
FAM35BP	ENSG00000165874	-1.60994441	0.01234812
AC021242.3	ENSG00000272267	1.65397105	0.01235074
RPS7P3	ENSG00000231940	2.64090717	0.0127183
RPL29P14	ENSG00000241112	4.2924962	0.01283576
AC092153.1	ENSG00000285155	-1.36031276	0.01298669
TSKU	ENSG00000182704	-1.14287846	0.0131096
GDPD3	ENSG00000102886	1.64643763	0.01396609
ZNF461	ENSG00000197808	1.74229184	0.01396758
AC026464.3	ENSG00000260371	-4.06843169	0.01406764
GAPLINC	ENSG00000266835	-2.20331774	0.01419526
AC007731.5	ENSG00000277971	1.49370025	0.0143874
RPL22P24	ENSG00000231084	1.82667863	0.01450668
NIPSNAP3B	ENSG00000165028	-1.28707788	0.01466515
AL606534.5	ENSG00000253326	1.22479385	0.01476442
MAP2K6	ENSG00000108984	-3.08788984	0.01501292
PFN2	ENSG00000070087	-1.07614659	0.01505029
AC087385.1	ENSG00000240163	1.40446145	0.01507388
AL138759.1	ENSG00000244332	-1.07627507	0.01511402
AC005154.3	ENSG00000244480	-2.43814730	0.01521608
CX3CL1	ENSG00000006210	-1.82865558	0.01530774
GPATCH2L	ENSG00000089916	1.22750838	0.01543521
AC011450.1	ENSG00000197813	-2.28278323	0.01565285
AC120114.1	ENSG00000247735	-1.29811994	0.01588365
AKR7A2P1	ENSG00000229020	2.47520686	0.01589265
AP001437.1	ENSG00000273210	3.59388757	0.01604888
SNHG9	ENSG00000255198	1.70829363	0.01607833
ROCK1P1	ENSG00000263006	1.10202369	0.01636782
AC108463.3	ENSG00000271590	1.90021069	0.01653999
ETV5-AS1	ENSG00000234197	3.57160334	0.0166479
INSIG1	ENSG00000186480	1.40761668	0.01664874
CYS1	ENSG00000205795	-1.08962847	0.01666268
AC010271.1	ENSG00000268366	1.46350109	0.01673258
SLC19A2	ENSG00000117479	1.28129429	0.01685912
AC092803.2	ENSG00000260805	1.57699291	0.01719059
AL391825.1	ENSG00000229808	1.68576425	0.01764241
BMS1P7	ENSG00000270025	2.01421995	0.01767193
YWHABP2	ENSG00000256464	2.55651557	0.01781584
NCCRP1	ENSG00000188505	-1.26807763	0.01785382
KRTAP5-1	ENSG00000205869	2.28432308	0.01801179
RPL13AP20	ENSG00000234498	1.14285951	0.01831132
AC055811.3	ENSG00000266498	2.94120037	0.01860298

ZNF195	ENSG00000005801	1.12727926	0.01867851
AL359183.1	ENSG00000279406	-1.50809936	0.01871332
ZNF223	ENSG00000178386	1.19552927	0.01896553
TUBA4B	ENSG00000243910	-1.87301142	0.01955439
RPS10-NUDT	ENSG00000270800	4.58544496	0.01967091
CARD8-AS1	ENSG00000268001	1.46772819	0.01977955
AC008429.1	ENSG00000204758	1.00795092	0.01996668
AP005136.2	ENSG00000266783	2.31267462	0.02002032
AC098487.1	ENSG00000248161	-1.31815449	0.02012807
LINC01843	ENSG00000251169	-1.03561157	0.02024813
LRRC8D	ENSG00000171492	-1.19289630	0.0203042
AL445183.3	ENSG00000236360	1.15830583	0.02075717
LRRC29	ENSG00000125122	-1.14423905	0.02084098
AL139260.1	ENSG00000228436	2.11400193	0.02089652
CR392039.3	ENSG00000279501	1.71110314	0.02091946
AP001574.1	ENSG00000253217	-2.14107063	0.0209671
AL157935.1	ENSG00000227218	2.12035595	0.02122161
MIR34AHG	ENSG00000228526	4.62268396	0.02122923
RCN3	ENSG00000142552	-1.32048955	0.02127134
AL021578.1	ENSG00000275894	1.65508265	0.02139521
ZBTB49	ENSG00000168826	1.23344529	0.02140101
NEURL2	ENSG00000124257	-1.23784709	0.02160691
AL445647.1	ENSG00000272046	-1.02828541	0.02199911
PSCA	ENSG00000167653	1.27294026	0.02208513
RAB31	ENSG00000168461	-1.15159947	0.02209574
AL356056.2	ENSG00000231187	-1.40905103	0.0221342
AL139424.2	ENSG00000284642	-1.82992508	0.02244123
AC004057.1	ENSG00000196656	1.29033017	0.0224904
AL138885.3	ENSG00000285278	1.26430593	0.02262489
ZNF641	ENSG00000167528	1.73206161	0.02279261
SEMA3F-AS1	ENSG00000235016	1.08858415	0.02294237
AC092651.2	ENSG00000266931	1.2313314	0.02308794
AL445931.1	ENSG00000235138	-1.44906260	0.02393889
KRT18P7	ENSG00000258951	2.03419163	0.02398025
BCL2L2	ENSG00000129473	2.10134403	0.02430837
KCNS3	ENSG00000170745	1.19469569	0.02440807
AGMO	ENSG00000187546	-1.36604862	0.0246899
AC109446.3	ENSG00000261448	-1.15733469	0.02504738
CLDN12	ENSG00000157224	1.00226416	0.02511726
STMN1P1	ENSG00000276058	2.38954086	0.02524759
AC011676.5	ENSG00000280035	-1.29935011	0.02533823
AC096921.2	ENSG00000261468	-1.80062489	0.02534169
AC008608.2	ENSG00000271737	-1.39977810	0.02548182
AC137834.2	ENSG00000276727	2.50290012	0.02553479
KPNA7	ENSG00000185467	1.70433672	0.02558657
AC141586.2	ENSG00000260176	1.16931172	0.02575066
AC026785.3	ENSG00000249199	-2.09178082	0.02586332
SETSIP	ENSG00000230667	1.33047388	0.02615526
AL137003.1	ENSG00000229931	1.35506188	0.0263447
CR769767.2	ENSG00000276692	1.12678616	0.02639106
RPS15AP36	ENSG00000213013	Inf	0.02657416
TREX2	ENSG00000183479	-1.20597041	0.02664916
HIPK4	ENSG00000160396	-1.32290205	0.02728453
ANXA2R	ENSG00000177721	-1.01023040	0.02732868
LINC01220	ENSG00000259687	-2.48936566	0.02759776
TRIB3	ENSG00000101255	1.19427173	0.02782643
AL591684.2	ENSG00000254929	1.13559837	0.027849
LDHD	ENSG00000166816	-1.02957730	0.0285874

AL139246.5	ENSG00000272449	-1.286850287	0.02876577
AC018695.6	ENSG00000275393	-2.991690864	0.02890545
RNF2P1	ENSG00000231381	-1.797349995	0.02902257
LCE3D	ENSG00000163202	2.17677961	0.02906766
AL049629.2	ENSG00000284969	1.27483654	0.02910265
AC135050.2	ENSG00000255439	Inf	0.029222
A4GNT	ENSG00000118017	-1.302917656	0.02924711
AL160269.1	ENSG00000285269	1.70521071	0.02955752
AP000695.2	ENSG00000233818	1.29271331	0.03044739
AC079949.2	ENSG00000278266	-1.038710218	0.03051282
AC125437.1	ENSG00000267655	1.67952382	0.03070739
LENG8-AS1	ENSG00000226696	-1.211019151	0.03071217
AC006273.1	ENSG00000272473	-1.445696696	0.03117688
C3orf52	ENSG00000114529	1.37266519	0.03123346
ZNF181	ENSG00000197841	1.09142301	0.03136375
TREX1	ENSG00000213689	-1.354826896	0.03138637
IPO9-AS1	ENSG00000231871	-1.824032911	0.03174771
SNORA28	ENSG00000272533	2.7433767	0.03222404
AC108134.2	ENSG00000261889	1.84842747	0.03237096
AL353801.3	ENSG00000273363	-3.534101081	0.03259737
FLVCR1-AS1	ENSG00000198468	-1.013174291	0.0326264
LIMS1	ENSG00000169756	1.20299902	0.03388206
MAST4-AS1	ENSG00000229666	-1.808528587	0.03390168
VGF	ENSG00000128564	1.24561573	0.03394944
AC080188.2	ENSG00000279384	2.1921836	0.03414353
AC007161.3	ENSG00000283549	Inf	0.03417959
MMP10	ENSG00000166670	1.88751179	0.03504043
FRMD8	ENSG00000126391	-1.093029150	0.03634849
LINC01474	ENSG00000236849	-1.702942618	0.03653957
PGAM1P8	ENSG00000255200	-2.102853496	0.03756949
RFPL4A	ENSG00000223638	1.83227324	0.03778359
AC053513.1	ENSG00000256973	-2.184759217	0.03787581
AC000093.1	ENSG00000284874	-2.142809325	0.03818545
AP001363.1	ENSG00000250659	Inf	0.03912027
AC079203.1	ENSG00000243094	2.94861974	0.03950531
AC013444.2	ENSG00000270390	-1.331837362	0.03975776
AC139149.1	ENSG00000229848	-1.230681161	0.03975942
COX20P1	ENSG00000213025	3.97224858	0.04009801
AC015971.1	ENSG00000228363	1.07612768	0.04037224
AP003119.2	ENSG00000255100	-1.514967764	0.04059897
C16orf46	ENSG00000166455	1.12505423	0.04100973
TACC1	ENSG00000147526	-1.183647969	0.04121469
AL713922.2	ENSG00000276662	2.74123723	0.04121587
PCBP2-OT1	ENSG00000282977	1.89234407	0.04170505
PPIAP54	ENSG00000264655	-2.682180585	0.0423599
AC107959.1	ENSG00000245025	1.07458727	0.0427463
AC092117.2	ENSG00000279901	1.38163602	0.04283202
DEFB1	ENSG00000164825	-1.497087312	0.04335303
AC025423.1	ENSG00000256325	2.18568339	0.04341308
AL132780.5	ENSG00000280129	-1.477134016	0.04362257
AC092120.1	ENSG00000261692	3.49585971	0.04363823
AC067931.1	ENSG00000279766	1.53397417	0.04364826
AC091133.4	ENSG00000251550	1.03935327	0.04411322
AC008895.1	ENSG00000279948	1.08595918	0.04440167
LINC02154	ENSG00000235385	2.86170011	0.0449321
NPIPB3	ENSG00000169246	1.04379052	0.04511112
AC012467.1	ENSG00000271916	-4.205101085	0.04512815
HRASLS2	ENSG00000133328	-1.933584659	0.04557629

RF00017	ENSG00000280502	-3.77311736	0.04559073
AC002467.1	ENSG00000241764	1.13413571	0.04571544
UBE2V1P1	ENSG00000226632	2.1569545	0.04609298
AC131009.4	ENSG00000279283	1.05702564	0.04705801
ZNF518A	ENSG00000177853	1.26341954	0.04734197
AL121832.3	ENSG00000275437	1.11102838	0.04747626
AP002990.1	ENSG00000255508	3.06437049	0.04749879
IFNL3P1	ENSG00000268510	-1.09269280	0.04751869
AC243964.2	ENSG00000266903	-1.92481972	0.04756019
AQP11	ENSG00000178301	-1.09535371	0.04763704
CTAGE7P	ENSG00000233122	-1.21019447	0.048235
AL359643.3	ENSG00000272142	-1.23977081	0.04836272
AC084880.1	ENSG00000213144	1.14795246	0.04858296
TMOD1	ENSG00000136842	1.00520279	0.04981848
MIR210HG	ENSG00000247095	-1.60315667	0.04986964

RNA-seq differentially genes (LINC01977-ASO vs Scramble)

Symbol	Ensembl	logFC	*P* value
CXCL1	ENSG00000163739	-3.464287166	1.152E-179
TM4SF20	ENSG00000168955	-3.392006299	8.911E-172
MUC5B	ENSG00000117983	-3.313487319	1.941E-162
STC2	ENSG00000113739	2.57539302	3.4835E-95
CDH1	ENSG00000039068	-2.424460139	5.7524E-93
PDK4	ENSG00000004799	-2.487610161	4.0583E-90
MEGF9	ENSG00000106780	-2.318407467	5.1098E-90
NEB	ENSG00000183091	-3.613709922	4.8287E-75
SPDEF	ENSG00000124664	-3.346754866	2.9155E-70
ERBB3	ENSG00000065361	-2.865636175	8.4286E-70
CHAC1	ENSG00000128965	3.38052255	7.1371E-69
HNF4A	ENSG00000101076	-2.587317433	3.8973E-68
FOSL1	ENSG00000175592	2.2136803	2.551E-67
SAMD11	ENSG00000187634	-2.834132598	3.2188E-67
MUC5AC	ENSG00000215182	-1.858170371	3.2195E-66
CP	ENSG00000047457	-1.935240769	1.9139E-64
DHCR24	ENSG00000116133	-1.918984249	1.7292E-63
SLC12A2	ENSG00000064651	-2.007562276	6.7174E-63
UBE2F-SCLY	ENSG00000258984	-6.153017766	8.982E-62
CFH	ENSG00000000971	-2.148569230	1.8898E-61
FCGBP	ENSG00000275395	-1.885873038	3.1205E-61
PTGES	ENSG00000148344	-1.944291219	2.4161E-58
GJA1	ENSG00000152661	-1.985767301	1.0778E-57
TUBB3	ENSG00000258947	2.16937078	1.7683E-57
NOTCH3	ENSG00000074181	-1.774448924	2.4452E-57
MTUS1	ENSG00000129422	-1.752443095	2.3634E-55
SMOC1	ENSG00000198732	-1.942045549	2.5058E-53
OSGIN1	ENSG00000140961	1.94721799	4.8085E-53
RHOV	ENSG00000104140	-2.680762899	7.9979E-53
TMOD3	ENSG00000138594	-1.668016438	1.252E-51
LAMC2	ENSG00000058085	1.79340501	1.4119E-51
TM4SF4	ENSG00000169903	-1.713960780	3.0661E-51
CXCL3	ENSG00000163734	-2.051770234	5.791E-51
THRA	ENSG00000126351	-1.945976473	1.651E-50
TIPARP	ENSG00000163659	1.79673353	4.1764E-50
CORO2A	ENSG00000106789	-2.706899613	5.9666E-49
SLC7A7	ENSG00000155465	-1.792790967	2.0177E-47
GPX2	ENSG00000176153	-1.694937760	3.4836E-47
NUDT15	ENSG00000136159	-2.273827337	7.6145E-47
THSD7A	ENSG00000005108	-1.890538344	9.5826E-47
HR	ENSG00000168453	-1.694772261	1.9703E-45
TNFRSF12A	ENSG00000006327	1.66662647	4.4042E-45
SLC23A2	ENSG00000089057	-1.562857699	6.8067E-45
ETS1	ENSG00000134954	1.68816787	1.9413E-44
USH1C	ENSG00000006611	-3.276817546	5.2627E-43
STK40	ENSG00000196182	1.94610832	8.3136E-43
NTS	ENSG00000133636	-1.719466941	9.4198E-43
SESN2	ENSG00000130766	1.796299	1.219E-42
MMGT1	ENSG00000169446	-1.840091098	4.423E-42
ARHGAP18	ENSG00000146376	-1.988088382	6.0858E-42
SH3PXD2A	ENSG00000107957	-1.929733769	1.1822E-41
CBX5	ENSG00000094916	-1.516559261	1.2443E-41
TRIB3	ENSG00000101255	1.62924989	6.1738E-41
VGLL3	ENSG00000206538	-2.952392522	7.9774E-41
RASSF9	ENSG00000198774	-2.355598491	1.0178E-40
ABCG1	ENSG00000160179	-2.129352852	1.6902E-40

DIXDC1	ENSG00000150764	-2.688155389	1.8257E-40
HSPA1B	ENSG00000204388	-1.469788069	4.0693E-40
TSC22D3	ENSG00000157514	2.06625375	7.0003E-40
HGD	ENSG00000113924	-2.597801297	1.3366E-39
FN1	ENSG00000115414	-1.469799203	1.6161E-39
SFN	ENSG00000175793	1.54464388	3.1857E-39
ANKS4B	ENSG00000175311	-3.270188879	6.4726E-39
C3	ENSG00000125730	-1.505767500	1.6609E-38
SLC23A1	ENSG00000170482	-2.231136421	1.9998E-38
ODC1	ENSG00000115758	1.50770651	2.0525E-38
ANXA13	ENSG00000104537	-2.319702054	2.6635E-38
NAV3	ENSG00000067798	1.81202598	2.758E-38
BRI3BP	ENSG00000184992	-1.523477525	3.1884E-38
SYNM	ENSG00000182253	-1.552642919	7.0369E-38
SMURF2	ENSG00000108854	1.61081489	1.2488E-37
KAT6A	ENSG00000083168	-1.614113452	1.3671E-37
SERPINE1	ENSG00000106366	1.46971158	1.4829E-37
HMGA2	ENSG00000149948	1.61627751	2.4499E-37
LANCL1	ENSG00000115365	-1.493179286	2.6566E-37
RAB8B	ENSG00000166128	-1.746897602	5.8796E-37
CDH17	ENSG00000079112	-1.668845946	6.2389E-37
SLC39A10	ENSG00000196950	-1.546834217	1.246E-36
EPS8	ENSG00000151491	-1.468366377	1.2937E-36
TTC39B	ENSG00000155158	-1.901928692	2.572E-36
TCIM	ENSG00000176907	-1.607513354	2.8916E-36
PDLIM5	ENSG00000163110	-1.448038288	4.0797E-36
TJP3	ENSG00000105289	-3.056372606	4.7818E-36
FGFR3	ENSG00000068078	-2.757853726	5.4947E-36
SUCNR1	ENSG00000198829	-2.993085871	6.2739E-36
PPIF	ENSG00000108179	1.51943216	2.0126E-35
THRB	ENSG00000151090	-2.232298876	2.6258E-35
MARCH4	ENSG00000144583	1.62425148	3.3018E-35
GREM1	ENSG00000166923	2.11051725	4.0776E-35
ICMT	ENSG00000116237	-1.520651323	8.4237E-35
VCAN	ENSG00000038427	-1.478344139	1.0685E-34
HSPA1A	ENSG00000204389	-1.353394430	1.4332E-34
C5	ENSG00000106804	-1.670987707	3.0381E-34
SORL1	ENSG00000137642	-1.875816325	3.6929E-34
ANXA4	ENSG00000196975	-1.335584378	7.3858E-34
CBFB	ENSG00000067955	-1.811333264	8.5289E-34
ZC3H12A	ENSG00000163874	-1.355576009	9.3455E-34
PTPRU	ENSG00000060656	-1.650341133	1.5448E-33
EGR1	ENSG00000120738	1.73084859	1.6669E-33
DOK4	ENSG00000125170	-1.520855149	1.907E-33
ADSSL1	ENSG00000185100	-2.673463329	2.1707E-33
PLEK2	ENSG00000100558	2.00472435	2.5749E-33
SNAP25	ENSG00000132639	-1.528857263	2.8212E-33
SLC45A4	ENSG00000022567	-1.362964330	3.1032E-33
RCOR1	ENSG00000089902	-1.438239178	3.9946E-33
CDKN2C	ENSG00000123080	-1.916067408	4.7352E-33
ALDH1A1	ENSG00000165092	-1.270420572	1.1578E-32
MTHFD2	ENSG00000065911	1.39175046	1.1821E-32
LUCAT1	ENSG00000248323	1.79407773	3.3875E-32
TGFBR1	ENSG00000106799	-1.407868636	3.7525E-32
CIDEC	ENSG00000187288	-3.502675799	6.1205E-32
HEXIM1	ENSG00000186834	-1.322695994	6.8118E-32
SEMA4G	ENSG00000095539	-1.776589079	2.7921E-31
VMA21	ENSG00000160131	-1.453385631	3.435E-31

PLD5	ENSG00000180287	-2.152852319	3.4943E-31
GPRIN3	ENSG00000185477	-1.299451732	5.5638E-31
ULK1	ENSG00000177169	1.36276013	6.4752E-31
KIF21B	ENSG00000116852	-1.445889643	8.9032E-31
ADSS	ENSG00000035687	-1.363905751	1.1043E-30
ASS1	ENSG00000130707	-1.491403510	1.3326E-30
CELSR2	ENSG00000143126	-1.555600661	1.7807E-30
SPTSSA	ENSG00000165389	-1.335142805	1.9516E-30
KIAA1551	ENSG00000174718	1.59186165	2.2202E-30
SLC40A1	ENSG00000138449	-1.942047646	2.4775E-30
JUP	ENSG00000173801	-1.260196897	2.591E-30
HSPA5	ENSG00000044574	1.2944362	3.127E-30
FMN1	ENSG00000248905	-1.565531881	4.5421E-30
ALOXE3	ENSG00000179148	2.95706474	4.9899E-30
ITGAV	ENSG00000138448	-1.246777842	6.105E-30
FRMD6	ENSG00000139926	1.6765015	7.71E-30
PLA2G12A	ENSG00000123739	-1.991607953	8.5292E-30
MYOCD	ENSG00000141052	-1.760675474	1.0079E-29
OAS1	ENSG00000089127	-1.483928890	1.31E-29
EPS8L3	ENSG00000198758	-3.001573307	1.9762E-29
GLS	ENSG00000115419	1.31539049	2.0909E-29
STX6	ENSG00000135823	-1.547615736	2.1997E-29
AP1S3	ENSG00000152056	-1.595866116	3.0873E-29
SDK2	ENSG00000069188	-2.220976672	3.2441E-29
TRIML2	ENSG00000179046	1.42612143	3.2596E-29
AP3M1	ENSG00000185009	-1.344420872	5.3754E-29
B4GALT5	ENSG00000158470	-1.264882919	5.8532E-29
APH1B	ENSG00000138613	-1.632507944	8.352E-29
ALDH3A2	ENSG00000072210	-1.292778682	1.0688E-28
LY6K	ENSG00000160886	2.99814901	1.3596E-28
F5	ENSG00000198734	-1.800904213	1.3946E-28
CDON	ENSG00000064309	-2.170243087	1.4414E-28
BCL2L11	ENSG00000153094	-1.578275642	2.5129E-28
HK2	ENSG00000159399	2.52073274	3.0015E-28
HERC5	ENSG00000138646	-1.280188261	3.8169E-28
SLC51B	ENSG00000186198	-2.930902710	4.1485E-28
CPS1	ENSG00000021826	-1.219002726	4.231E-28
SLPI	ENSG00000124107	-1.807126541	4.4021E-28
VAV3	ENSG00000134215	-1.943464641	4.7924E-28
NEDD4	ENSG00000069869	1.26225128	6.0207E-28
STARD7	ENSG00000084090	-1.254981427	1.0828E-27
OLFML2A	ENSG00000185585	-1.589238014	1.7702E-27
RNASE4	ENSG00000258818	-1.510589828	1.9515E-27
EHF	ENSG00000135373	-1.872778552	2.1436E-27
AC005077.4	ENSG00000230882	1.51913931	2.7501E-27
KLF6	ENSG00000067082	1.56497439	3.9382E-27
FGB	ENSG00000171564	-2.326842897	5.9431E-27
TSC22D2	ENSG00000196428	1.38823083	1.4383E-26
CD38	ENSG00000004468	-1.307160470	2.2046E-26
ADCY9	ENSG00000162104	-1.380676227	6.9857E-26
IGFBP1	ENSG00000146678	1.26840663	8.1393E-26
HACD3	ENSG00000074696	-1.170743473	8.7818E-26
PLPP3	ENSG00000162407	1.37353081	8.9612E-26
FGFR4	ENSG00000160867	-1.325187324	1.002E-25
FAM57A	ENSG00000167695	1.55069017	1.0596E-25
F2RL1	ENSG00000164251	1.24835832	1.1824E-25
SOX9	ENSG00000125398	-1.452259021	1.4057E-25
HLA-DMB	ENSG00000242574	-3.670753791	1.5126E-25

NTRK3	ENSG00000140538	-2.06478184[illegible]	1.6837E-25
CAMLG	ENSG00000164615	-1.51545947[illegible]	1.9213E-25
MMD	ENSG00000108960	-1.32198423[illegible]	1.9591E-25
RASAL2	ENSG00000075391	1.52151323	1.9889E-25
EVA1C	ENSG00000166979	-1.54649217[illegible]	4.4571E-25
KRT18	ENSG00000111057	-1.11229577[illegible]	4.592E-25
DPY19L1	ENSG00000173852	-1.15184180[illegible]	4.6759E-25
TNS1	ENSG00000079308	-1.66274427[illegible]	5.46E-25
KLHL21	ENSG00000162413	1.32522239	6.209E-25
GALNT4	ENSG00000257594	-1.27875965[illegible]	7.9126E-25
PLCH1	ENSG00000114805	-1.69492076[illegible]	1.1182E-24
CHSY1	ENSG00000131873	-1.41959533[illegible]	1.5884E-24
ASNS	ENSG00000070669	1.27040482	1.9438E-24
HAS2	ENSG00000170961	1.66027479	2.3083E-24
CYP24A1	ENSG00000019186	-1.09791434[illegible]	2.3563E-24
SMIM14	ENSG00000163683	-1.28028944[illegible]	2.6397E-24
ADGRB2	ENSG00000121753	-1.69870048[illegible]	3.3103E-24
MYORG	ENSG00000164976	-1.33615505[illegible]	5.7951E-24
GADD45A	ENSG00000116717	1.36847997	9.4027E-24
S100P	ENSG00000163993	-1.37310162[illegible]	1.0787E-23
LINC00473	ENSG00000223414	-1.39895464[illegible]	1.5416E-23
AL163636.2	ENSG00000259171	-3.09794205[illegible]	1.9494E-23
SLC17A3	ENSG00000124564	-2.71020982[illegible]	2.1469E-23
CAMK2N1	ENSG00000162545	-1.32166095[illegible]	2.3733E-23
PIK3AP1	ENSG00000155629	-1.89799958[illegible]	2.9311E-23
VIL1	ENSG00000127831	-4.89296158[illegible]	3.8991E-23
IL1RL1	ENSG00000115602	4.9225945	4.0708E-23
SLC22A18	ENSG00000110628	-1.35460131[illegible]	5.4633E-23
SLITRK5	ENSG00000165300	-2.19293017[illegible]	5.9766E-23
RHNO1	ENSG00000171792	-1.43106347[illegible]	6.3032E-23
ISG20L2	ENSG00000143319	1.30513176	6.7253E-23
JUN	ENSG00000177606	1.14080752	8.0097E-23
SORT1	ENSG00000134243	-1.52322705[illegible]	8.3968E-23
THOP1	ENSG00000172009	-1.30988614[illegible]	9.2454E-23
AP000439.2	ENSG00000255774	-2.18418776[illegible]	1.7295E-22
KCNJ16	ENSG00000153822	-2.42268261[illegible]	1.8114E-22
SNPH	ENSG00000101298	-1.42260777[illegible]	2.3962E-22
PCSK9	ENSG00000169174	-1.22001436[illegible]	2.4384E-22
EGFR	ENSG00000146648	1.09824544	2.9438E-22
RHOB	ENSG00000143878	1.12149127	3.1337E-22
TNPO1	ENSG00000083312	1.0954948	3.3117E-22
CBX6	ENSG00000183741	-1.14401488[illegible]	4.7423E-22
FZD4	ENSG00000174804	-1.30692913[illegible]	5.68E-22
IFNL2	ENSG00000183709	-1.49035353[illegible]	5.8728E-22
SLC44A1	ENSG00000070214	-1.09086991[illegible]	7.3238E-22
HAS3	ENSG00000103044	1.20523145	7.3965E-22
IFNL3	ENSG00000197110	-1.57034697[illegible]	8.0602E-22
NEO1	ENSG00000067141	-1.15316980[illegible]	9.4334E-22
OTUD1	ENSG00000165312	-1.30355577[illegible]	9.7485E-22
PDE3A	ENSG00000172572	-1.78173128[illegible]	9.8172E-22
LIN7A	ENSG00000111052	-1.34161649[illegible]	1.0292E-21
GOLGA4	ENSG00000144674	1.16345235	1.0895E-21
SLC7A1	ENSG00000139514	1.11594889	1.3385E-21
ARHGEF40	ENSG00000165801	-1.35702815[illegible]	1.4665E-21
ZNF367	ENSG00000165244	1.35367146	1.613E-21
UPK3B	ENSG00000243566	-1.75773534[illegible]	1.7775E-21
PLEKHG2	ENSG00000090924	-1.18552534[illegible]	2.0352E-21
GOLT1B	ENSG00000111711	-1.18449431[illegible]	2.3365E-21

AP003119.3	ENSG00000261578	-1.51569978	2.7919E-21
RAB37	ENSG00000172794	-1.78391543	3.134E-21
ATF4	ENSG00000128272	1.08668597	3.6818E-21
DDIT4L	ENSG00000145358	-1.87359206	3.7118E-21
LRFN1	ENSG00000128011	-1.37404180	3.7383E-21
TMEM37	ENSG00000171227	-2.46638723	4.1171E-21
PGM2L1	ENSG00000165434	-1.25546141	5.37E-21
MZT1	ENSG00000204899	-1.34901071	5.856E-21
UPP1	ENSG00000183696	1.15309079	5.9603E-21
BCAM	ENSG00000187244	-1.42548386	6.0181E-21
RAB27B	ENSG00000041353	-1.04731041	6.6075E-21
NRG1	ENSG00000157168	1.11517563	7.7447E-21
UGDH	ENSG00000109814	-1.01583166	1.0437E-20
FAM129A	ENSG00000135842	1.27708821	1.0567E-20
AMIGO3	ENSG00000176020	-3.33884603	1.2195E-20
CMTM6	ENSG00000091317	-1.10444308	1.2635E-20
WRB	ENSG00000182093	-1.54463828	1.2824E-20
SMAD6	ENSG00000137834	-1.14682475	1.3136E-20
SCP2	ENSG00000116171	-1.18820496	1.3658E-20
MIR22HG	ENSG00000186594	1.70798643	1.722E-20
MIB1	ENSG00000101752	-1.11868171	1.7749E-20
SYT17	ENSG00000103528	-1.47095663	2.0982E-20
KCNV1	ENSG00000164794	-1.39069341	2.2479E-20
GABRE	ENSG00000102287	-1.09524610	2.2969E-20
ALDH2	ENSG00000111275	-1.03856459	2.4555E-20
GLI1	ENSG00000111087	-1.64516534	2.4834E-20
LINC02582	ENSG00000261780	-1.23720738	2.5641E-20
DCLK1	ENSG00000133083	1.98376867	2.6037E-20
CTPS1	ENSG00000171793	1.08719552	2.6893E-20
RHPN2	ENSG00000131941	-1.08068725	2.7496E-20
CGN	ENSG00000143375	-1.89494878	3.0563E-20
OAS2	ENSG00000111335	-1.53960799	3.1256E-20
SEMA3B	ENSG00000012171	-1.52452109	3.2251E-20
FLRT3	ENSG00000125848	-1.51202494	4.4641E-20
CACNA1G	ENSG00000006283	-1.50457913	5.4681E-20
IFNL1	ENSG00000182393	-1.05046890	6.7219E-20
CNTN1	ENSG00000018236	-1.13729612	8.3499E-20
COL4A4	ENSG00000081052	-1.31065972	8.3712E-20
RBFOX3	ENSG00000167281	-2.08350040	8.831E-20
IGFBP3	ENSG00000146674	1.05994951	9.9563E-20
C11orf68	ENSG00000175573	1.26905821	9.967E-20
UGT1A7	ENSG00000244122	-1.52027610	1.2356E-19
IPMK	ENSG00000151151	-1.25378326	1.3837E-19
FAM102A	ENSG00000167106	1.05934658	1.4897E-19
MYADM	ENSG00000179820	1.04095346	1.5438E-19
PLSCR4	ENSG00000114698	-1.80236351	1.569E-19
TANC1	ENSG00000115183	-1.20210505	1.5726E-19
SERINC5	ENSG00000164300	-1.22248353	1.5759E-19
LIMK1	ENSG00000106683	1.16649425	1.6733E-19
MYO1A	ENSG00000166866	-3.88110144	1.7385E-19
CSF1	ENSG00000184371	-1.12523100	1.9388E-19
MSANTD3	ENSG00000066697	1.12198978	2.1004E-19
PON3	ENSG00000105852	-1.32842030	2.1207E-19
GTF2IP4	ENSG00000233369	-1.04474020	2.1725E-19
ARL6IP5	ENSG00000144746	-1.08471480	2.2877E-19
CXXC4	ENSG00000168772	-2.72055701	2.4593E-19
GMFB	ENSG00000197045	-1.06100872	2.4929E-19
SHROOM3	ENSG00000138771	-1.64170564	2.7818E-19

BRCA2	ENSG00000139618	1.37441268	2.8085E-19
TMED2	ENSG00000086598	-1.02730824(	2.9248E-19
SMO	ENSG00000128602	-1.37086588	3.216E-19
PLEKHM1	ENSG00000225190	1.14495107	3.7633E-19
TUBB4A	ENSG00000104833	-1.58451064	4.3138E-19
CA11	ENSG00000063180	-1.82209628(	4.6506E-19
HMGCS1	ENSG00000112972	1.26626301	4.6936E-19
PAPSS2	ENSG00000198682	-1.08034784	5.3576E-19
BCHE	ENSG00000114200	-1.32405822(	6.4258E-19
NOL4L	ENSG00000197183	-1.32397677	8.8501E-19
TNS2	ENSG00000111077	-1.32966581	1.0767E-18
NR4A2	ENSG00000153234	-1.42561781	1.0783E-18
PPARGC1A	ENSG00000109819	-2.31688248	1.1634E-18
CMPK1	ENSG00000162368	-1.01173819	1.1709E-18
AFAP1	ENSG00000196526	-1.05191022	1.1946E-18
MYO18A	ENSG00000196535	-1.00430428	1.1996E-18
FBXW11	ENSG00000072803	-1.06254371	1.2468E-18
RRP12	ENSG00000052749	1.05331584	1.3262E-18
KIF12	ENSG00000136883	-1.99442766	1.6206E-18
SLC44A2	ENSG00000129353	-1.60289454	1.719E-18
TWNK	ENSG00000107815	-1.16284639	1.9981E-18
NAB1	ENSG00000138386	-1.04842318	2.3201E-18
TUFT1	ENSG00000143367	1.21795099	2.3247E-18
SUSD2	ENSG00000099994	-1.43096840	3.0355E-18
FGL1	ENSG00000104760	-1.86864853(	3.3829E-18
CSGALNACT2	ENSG00000169826	1.08627667	3.904E-18
SOWAHC	ENSG00000198142	1.21162295	4.3043E-18
THBD	ENSG00000178726	1.41273034	4.3119E-18
BTBD11	ENSG00000151136	-1.17213462	4.5716E-18
KCTD5	ENSG00000167977	1.13253163	5.2359E-18
GARS	ENSG00000106105	1.00080545	5.7143E-18
CLCF1	ENSG00000175505	1.06559634	6.6438E-18
FAM83A	ENSG00000147689	-2.00255580	6.7614E-18
VEGFA	ENSG00000112715	1.06407175	7.0606E-18
IDE	ENSG00000119912	-1.01665404	7.3763E-18
BDNF	ENSG00000176697	-2.09593803(	7.7829E-18
ARRB1	ENSG00000137486	-1.51011217	7.7883E-18
ADRA1D	ENSG00000171873	-2.78365543	8.3684E-18
CAPN15	ENSG00000103326	1.04004796	9.3686E-18
EPDR1	ENSG00000086289	-1.01422370	2.3701E-17
CTNNAL1	ENSG00000119326	1.04974859	2.5403E-17
SLC6A9	ENSG00000196517	1.36519889	2.671E-17
DNM1	ENSG00000106976	-1.05866874	2.6854E-17
CPOX	ENSG00000080819	-1.19116793	2.8555E-17
CHMP1A	ENSG00000131165	1.05521561	3.4825E-17
PARM1	ENSG00000169116	-1.95716019	4.1166E-17
SERTAD1	ENSG00000197019	1.1973572	4.8533E-17
C1GALT1C1	ENSG00000171155	-1.31858050	5.4305E-17
SLC9A3R2	ENSG00000065054	-1.10316288	8.9329E-17
ABCA1	ENSG00000165029	-1.00592836(	1.1182E-16
MEAF6	ENSG00000163875	-1.25429165	1.2844E-16
CYB5A	ENSG00000166347	-1.24033139	1.3116E-16
IL6R	ENSG00000160712	2.09118294	1.6239E-16
SPOCK1	ENSG00000152377	1.61744452	1.6258E-16
VASH2	ENSG00000143494	-2.55169495	1.6883E-16
FILIP1	ENSG00000118407	-1.31505773	2.0309E-16
FLCN	ENSG00000154803	1.01748706	2.343E-16
MGAM	ENSG00000257335	-1.68762064	2.4623E-16

CFB	ENSG00000243649	-1.007984439	2.6852E-16
AC233724.15	ENSG00000283740	-3.284020650	3.0303E-16
CH25H	ENSG00000138135	-1.649857114	3.071E-16
ADD3	ENSG00000148700	-1.773836675	3.5061E-16
NABP1	ENSG00000173559	1.12010277	3.7389E-16
DAGLB	ENSG00000164535	1.16378284	4.6539E-16
ZBTB32	ENSG00000011590	-1.829801079	5.3422E-16
PROS1	ENSG00000184500	-1.099501379	5.7509E-16
LBP	ENSG00000129988	-4.829440960	6.3705E-16
NPY4R2	ENSG00000264717	-1.707985999	6.9155E-16
CEP135	ENSG00000174799	1.25246062	7.3846E-16
PIGK	ENSG00000142892	-1.137758943	7.5498E-16
FAM46A	ENSG00000112773	-1.245295880	8.2231E-16
DCK	ENSG00000156136	-1.143738408	8.3461E-16
DHTKD1	ENSG00000181192	-1.098204771	8.9138E-16
AKNA	ENSG00000106948	1.27274051	9.3029E-16
NCOA2	ENSG00000140396	-1.024586363	9.3676E-16
GMCL1	ENSG00000087338	-1.097436283	1.0021E-15
PLA2G4A	ENSG00000116711	-1.081905447	1.1028E-15
FCHSD2	ENSG00000137478	-1.176095477	1.1409E-15
SUZ12	ENSG00000178691	-1.002295429	1.4714E-15
JAKMIP3	ENSG00000188385	-1.191367264	1.4833E-15
AL645608.1	ENSG00000223764	-2.330925551	1.5288E-15
PAX7	ENSG00000009709	-1.143250306	1.854E-15
SMAP2	ENSG00000084070	-1.561957623	1.9711E-15
SELENBP1	ENSG00000143416	-2.901929703	2.1545E-15
EFNA1	ENSG00000169242	-1.106140057	2.5142E-15
SEMA4D	ENSG00000187764	-1.259138216	2.5718E-15
MED10	ENSG00000133398	1.02746932	2.7425E-15
ANG	ENSG00000214274	-1.507472910	2.8122E-15
PLLP	ENSG00000102934	-1.459626009	2.8649E-15
EEA1	ENSG00000102189	1.22519433	2.9725E-15
DOC2B	ENSG00000272636	-2.748662162	2.9858E-15
APLP1	ENSG00000105290	-1.006748243	3.4766E-15
NPY1R	ENSG00000164128	-3.266758336	3.5798E-15
CASTOR3	ENSG00000239521	-1.384850069	4.1802E-15
EBF4	ENSG00000088881	-1.751563878	4.665E-15
AMPD3	ENSG00000133805	2.39581215	4.8422E-15
FOXA1	ENSG00000129514	-1.526327384	5.6474E-15
RAVER1	ENSG00000161847	1.1156526	5.7442E-15
PIGW	ENSG00000277161	1.0417443	6.1906E-15
NDUFA5	ENSG00000128609	-1.236360134	6.2289E-15
LINC01963	ENSG00000260804	-1.350486236	6.4197E-15
NPY4R	ENSG00000204174	-1.500019630	6.7919E-15
FAM69A	ENSG00000154511	-1.151774822	7.2617E-15
ENTPD7	ENSG00000198018	1.22313179	8.4347E-15
PIM2	ENSG00000102096	-1.705772148	9.8556E-15
ACSF2	ENSG00000167107	-1.492620511	1.0416E-14
NR2F1	ENSG00000175745	-1.792188875	1.222E-14
MEIOB	ENSG00000162039	1.49053787	1.4126E-14
HHIP	ENSG00000164161	-2.177119430	1.6137E-14
ZMYM3	ENSG00000147130	-1.012986348	1.7764E-14
ALDH1A3	ENSG00000184254	1.10754309	1.8237E-14
PIK3C2B	ENSG00000133056	-1.001496032	1.9517E-14
SERPINI1	ENSG00000163536	-2.400549960	1.973E-14
UBA3	ENSG00000144744	-1.029916952	2.0737E-14
CABLES2	ENSG00000149679	-1.437249759	2.1149E-14
DHRS3	ENSG00000162496	-1.513672849	2.717E-14

IFNB1	ENSG00000171855	-1.427912085	2.8969E-14
ITGA1	ENSG00000213949	-1.306809488	3.2031E-14
UBE2L6	ENSG00000156587	-1.131973712	3.2315E-14
HSPA4L	ENSG00000164070	-1.023429669	3.5057E-14
ZNF467	ENSG00000181444	-3.007498282	3.6515E-14
RAB3D	ENSG00000105514	-1.064060380	3.7528E-14
IER3IP1	ENSG00000134049	-1.048026939	3.8471E-14
KLHL23	ENSG00000213160	-1.133856196	4.483E-14
RFC3	ENSG00000133119	-1.039396293	4.5529E-14
SEMA7A	ENSG00000138623	2.0159616	4.8944E-14
ITGB6	ENSG00000115221	-1.649164405	5.1225E-14
FAM19A5	ENSG00000219438	-1.878154276	5.1746E-14
WNT9A	ENSG00000143816	1.45950907	6.0746E-14
LCLAT1	ENSG00000172954	-1.002321617	6.7242E-14
CYR61	ENSG00000142871	1.1249925	7.0989E-14
PHACTR2	ENSG00000112419	-1.063062048	7.4507E-14
MFSD2A	ENSG00000168389	-1.507797450	7.6972E-14
DOCK8	ENSG00000107099	-1.467676904	7.7497E-14
TUB	ENSG00000166402	-1.145850827	8.0411E-14
CTSF	ENSG00000174080	-1.212595434	8.365E-14
CDH4	ENSG00000179242	1.56573286	8.4011E-14
TST	ENSG00000128311	-1.011243931	8.8115E-14
OXTR	ENSG00000180914	1.2341333	1.0257E-13
DDIT3	ENSG00000175197	1.76927694	1.0345E-13
IBA57	ENSG00000181873	1.28342092	1.0605E-13
SYT6	ENSG00000134207	-1.392628115	1.12E-13
TRIM2	ENSG00000109654	-1.953563359	1.1576E-13
TMCC1	ENSG00000172765	1.02916913	1.2437E-13
RTL9	ENSG00000243978	-1.695894284	1.336E-13
DUSP7	ENSG00000164086	1.27866092	1.3461E-13
SULT2B1	ENSG00000088002	-1.869611030	1.6696E-13
ZNF704	ENSG00000164684	-1.196889562	1.7606E-13
DNAJB2	ENSG00000135924	1.00103266	1.771E-13
CACNA1D	ENSG00000157388	-1.148620212	1.9293E-13
CD163L1	ENSG00000177675	2.7098134	2.0508E-13
EPC2	ENSG00000135999	1.05180337	2.1701E-13
TFDP2	ENSG00000114126	-1.000170755	2.5181E-13
EXPH5	ENSG00000110723	1.47461925	2.5199E-13
BICDL1	ENSG00000135127	-1.255226869	2.5259E-13
C10orf91	ENSG00000180066	-1.775368900	2.6768E-13
PDK3	ENSG00000067992	-1.291127807	2.9351E-13
RAB29	ENSG00000117280	-1.103774420	3.0305E-13
OPHN1	ENSG00000079482	-1.224816553	3.0612E-13
PNPO	ENSG00000108439	-1.048752858	3.116E-13
PRNP	ENSG00000171867	1.88735544	3.2402E-13
AC004656.1	ENSG00000260822	-1.239968510	3.4436E-13
GRB7	ENSG00000141738	-1.455979961	3.7324E-13
TTC33	ENSG00000113638	-1.231530204	4.0231E-13
SP2	ENSG00000167182	1.04268823	4.5544E-13
COPS7A	ENSG00000111652	-1.002817406	4.7575E-13
MYRF	ENSG00000124920	-1.104389690	4.9251E-13
PLEKHA4	ENSG00000105559	-1.128357821	5.8394E-13
FRK	ENSG00000111816	-1.089379884	6.0924E-13
SAV1	ENSG00000151748	-1.012990646	6.4074E-13
FBXL17	ENSG00000145743	-1.221945482	6.4243E-13
SLFNL1-AS1	ENSG00000281207	1.65846537	6.9482E-13
VTN	ENSG00000109072	-1.580473276	1.0329E-12
CEBPA	ENSG00000245848	-1.360374491	1.0448E-12

STRADB	ENSG00000082146	-1.24680464[illegible]	1.1442E-12
LRIG1	ENSG00000144749	-1.21494258[illegible]	1.2427E-12
SEMA6B	ENSG00000167680	-1.28126134[illegible]	1.2784E-12
F7	ENSG00000057593	-2.59244749[illegible]	1.4694E-12
ADPRHL2	ENSG00000116863	1.06289591	1.4957E-12
KSR2	ENSG00000171435	1.46852634	1.5403E-12
LRRC8B	ENSG00000197147	-1.08783720[illegible]	1.5807E-12
ZNF107	ENSG00000196247	1.37167144	1.6254E-12
ZNF185	ENSG00000147394	1.26408032	1.8904E-12
AC007325.2	ENSG00000277196	-2.28559519[illegible]	2.0289E-12
LIPA	ENSG00000107798	-1.73260555[illegible]	2.2104E-12
RTN4R	ENSG00000040608	1.68278172	2.4083E-12
L3MBTL3	ENSG00000198945	-1.61060811[illegible]	2.4514E-12
LHPP	ENSG00000107902	-1.36217787[illegible]	2.6357E-12
ELOVL3	ENSG00000119915	-2.47244029[illegible]	2.6396E-12
TC2N	ENSG00000165929	-1.49527495[illegible]	2.7997E-12
TMEM164	ENSG00000157600	-1.82828449[illegible]	3.3342E-12
GPR20	ENSG00000204882	-2.16749956[illegible]	3.3698E-12
USP18	ENSG00000184979	-1.17014872[illegible]	3.4E-12
C3orf58	ENSG00000181744	-1.46764274[illegible]	3.7274E-12
ING1	ENSG00000153487	1.20614002	4.2174E-12
PTP4A3	ENSG00000184489	-1.23784351[illegible]	4.3492E-12
ADAM23	ENSG00000114948	-1.04633826[illegible]	6.1776E-12
RN7SK	ENSG00000283293	1.49530442	6.3453E-12
GYG2	ENSG00000056998	-1.80927270[illegible]	7.0973E-12
HHEX	ENSG00000152804	-1.12658035[illegible]	7.6254E-12
MAPRE3	ENSG00000084764	-1.16720125[illegible]	8.4511E-12
CDC25A	ENSG00000164045	1.06812469	1.1914E-11
PELI2	ENSG00000139946	-1.56016905[illegible]	1.3908E-11
PRR15	ENSG00000176532	-1.23828227[illegible]	1.4469E-11
STAG3	ENSG00000066923	-1.37263449[illegible]	1.5478E-11
TRIM31	ENSG00000204616	-3.29940185[illegible]	1.9221E-11
UNC13A	ENSG00000130477	-1.24884129[illegible]	1.9367E-11
RGS20	ENSG00000147509	1.19308099	2.1192E-11
EHD3	ENSG00000013016	-1.18844237[illegible]	2.1478E-11
METTL7B	ENSG00000170439	-1.41997656[illegible]	2.2147E-11
PDGFRL	ENSG00000104213	-1.64029420[illegible]	2.3558E-11
GPR35	ENSG00000178623	-2.34071026[illegible]	2.3861E-11
ADM2	ENSG00000128165	1.47996372	2.4319E-11
MVB12A	ENSG00000141971	-1.05093104[illegible]	3.3644E-11
CES1	ENSG00000198848	-1.04302508[illegible]	3.3959E-11
SLC16A4	ENSG00000168679	-1.11849701[illegible]	3.9886E-11
PADI1	ENSG00000142623	2.58949341	4.0023E-11
NNMT	ENSG00000166741	-1.20358683[illegible]	4.2245E-11
ATP4A	ENSG00000105675	-1.63752713[illegible]	4.3128E-11
MED12L	ENSG00000144893	-1.66752063[illegible]	4.3909E-11
TLE2	ENSG00000065717	-1.71912671[illegible]	4.5368E-11
SH2D5	ENSG00000189410	1.83872572	4.8406E-11
GPR37	ENSG00000170775	-1.67958110[illegible]	6.0318E-11
HADH	ENSG00000138796	-1.11641459[illegible]	6.0929E-11
ADAMTS10	ENSG00000142303	-1.19218374[illegible]	6.1599E-11
ZNF516	ENSG00000101493	-1.32023395[illegible]	6.362E-11
ATF5	ENSG00000169136	1.04370658	6.6197E-11
HRH2	ENSG00000113749	-2.40918173[illegible]	6.9111E-11
BMP4	ENSG00000125378	-2.18154911[illegible]	8.0692E-11
RAB20	ENSG00000139832	-1.89894725[illegible]	9.6196E-11
CTGF	ENSG00000118523	1.12007332	9.9536E-11
C8orf58	ENSG00000241852	1.24278651	1.0297E-10

GLIPR1	ENSG00000139278	1.05087522	1.4531E-10
SLC4A4	ENSG00000080493	-1.91555160	1.481E-10
CGB8	ENSG00000213030	3.57068987	1.5541E-10
DNAH17	ENSG00000187775	1.25488913	1.6187E-10
LRRC4B	ENSG00000131409	-2.63474037	1.9397E-10
HNF1A-AS1	ENSG00000241388	-2.54988495	1.9933E-10
BARD1	ENSG00000138376	-1.16403610	2.02E-10
CCDC92	ENSG00000119242	1.03187839	2.0957E-10
SLC7A2	ENSG00000003989	-1.38190299	2.1154E-10
SLC25A29	ENSG00000197119	-1.10211218	2.2455E-10
RHOU	ENSG00000116574	-1.16031948	2.3236E-10
C1orf115	ENSG00000162817	-1.12812744	2.3852E-10
LIMA1	ENSG00000050405	1.12964045	2.4253E-10
MAP3K12	ENSG00000139625	-1.00428898	2.7791E-10
RBPMS2	ENSG00000166831	-2.30293422	2.9468E-10
LRP4	ENSG00000134569	-1.07106233	3.1543E-10
SEMA3A	ENSG00000075213	-1.10832004	3.707E-10
ST8SIA4	ENSG00000113532	-1.48483210	4.5479E-10
PDE8B	ENSG00000113231	-1.26530350	4.6341E-10
SLC27A2	ENSG00000140284	-1.17480177	5.855E-10
ALDH5A1	ENSG00000112294	-1.12667442	6.2629E-10
HYAL4	ENSG00000106302	-3.45613234	6.3159E-10
AC092118.1	ENSG00000187185	-1.25578894	6.5562E-10
BMP8B	ENSG00000116985	-1.11660724	7.4005E-10
AP000769.1	ENSG00000173727	-1.59559642	7.475E-10
SCD5	ENSG00000145284	-1.18056246	8.751E-10
SH2B2	ENSG00000160999	-1.70294417	9.1411E-10
NDRG2	ENSG00000165795	-1.86727398	9.3551E-10
RBM38	ENSG00000132819	1.36006298	1.1341E-09
HUNK	ENSG00000142149	-1.58286401	1.1818E-09
FAM102B	ENSG00000162636	-1.28957584	1.3212E-09
ZNF703	ENSG00000183779	-1.71340815	1.351E-09
AC023157.3	ENSG00000276900	1.77119994	1.4234E-09
ZNF75A	ENSG00000162086	1.10996664	1.4973E-09
CACNG6	ENSG00000130433	-1.06172578	1.5115E-09
KRT17	ENSG00000128422	-1.32685062	1.6534E-09
HS3ST3B1	ENSG00000125430	-2.12802366	1.7699E-09
FBLN5	ENSG00000140092	-1.88854455	2.0653E-09
HMGCR	ENSG00000113161	1.04039212	2.089E-09
FGG	ENSG00000171557	-2.28040821	2.1819E-09
SYT12	ENSG00000173227	-1.28592894	2.1992E-09
EOGT	ENSG00000163378	1.0882206	2.2474E-09
PRG4	ENSG00000116690	-1.79613570	2.2795E-09
SIX4	ENSG00000100625	-1.05438132	2.3006E-09
BCL6	ENSG00000113916	-1.31286634	2.3777E-09
RBM20	ENSG00000203867	-1.04945855	2.6314E-09
AOX1	ENSG00000138356	1.16562593	2.6398E-09
CACNA1H	ENSG00000196557	-1.14539884	2.6726E-09
RARRES3	ENSG00000133321	-1.20262533	2.7386E-09
VSTM2L	ENSG00000132821	-2.58694879	3.0331E-09
MERTK	ENSG00000153208	-1.82592222	3.1151E-09
AC010531.1	ENSG00000131152	Inf	3.1622E-09
ANKRD1	ENSG00000148677	2.1143223	3.2084E-09
KCNH1	ENSG00000143473	1.5704407	3.2205E-09
RIN1	ENSG00000174791	1.00445161	3.4729E-09
C1QTNF6	ENSG00000133466	-1.17700488	3.5049E-09
SP6	ENSG00000189120	1.47841741	3.546E-09
VANGL2	ENSG00000162738	-1.27248997	3.691E-09

SLC29A4	ENSG00000164638	-1.20524347(	3.73E-09
SYNGR1	ENSG00000100321	-1.17013007(	4.6243E-09
PEAR1	ENSG00000187800	1.88055864	5.1623E-09
FBXL12	ENSG00000127452	1.05948302	5.9369E-09
RHBDL3	ENSG00000141314	-2.02688052;	6.3441E-09
MGST2	ENSG00000085871	-1.07689069;	7.9906E-09
CPM	ENSG00000135678	1.09194813	8.0216E-09
CAMSAP3	ENSG00000076826	-1.43242163:	8.3105E-09
AP1M2	ENSG00000129354	-1.28453312;	8.3599E-09
GTF2IP7	ENSG00000227038	1.47823092	8.5396E-09
KLF5	ENSG00000102554	-1.37721729;	8.5874E-09
ETV1	ENSG00000006468	-1.13012396!	8.6106E-09
FRMD3	ENSG00000172159	-1.13658581:	8.6343E-09
KRT15	ENSG00000171346	1.90095673	8.8875E-09
DTX2P1-UPK:	ENSG00000265479	-1.22900491;	9.026E-09
SGPP2	ENSG00000163082	-1.13954939;	9.2346E-09
SHISAL1	ENSG00000138944	1.27715803	1.0333E-08
AC015813.6	ENSG00000279207	-1.13362147;	1.0389E-08
ARAP3	ENSG00000120318	-1.02305900:	1.0767E-08
P2RY6	ENSG00000171631	-1.12396053(	1.1189E-08
TMEM158	ENSG00000249992	1.37953862	1.1386E-08
RHPN1	ENSG00000158106	-1.00793708!	1.19E-08
ST6GAL1	ENSG00000073849	-1.12110645;	1.2118E-08
BCAS1	ENSG00000064787	-1.67625018!	1.2159E-08
SNTB1	ENSG00000172164	-1.09130784(	1.3115E-08
SH3BGRL2	ENSG00000198478	-1.17285446:	1.4864E-08
BMPR1B	ENSG00000138696	-1.28671795;	1.5032E-08
POC1B-GALN	ENSG00000259075	-6.23668391!	1.5277E-08
AP001972.5	ENSG00000279117	-1.40197216!	1.6882E-08
MARCH9	ENSG00000139266	-1.11946710:	1.7384E-08
FNIP2	ENSG00000052795	-1.08488137!	1.9285E-08
FYN	ENSG00000010810	-1.27671321!	2.0071E-08
SLFNL1	ENSG00000171790	1.54010862	2.1807E-08
PXK	ENSG00000168297	-1.29024575;	2.184E-08
CHST1	ENSG00000175264	2.80968218	2.257E-08
C9orf152	ENSG00000188959	-2.53944512!	2.3376E-08
DGCR6	ENSG00000183628	-1.21399747:	2.4389E-08
MIR29B2CHG	ENSG00000203709	-1.11029199!	2.7991E-08
AC016831.7	ENSG00000285106	-1.87642042;	2.8483E-08
PARS2	ENSG00000162396	-1.33673571(	3.0043E-08
GSTM4	ENSG00000168765	-1.54459616:	3.1281E-08
MEX3A	ENSG00000254726	-1.28280793!	3.1544E-08
CYP4F12	ENSG00000186204	-1.34616659:	3.1897E-08
AC007325.4	ENSG00000278817	-1.11691398;	3.6631E-08
GNAZ	ENSG00000128266	-1.39346507!	3.8847E-08
FOSB	ENSG00000125740	1.57612429	3.9165E-08
SAP30L	ENSG00000164576	-1.42863010;	4.4134E-08
EHHADH	ENSG00000113790	-1.13086117;	5.2156E-08
DIO2	ENSG00000211448	1.40009656	5.3491E-08
PELI1	ENSG00000197329	-1.12591512:	5.3612E-08
ATXN7L2	ENSG00000162650	1.92814753	5.3669E-08
HSPA6	ENSG00000173110	1.91533306	6.0493E-08
CTNNBIP1	ENSG00000178585	-1.17508293;	6.1217E-08
CCDC106	ENSG00000173581	-1.00495519!	6.3693E-08
SATB2	ENSG00000119042	-1.01602853!	6.8541E-08
TMEM170B	ENSG00000205269	-1.34352184!	7.1762E-08
ONECUT2	ENSG00000119547	-1.12974899;	7.2348E-08
TBKBP1	ENSG00000198933	-1.14944139(	7.3232E-08

JAZF1	ENSG00000153814	-1.016876184	7.6535E-08
IPO4	ENSG00000196497	1.01978913	7.6846E-08
NKAIN4	ENSG00000101198	-1.989240447	8.7159E-08
SRPX2	ENSG00000102359	-1.105408737	9.993E-08
INSL4	ENSG00000120211	-1.027773933	1.0694E-07
AK7	ENSG00000140057	-1.431270248	1.0815E-07
RGS9	ENSG00000108370	-1.067076707	1.1455E-07
SEMA3D	ENSG00000153993	-1.397963539	1.211E-07
AC010997.3	ENSG00000270087	-7.106039714	1.211E-07
AC012513.3	ENSG00000279348	-1.226266338	1.2254E-07
HHIP-AS1	ENSG00000248890	-2.228235158	1.3065E-07
ENO1P1	ENSG00000244457	-1.716787183	1.3118E-07
HNMT	ENSG00000150540	-1.220154176	1.3747E-07
FJX1	ENSG00000179431	1.02206062	1.389E-07
FMO5	ENSG00000131781	-1.862198457	1.4488E-07
ISY1-RAB43	ENSG00000261796	Inf	1.456E-07
AC008440.3	ENSG00000232324	1.25961539	1.4685E-07
AL583856.1	ENSG00000232389	1.33177295	1.482E-07
PLEKHH2	ENSG00000152527	-1.382399431	1.5717E-07
AL133352.1	ENSG00000255339	-1.376059985	1.5947E-07
ZNF862	ENSG00000106479	-1.132440729	1.6475E-07
CASTOR2	ENSG00000274070	-1.352158534	1.6751E-07
TEAD3	ENSG00000007866	-1.045472265	1.85E-07
DPCD	ENSG00000166171	-1.305189447	1.9506E-07
FAM234B	ENSG00000084444	-1.103705252	2.2181E-07
NID1	ENSG00000116962	-1.012796839	2.2983E-07
POU2F1	ENSG00000143190	-1.354021738	2.5684E-07
OLAH	ENSG00000152463	2.28348109	2.7531E-07
ENTPD2	ENSG00000054179	-2.362635146	2.7623E-07
ZBTB43	ENSG00000169155	1.08480842	2.9325E-07
PPFIA3	ENSG00000177380	-1.118859058	2.9782E-07
BCORL1	ENSG00000085185	-1.012187606	3.4163E-07
KCNH3	ENSG00000135519	-1.664367700	3.5299E-07
ALB	ENSG00000163631	2.85780566	3.6068E-07
SPNS2	ENSG00000183018	1.37066695	3.6692E-07
SMARCD3	ENSG00000082014	-1.214828192	3.6918E-07
ABCC6	ENSG00000091262	-1.776527500	3.7741E-07
DTX4	ENSG00000110042	-1.989157495	3.8212E-07
GALNT9	ENSG00000182870	2.1743885	3.938E-07
TM7SF2	ENSG00000149809	-1.112621580	3.9829E-07
ICAM5	ENSG00000105376	-1.972431025	4.0959E-07
GRTP1	ENSG00000139835	-1.779956309	4.7101E-07
LINC01583	ENSG00000259518	3.45129196	4.8001E-07
N6AMT1	ENSG00000156239	-1.010342712	4.8455E-07
PHF21A	ENSG00000135365	-1.004932866	4.9968E-07
HNF1A	ENSG00000135100	-1.465771467	5.2328E-07
KRT8	ENSG00000170421	-1.000995402	5.2911E-07
CD302	ENSG00000241399	-1.349307812	5.683E-07
CRYM	ENSG00000103316	-1.528709052	5.8905E-07
TM4SF5	ENSG00000142484	-4.106884069	5.9155E-07
AL451069.3	ENSG00000234311	-2.343333224	6.017E-07
SCN3A	ENSG00000153253	-1.450059427	6.0999E-07
BDH2	ENSG00000164039	-1.230427285	6.4668E-07
AC099509.1	ENSG00000249746	-4.478912635	6.4923E-07
SSBP3	ENSG00000157216	-1.302870485	6.6366E-07
ECM1	ENSG00000143369	1.45538597	6.9343E-07
KCNN1	ENSG00000105642	-1.442022950	7.2573E-07
COLCA2	ENSG00000214290	-2.715546318	7.4704E-07

UGT1A1	ENSG00000241635	-1.774665541	7.7015E-07
GPR146	ENSG00000164849	2.15265535	7.9608E-07
SLC17A1	ENSG00000124568	-3.149789521	8.3087E-07
TTLL6	ENSG00000170703	-1.845090958	8.3576E-07
EMC3-AS1	ENSG00000180385	2.24436493	8.6139E-07
ADAMTS16	ENSG00000145536	1.27975699	8.7188E-07
ITPKA	ENSG00000137825	1.83606368	9.307E-07
SALL1	ENSG00000103449	-1.275712844	9.9643E-07
HOXA13	ENSG00000106031	-1.560286240	1.0701E-06
SNAP25-AS1	ENSG00000227906	-1.726918288	1.0735E-06
PROB1	ENSG00000228672	-1.793874751	1.1913E-06
NDRG1	ENSG00000104419	1.19404051	1.3095E-06
EFHD1	ENSG00000115468	-1.663637017	1.3925E-06
ZNF329	ENSG00000181894	1.66374005	1.4032E-06
CYP1B1	ENSG00000138061	1.04000761	1.4037E-06
AC008429.1	ENSG00000204758	1.64717575	1.5477E-06
RF00096	ENSG00000238840	-1.204538011	1.5654E-06
PDE2A	ENSG00000186642	1.53271462	1.6784E-06
CYP1A1	ENSG00000140465	2.86703717	1.6813E-06
BHLHB9	ENSG00000198908	-1.363269354	1.7957E-06
GRB14	ENSG00000115290	-1.010070890	1.8414E-06
GRIN3B	ENSG00000116032	-1.263024474	1.8679E-06
MDK	ENSG00000110492	-1.271051788	1.9586E-06
APOA2	ENSG00000158874	Inf	2.2153E-06
DEPDC7	ENSG00000121690	-1.203793246	2.3576E-06
SCARA5	ENSG00000168079	-1.832120132	2.4542E-06
DICER1-AS1	ENSG00000235706	-1.900495347	2.8555E-06
INTU	ENSG00000164066	-1.032156605	2.8593E-06
APOH	ENSG00000091583	-1.259109824	2.8848E-06
TXNDC16	ENSG00000087301	-1.460913607	2.9161E-06
MEIS2	ENSG00000134138	-1.198427770	2.928E-06
LINC01719	ENSG00000233396	-2.038753265	2.9776E-06
PRODH2	ENSG00000250799	-2.692680388	3.1045E-06
GREB1	ENSG00000196208	1.01773902	3.1225E-06
LINC02532	ENSG00000235142	-1.526661144	3.2634E-06
MAP3K14-AS1	ENSG00000267278	-1.278977565	3.2985E-06
CISH	ENSG00000114737	-1.488645213	3.4156E-06
AC008537.3	ENSG00000279108	-1.939026056	3.7461E-06
HOXA-AS2	ENSG00000253552	-1.600141647	3.822E-06
NKX2-5	ENSG00000183072	1.42643003	3.9305E-06
ALDOC	ENSG00000109107	1.1298441	4.0984E-06
KRT8P3	ENSG00000254285	-1.051849568	4.1767E-06
UGT1A9	ENSG00000241119	-2.647685635	4.2295E-06
ULBP1	ENSG00000111981	1.46051919	4.4307E-06
MB	ENSG00000198125	-1.804651597	4.5127E-06
BCL11B	ENSG00000127152	-1.957461755	4.7935E-06
AXIN2	ENSG00000168646	-1.455688678	4.8544E-06
AL645608.3	ENSG00000230699	-2.160808806	5.0228E-06
C5AR1	ENSG00000197405	-1.639911220	5.4281E-06
AP000757.2	ENSG00000254844	-1.482697974	5.4727E-06
CCDC146	ENSG00000135205	-1.284164833	5.4897E-06
CD14	ENSG00000170458	-2.011553746	5.9308E-06
ASF1B	ENSG00000105011	-1.016558488	6.2561E-06
COL17A1	ENSG00000065618	1.45956673	6.3342E-06
HOXB8	ENSG00000120068	-1.344137207	6.4664E-06
ITIH2	ENSG00000151655	-1.497179623	6.6856E-06
ASGR1	ENSG00000141505	-1.999674678	6.797E-06
ZNF841	ENSG00000197608	1.05075992	6.8599E-06

PYROXD2	ENSG00000119943	-1.16171793!	7.3522E-06
RBP4	ENSG00000138207	-1.723431874	7.3629E-06
SEPSECS	ENSG00000109618	-1.09171563(	7.898E-06
SRRM3	ENSG00000177679	-1.093996252	8.2304E-06
IL22RA1	ENSG00000142677	-1.11512598(	8.3196E-06
AL031009.1	ENSG00000278987	1.78335237	8.4577E-06
RETREG1	ENSG00000154153	-1.744431278	8.574E-06
IFITM3	ENSG00000142089	-1.83374367(	8.9096E-06
HBEGF	ENSG00000113070	1.05457315	9.4118E-06
AC100803.2	ENSG00000261655	-2.03936918(	9.5137E-06
CYGB	ENSG00000161544	-2.579924394	1.0331E-05
GAS6-AS1	ENSG00000233695	-1.374353423	1.0401E-05
PISD	ENSG00000241878	1.03042726	1.1059E-05
PLA2R1	ENSG00000153246	-1.159101897	1.1091E-05
SMKR1	ENSG00000240204	-1.37646924(	1.1105E-05
MIR600HG	ENSG00000236901	-1.309432148	1.1346E-05
C3orf33	ENSG00000174928	-1.653434609	1.1637E-05
CRYAB	ENSG00000109846	-2.474088063	1.256E-05
VWA1	ENSG00000179403	-1.128717578	1.2875E-05
AC108751.5	ENSG00000244503	-2.091823757	1.3716E-05
ADGRA2	ENSG00000020181	-1.334151713	1.5658E-05
TP53I11	ENSG00000175274	-1.090606803	1.5784E-05
VASH1	ENSG00000071246	-1.023255019	1.5935E-05
C4BPA	ENSG00000123838	-2.114414944	1.6937E-05
ZNF470	ENSG00000197016	1.0170833	1.6977E-05
BEX2	ENSG00000133134	1.04914301	1.7023E-05
SERPINA6	ENSG00000170099	-1.641724628	1.7424E-05
JMJD7-PLA2(	ENSG00000168970	-1.17899669(	1.7557E-05
GSDMB	ENSG00000073605	-1.43315364(	1.7877E-05
ZFYVE28	ENSG00000159733	-1.11610030(	1.8045E-05
GAL	ENSG00000069482	1.45498122	1.8276E-05
SULT1A4	ENSG00000213648	-1.93975933(	1.8581E-05
NES	ENSG00000132688	-1.51921564(	1.8801E-05
TMEM121	ENSG00000184986	-1.83628150(	1.9322E-05
FO681492.1	ENSG00000277758	-1.342816079	1.9974E-05
CEMIP	ENSG00000103888	1.25714412	2.0046E-05
NFIA	ENSG00000162599	-1.080227272	2.1115E-05
C21orf91	ENSG00000154642	-2.115311649	2.1747E-05
FIBCD1	ENSG00000130720	-1.133488379	2.1748E-05
H19	ENSG00000130600	-1.654265587	2.1808E-05
F3	ENSG00000117525	1.26780794	2.2422E-05
EXOC3L4	ENSG00000205436	-1.308609385	2.3014E-05
AC008105.1	ENSG00000233175	-1.791486979	2.3982E-05
HIGD1A	ENSG00000181061	1.04019001	2.4537E-05
MRPL49	ENSG00000149792	-1.267790285	2.4668E-05
CYB561D1	ENSG00000174151	1.42040542	2.4853E-05
IL31RA	ENSG00000164509	1.40048731	2.7256E-05
UST	ENSG00000111962	-1.331842461	2.7612E-05
ZNF229	ENSG00000278318	1.14064329	2.7847E-05
URB1-AS1	ENSG00000256073	-1.130741432	2.9991E-05
PLA2G6	ENSG00000184381	-1.110640735	3.1032E-05
LINC00342	ENSG00000232931	1.00453216	3.1451E-05
RAB3A	ENSG00000105649	-2.239913857	3.2415E-05
MCAM	ENSG00000076706	-1.010162633	3.7369E-05
PTPRN2	ENSG00000155093	-1.531164714	3.7802E-05
PDE4B	ENSG00000184588	-1.065417383	3.8361E-05
BAIAP2-AS1	ENSG00000226137	-1.070032898	3.8548E-05
PRELID3A	ENSG00000141391	1.04421235	3.8625E-05

NEURL1B	ENSG00000214357	-2.92872342	3.9009E-05
GACAT2	ENSG00000265962	-1.01397478	4.0459E-05
F2	ENSG00000180210	-1.67891370	4.0736E-05
AC023632.6	ENSG00000280123	-1.06261646	4.083E-05
ZNF792	ENSG00000180884	-1.16497836	4.0985E-05
ATP6V1B1	ENSG00000116039	-1.87449607	4.2128E-05
AC012181.1	ENSG00000261114	1.42805374	4.2514E-05
DUSP19	ENSG00000162999	-1.44516916	4.3228E-05
AC008105.3	ENSG00000267121	-1.19061313	4.409E-05
LRRN3	ENSG00000173114	-1.78063704	4.5849E-05
LCE3D	ENSG00000163202	3.00875709	4.5985E-05
SCNN1A	ENSG00000111319	-1.24533789	4.63E-05
LINC01433	ENSG00000230176	-1.54492173	4.7242E-05
PDZK1	ENSG00000174827	-1.19923928	4.8671E-05
SLC25A42	ENSG00000181035	-1.59953644	4.899E-05
AC138811.2	ENSG00000260342	Inf	4.902E-05
KRT14	ENSG00000186847	-1.64233476	5.0459E-05
AC124067.4	ENSG00000254290	-2.50241667	5.1689E-05
CERS4	ENSG00000090661	-1.34037043	5.2008E-05
AC093297.2	ENSG00000272335	-1.02213707	5.4146E-05
ASPHD2	ENSG00000128203	-1.32953095	5.4318E-05
AC123595.2	ENSG00000271762	2.09589052	5.4437E-05
HIC2	ENSG00000169635	1.06926738	5.5703E-05
NGF	ENSG00000134259	1.99909078	5.7415E-05
CTH	ENSG00000116761	-1.55669604	5.7785E-05
SGMS1-AS1	ENSG00000226200	-1.00978861	5.9815E-05
CEBPA-AS1	ENSG00000267296	-1.56972338	6.3568E-05
LINC00173	ENSG00000196668	-2.36226046	6.6275E-05
SFRP4	ENSG00000106483	-2.13868821	6.8302E-05
TNFRSF19	ENSG00000127863	1.55970593	6.9101E-05
LYPD3	ENSG00000124466	1.66593013	7.5058E-05
GATA2	ENSG00000179348	-1.16569574	7.6869E-05
AC002524.1	ENSG00000233247	-1.95801787	7.8597E-05
EHD1	ENSG00000110047	1.17961901	7.9934E-05
RF00096	ENSG00000239148	-1.02738162	8.1005E-05
C1R	ENSG00000159403	-1.01673822	8.4182E-05
FOXA3	ENSG00000170608	-2.04465097	8.6039E-05
DNAAF3	ENSG00000167646	-1.16501098	8.6402E-05
CAPN12	ENSG00000182472	-1.64267624	8.7335E-05
MIR222HG	ENSG00000270069	1.70988913	8.927E-05
SPRY1	ENSG00000164056	-1.12540152	9.2284E-05
ZNF165	ENSG00000197279	1.55272035	9.3053E-05
EDARADD	ENSG00000186197	-1.18291444	9.3287E-05
OCEL1	ENSG00000099330	-1.27366584	9.3882E-05
OTX1	ENSG00000115507	-1.05418102	9.5249E-05
CYP2U1	ENSG00000155016	-1.31016014	9.9962E-05
RARRES1	ENSG00000118849	-1.25724430	0.00010164
GLIS3	ENSG00000107249	-1.07252165	0.00010891
CKMT1B	ENSG00000237289	-1.21312959	0.00010946
MKL2	ENSG00000186260	-1.00845391	0.00011104
C3orf18	ENSG00000088543	-1.05806492	0.00011393
NOV	ENSG00000136999	-1.18714426	0.00011658
FRAT1	ENSG00000165879	-1.18795600	0.00012069
CRYM-AS1	ENSG00000189149	1.58926163	0.00013073
KCNJ2	ENSG00000123700	-1.34598600	0.00013424
TMOD1	ENSG00000136842	1.0590703	0.00013458
MT1X	ENSG00000187193	1.36672152	0.00013518
PAPLN	ENSG00000100767	-1.05690470	0.00013567

ANXA9	ENSG00000143412	-1.61986877[illegible]	0.00013601
PALM	ENSG00000099864	-1.34420093[illegible]	0.00014002
MICALL1	ENSG00000100139	1.0302605	0.00014406
MISP3	ENSG00000141854	-1.21913428[illegible]	0.00014611
SYDE2	ENSG00000097096	1.04778884	0.00014683
TMEM268	ENSG00000157693	1.02663131	0.00014732
TMEM151A	ENSG00000179292	-1.19894601[illegible]	0.00014771
HRASLS2	ENSG00000133328	-4.22782214[illegible]	0.00014837
NUDT11	ENSG00000196368	-1.04347649[illegible]	0.00015216
CTSH	ENSG00000103811	-1.00267424[illegible]	0.00015991
CFHR1	ENSG00000244414	-2.84502341[illegible]	0.00016053
BAAT	ENSG00000136881	-1.36422606[illegible]	0.00016249
BCL2A1	ENSG00000140379	1.26749892	0.00016562
AC090197.1	ENSG00000253837	1.38779941	0.00017493
AC092683.1	ENSG00000230606	1.28196805	0.00017923
AC106782.1	ENSG00000258130	-1.36010424[illegible]	0.00018319
TMEM263	ENSG00000151135	-1.23248555[illegible]	0.00018541
TRIM46	ENSG00000163462	-1.06753504[illegible]	0.00018837
AC008915.2	ENSG00000260136	1.14455531	0.00019324
RASA4	ENSG00000105808	-1.14445175[illegible]	0.00020904
HLA-DMA	ENSG00000204257	-1.47148302[illegible]	0.00021073
RNF185	ENSG00000138942	1.35519785	0.00021754
FAM111A	ENSG00000166801	-1.25223847[illegible]	0.0002287
PRKAR2B	ENSG00000005249	-1.29619664[illegible]	0.00024048
FTCDNL1	ENSG00000226124	-1.19044512[illegible]	0.00024723
KRT19	ENSG00000171345	1.07625154	0.00025426
MLXIPL	ENSG00000009950	-1.28068516[illegible]	0.00026036
S1PR5	ENSG00000180739	-2.53860702[illegible]	0.00026252
ACY3	ENSG00000132744	-2.27155532[illegible]	0.00026976
LRRC37A17P	ENSG00000263142	-1.97497872[illegible]	0.00027112
VPS37D	ENSG00000176428	-1.12639793[illegible]	0.00027372
LRP12	ENSG00000147650	-2.04553475[illegible]	0.0002742
LINC02454	ENSG00000256268	3.27539845	0.00029401
LAT2	ENSG00000086730	1.06646986	0.00032533
IFNL4	ENSG00000272395	-2.09033623[illegible]	0.00033645
AC159540.2	ENSG00000277701	1.43348662	0.00034518
ARSI	ENSG00000183876	1.09244597	0.0003783
TCN1	ENSG00000134827	-2.73803668[illegible]	0.00037932
AP000523.1	ENSG00000215270	-1.22997516[illegible]	0.00041102
TM4SF19	ENSG00000145107	1.94322426	0.00042097
PRKCG	ENSG00000126583	-1.30952267[illegible]	0.00042733
ABLIM3	ENSG00000173210	1.85875395	0.0004469
AC103740.2	ENSG00000259727	3.19379429	0.00045766
C7orf55-LUC7	ENSG00000269955	Inf	0.00047532
PPM1K	ENSG00000163644	-1.02750391[illegible]	0.00048094
PCYOX1L	ENSG00000145882	-1.04582426[illegible]	0.00050166
DPEP1	ENSG00000015413	-1.59509795[illegible]	0.00051222
AC211429.1	ENSG00000242073	3.67210005	0.00051952
AC100757.1	ENSG00000273679	-1.40623678[illegible]	0.00053466
AC073611.1	ENSG00000257605	1.94031835	0.00054681
AC068491.4	ENSG00000279191	1.59410164	0.00056828
FBXO2	ENSG00000116661	-1.11633602[illegible]	0.00057115
ZEB1	ENSG00000148516	-3.0532996	1.4379E-05
CCL24	ENSG00000106178	-2.94530293[illegible]	0.00057253
KPNA7	ENSG00000185467	2.03306561	0.00057445
RAB30	ENSG00000137502	-1.06287332[illegible]	0.00060273
NRSN2	ENSG00000125841	-1.45884723[illegible]	0.00060609
LINC01942	ENSG00000253428	-2.75905043[illegible]	0.00061012

CFD	ENSG00000197766	-1.552124727	0.00065089
DEFB1	ENSG00000164825	-2.716579844	0.00066097
TUBA4B	ENSG00000243910	-2.707094697	0.00066174
AL022238.4	ENSG00000284431	-2.436341307	0.0006639
QPRT	ENSG00000103485	-2.245398701	0.00066781
MAPK7	ENSG00000166484	1.21593337	0.00067869
LINC00313	ENSG00000185186	-1.347981799	0.00071651
AC008687.6	ENSG00000269706	4.7249395	0.0007443
LGI3	ENSG00000168481	-1.544117447	0.00075833
HOXC6	ENSG00000197757	-1.247535197	0.00076513
MEG3	ENSG00000214548	1.06056775	0.00077187
RPPH1	ENSG00000277209	-1.796630629	0.00078504
GDPD1	ENSG00000153982	-1.240984401	0.00079079
LRRC8D	ENSG00000171492	-1.583116987	0.0008056
RCBTB2	ENSG00000136161	1.48839353	0.00080808
DNLZ	ENSG00000213221	1.11326596	0.00081412
KLHDC1	ENSG00000197776	-1.776962367	0.00086119
C3orf80	ENSG00000180044	-1.472111580	0.00086387
GUCY2EP	ENSG00000204529	-1.913455009	0.00094794
MIR193BHG	ENSG00000262454	-1.148980274	0.00094988
STK38L	ENSG00000211455	1.20109342	0.00095955
SH3BP1	ENSG00000100092	-1.063376333	0.00096409
EPCAM	ENSG00000119888	-1.805342762	0.00096748
LINC00482	ENSG00000185168	-1.022107164	0.00097594
TDG	ENSG00000139372	-1.118744836	0.00098932
RIBC2	ENSG00000128408	-1.573684386	0.00099426
DLG3	ENSG00000082458	-1.119106253	0.00100075
CYS1	ENSG00000205795	-1.376609989	0.00101513
LINC00488	ENSG00000214381	-1.187198059	0.00102011
TOLLIP	ENSG00000078902	1.00100453	0.00104817
ARL14	ENSG00000179674	1.26262204	0.00105793
VIM	ENSG00000026025	1.08494895	0.00109171
IQCD	ENSG00000166578	-1.427327250	0.00110221
AKR1C4	ENSG00000198610	-1.253214261	0.00114444
AL137003.1	ENSG00000229931	1.68957023	0.00117867
UNC5CL	ENSG00000124602	-1.144534791	0.00118265
TSKU	ENSG00000182704	-1.515257297	0.00126698
TRAPPC2B	ENSG00000256060	1.22103634	0.00128871
KCNJ2-AS1	ENSG00000267365	-1.934962285	0.00133556
HAPLN3	ENSG00000140511	-1.205301591	0.00136971
RDH5	ENSG00000135437	-1.423327511	0.0014005
AC005670.2	ENSG00000262633	Inf	0.00140908
LINC02313	ENSG00000258474	-1.778816862	0.00148346
IFNL3P1	ENSG00000268510	-1.744340201	0.00149682
AL049794.1	ENSG00000273998	4.64343529	0.00151068
RGS14	ENSG00000169220	-1.241838886	0.00151511
AGMO	ENSG00000187546	-1.529143150	0.0015188
ADAM19	ENSG00000135074	-1.024068430	0.00153947
NEURL2	ENSG00000124257	-1.621945056	0.00154041
ELF3-AS1	ENSG00000234678	-1.097924409	0.00154572
LCA5	ENSG00000135338	-1.228950133	0.00159498
TMEM81	ENSG00000174529	-1.493214637	0.00165634
AC090192.2	ENSG00000253227	1.46954311	0.001659
PRRT1B	ENSG00000283526	-1.967411186	0.00168957
PAOX	ENSG00000148832	-1.161418590	0.00173655
LINC02057	ENSG00000249279	1.56078805	0.00175871
CARD8-AS1	ENSG00000268001	1.68084664	0.00178278
PPARA	ENSG00000186951	-1.389869439	0.00179578

KRT80	ENSG00000167767	1.15943619	0.00186619
AP000275.2	ENSG00000265590	-5.342845404	0.00196245
SPATA32	ENSG00000184361	-1.413674018	0.00205648
NCCRP1	ENSG00000188505	-1.555352755	0.00211339
NALT1	ENSG00000237886	-1.700357588	0.00220182
LINC02210	ENSG00000204650	-1.238499716	0.00225413
AL645608.9	ENSG00000273443	1.32860651	0.00230035
PCDHAC1	ENSG00000248383	-1.030029991	0.00233761
BCL2L2	ENSG00000129473	1.86869106	0.00236269
DEPTOR	ENSG00000155792	-1.247675614	0.00236897
TMEM75	ENSG00000280055	1.72372774	0.00243512
AC005020.2	ENSG00000272647	-2.266597790	0.00244391
PRRT2	ENSG00000167371	-1.471859069	0.00244929
ANO1	ENSG00000131620	1.0044024	0.00246232
GPR1	ENSG00000183671	-1.414663677	0.00246835
AC068888.1	ENSG00000257337	-1.130211096	0.00253334
SLX1A-SULT1	ENSG00000213599	-1.133409369	0.00253709
LGALS2	ENSG00000100079	-3.383124388	0.00256837
TLE6	ENSG00000104953	-1.228909180	0.00257399
CCDC184	ENSG00000177875	-1.056237720	0.00262533
FBXO27	ENSG00000161243	1.14745511	0.00271873
DLG5-AS1	ENSG00000233871	-1.678243309	0.0027191
SMPD1	ENSG00000166311	1.14508133	0.00272486
AC006486.1	ENSG00000268643	5.29866784	0.00279976
HBQ1	ENSG00000086506	-1.092611275	0.0028226
PRPS2	ENSG00000101911	-1.080626615	0.00283251
EGFR-AS1	ENSG00000224057	1.51701069	0.00283531
CHST13	ENSG00000180767	-1.551596880	0.00283722
RGL3	ENSG00000205517	-1.154669231	0.00285936
PSCA	ENSG00000167653	1.37338022	0.00295394
CXXC5	ENSG00000171604	-1.562050217	0.00300943
UGT1A2P	ENSG00000228445	-1.512920589	0.00301896
RAB26	ENSG00000167964	-1.661421636	0.00303734
AL139246.5	ENSG00000272449	-1.623767050	0.00316746
AL732372.3	ENSG00000250575	1.12860782	0.00328434
RCOR2	ENSG00000167771	-1.376937726	0.00335733
RARB	ENSG00000077092	-1.552777904	0.00343633
AC017116.1	ENSG00000239775	-3.324398873	0.00345361
AL356019.2	ENSG00000258768	-1.213816762	0.00348052
AC096887.2	ENSG00000280417	-1.602799156	0.00356358
AC133644.2	ENSG00000273445	-1.724366508	0.00366967
AP002807.1	ENSG00000255031	-1.203676068	0.0037196
AC112220.4	ENSG00000271643	1.2431675	0.00375071
RPL13AP20	ENSG00000234498	1.22098717	0.00404749
ZNF461	ENSG00000197808	1.10590086	0.00409941
AL807757.2	ENSG00000236095	-1.538941703	0.00415806
RTP4	ENSG00000136514	-2.207824450	0.00418934
RASAL2-AS1	ENSG00000224687	1.27252442	0.00423591
MAP1LC3B2	ENSG00000258102	1.07154764	0.00426224
GAPLINC	ENSG00000266835	-2.332011580	0.00428684
TTLL11	ENSG00000175764	1.00576922	0.00455302
MGMT	ENSG00000170430	-1.311497446	0.00456516
AC011676.5	ENSG00000280035	-1.549315813	0.00462774
ZNF223	ENSG00000178386	1.74685871	0.00481843
AC073109.1	ENSG00000235920	-2.068429240	0.00488671
PRR22	ENSG00000212123	1.07160005	0.00500092
GPATCH2L	ENSG00000089916	1.26956965	0.00502135
KCNG3	ENSG00000171126	-1.131809720	0.00507916

AC013444.2	ENSG00000270390	-1.80563836	0.00511882
AC108463.3	ENSG00000271590	1.92379091	0.00515289
AL133367.1	ENSG00000260285	-1.12350152	0.00521242
AC107021.2	ENSG00000261051	1.17906385	0.00558881
S100A9	ENSG00000163220	-1.87208059	0.00565602
DMBX1	ENSG00000197587	-1.16997971	0.00568243
GSEC	ENSG00000280832	-1.03399617	0.00596872
BST2	ENSG00000130303	-1.97792749	0.00597992
LINC01468	ENSG00000231131	-1.91981104	0.00598639
ETNK2	ENSG00000143845	-1.40686185	0.00606266
IDH1-AS1	ENSG00000231908	-1.96663615	0.00616831
GJB3	ENSG00000188910	1.37942019	0.00619157
AL138759.1	ENSG00000244332	-1.16279175	0.00637951
AC073896.1	ENSG00000144785	-1.55935735	0.00640117
AC067930.4	ENSG00000254859	-1.35363010	0.00644069
AC126474.2	ENSG00000280088	-1.06493845	0.006597
AL161668.1	ENSG00000178107	-3.34440936	0.00667972
SOCS2	ENSG00000120833	1.52334551	0.00704603
SLC6A6	ENSG00000131389	1.13169718	0.00742194
TOB1	ENSG00000141232	-1.00009469	0.00767668
TMEM169	ENSG00000163449	-1.45846200	0.00795592
ADM5	ENSG00000224420	1.26246698	0.00802725
AL133355.1	ENSG00000260461	-1.04064502	0.00815493
AC004231.3	ENSG00000265359	-Inf	0.00841165
OSER1-AS1	ENSG00000223891	1.44927845	0.00847864
ZNF181	ENSG00000197841	1.02013238	0.0084958
CCDC96	ENSG00000173013	1.11245177	0.00850062
AC105052.1	ENSG00000205236	1.10389966	0.00859647
AC008537.2	ENSG00000269843	-1.45936889	0.00886824
RGCC	ENSG00000102760	-2.43166165	0.00897983
RCN3	ENSG00000142552	-1.33053907	0.00935683
HOXA2	ENSG00000105996	-1.25696654	0.00937695
LINC01843	ENSG00000251169	-1.05886667	0.00963807
HSPA2	ENSG00000126803	-1.36367691	0.0099745
LTB	ENSG00000227507	-1.01522520	0.01009086
AC026464.3	ENSG00000260371	-3.66016872	0.01018295
FCGRT	ENSG00000104870	-1.07616896	0.01020522
AC073569.2	ENSG00000258048	-1.58953245	0.01024692
DUSP9	ENSG00000130829	-1.29719961	0.01042075
CDKL2	ENSG00000138769	1.05268738	0.01042961
CNFN	ENSG00000105427	-1.15647806	0.01053179
SLC35A5	ENSG00000138459	-1.80502850	0.01061123
DUX4L9	ENSG00000224807	-1.57318903	0.01064809
BDKRB2	ENSG00000168398	-1.01273494	0.0107397
LINC02298	ENSG00000257556	-1.21766223	0.01095472
AL137802.2	ENSG00000261135	-1.99839982	0.01111683
AC087632.1	ENSG00000259316	-1.10919578	0.01114417
AC110285.2	ENSG00000262877	-1.30210549	0.01115803
C20orf141	ENSG00000258713	-1.69664097	0.0112556
PWAR5	ENSG00000279192	-1.08073804	0.01156233
TNPO1P3	ENSG00000229586	1.25879856	0.01156572
AC008105.2	ENSG00000233483	-1.25546821	0.01165155
RRAD	ENSG00000166592	1.19760882	0.01234759
AC023024.2	ENSG00000279970	-1.01173082	0.01248529
MAST4-AS1	ENSG00000229666	-2.03897709	0.01251559
UPK3BL2	ENSG00000284981	5.13237232	0.01278894
P2RY11	ENSG00000244165	1.15633271	0.01301377
EFNA2	ENSG00000099617	-2.19826091	0.013169

AC009283.1	ENSG00000273576	-1.07482674[illegible]	0.01336757
LINC01474	ENSG00000236849	-1.85554026[illegible]	0.01350842
AC011466.3	ENSG00000269534	-2.23866226[illegible]	0.01352024
AC137932.2	ENSG00000261253	1.40100666	0.01372448
SCG5	ENSG00000166922	1.04187735	0.0138613
KRT20	ENSG00000171431	-1.32252483[illegible]	0.01386452
AL512844.1	ENSG00000232952	1.63726698	0.01402281
RAP1AP	ENSG00000258769	Inf	0.01442475
AC112220.2	ENSG00000271020	1.17482619	0.01447198
RN7SL23P	ENSG00000240823	-Inf	0.01456708
MT1F	ENSG00000198417	1.52505538	0.01458042
AC006538.1	ENSG00000261342	-2.10466113[illegible]	0.01470407
RN7SL5P	ENSG00000265735	1.09435516	0.01510819
AC027117.1	ENSG00000253671	-1.69421933[illegible]	0.01559665
AC090587.2	ENSG00000229368	-1.58029754[illegible]	0.01565483
OVGP1	ENSG00000085465	-1.28303472[illegible]	0.01700115
NIPSNAP3B	ENSG00000165028	-1.08393210[illegible]	0.01710129
AC027702.1	ENSG00000260588	1.14626182	0.01774658
HOXD11	ENSG00000128713	-1.32461235[illegible]	0.01781696
FLVCR1-AS1	ENSG00000198468	-1.03209344[illegible]	0.01781709
CLDN12	ENSG00000157224	1.11460992	0.01819117
AC011448.1	ENSG00000258674	1.45748492	0.01860489
CX3CL1	ENSG00000006210	-1.69757083[illegible]	0.01864159
AC005730.2	ENSG00000261033	2.12642028	0.01870496
TSPAN12	ENSG00000106025	-1.05955637[illegible]	0.01874237
AC002467.1	ENSG00000241764	1.21491302	0.01894269
AC004803.1	ENSG00000250132	-1.14758113[illegible]	0.01908662
AP001574.1	ENSG00000253217	-1.85921440[illegible]	0.01912367
AASDHPPT	ENSG00000149313	-1.19737061[illegible]	0.01951517
RNF103-CHM	ENSG00000249884	2.56304021	0.02018159
BASP1-AS1	ENSG00000215196	-1.04992225[illegible]	0.02077096
AC018690.1	ENSG00000273306	1.41597461	0.02151948
AC011445.1	ENSG00000268262	-1.41818317[illegible]	0.02176842
KRT83	ENSG00000170523	-1.21118604[illegible]	0.02224344
AD000671.1	ENSG00000188223	Inf	0.02240402
AC131212.2	ENSG00000279700	-1.52316265[illegible]	0.02276161
AC008083.2	ENSG00000258181	-1.55048113[illegible]	0.02303335
AC012181.2	ENSG00000261270	1.2836998	0.02403276
AC126544.1	ENSG00000280022	-2.61049980[illegible]	0.02434657
SERPINB3	ENSG00000057149	-1.00554950[illegible]	0.02487375
AC004918.1	ENSG00000244701	-1.01241340[illegible]	0.02499296
TRIM74	ENSG00000155428	-1.34321985[illegible]	0.02520691
AL139289.1	ENSG00000229431	2.35286166	0.02528089
AC113404.3	ENSG00000254893	-1.24658452[illegible]	0.02552758
MALAT1	ENSG00000251562	-1.38168272[illegible]	0.02680626
SP2-AS1	ENSG00000234494	-1.28346737[illegible]	0.02701884
BCRP3	ENSG00000215481	-1.47543965[illegible]	0.02792913
FRMD6-AS1	ENSG00000273888	1.06236934	0.02797896
RPS10-NUDT	ENSG00000270800	4.40335956	0.02810169
PTGER2	ENSG00000125384	-1.02011893[illegible]	0.02812332
DGCR10	ENSG00000273164	-3.62425611[illegible]	0.02874729
VPS33B	ENSG00000184056	-1.34063266[illegible]	0.02897822
UPK3BL1	ENSG00000267368	-4.79403004[illegible]	0.02922971
LINC01970	ENSG00000265692	-1.50940774[illegible]	0.0295659
ZNF830	ENSG00000198783	1.00060339	0.02985294
AL356056.2	ENSG00000231187	-1.18185319[illegible]	0.03019431
AC010809.2	ENSG00000259408	1.69013432	0.03103661
PCOTH	ENSG00000205861	1.92724929	0.03121287

FAM35BP	ENSG00000165874	-1.343902773	0.03138343
AC026368.1	ENSG00000277840	-1.205789026	0.03165312
DCST1-AS1	ENSG00000232093	-1.155446070	0.03204717
ZNF84	ENSG00000198040	2.08124395	0.0331159
RPL23AP95	ENSG00000236848	-Inf	0.03348931
LINC01220	ENSG00000259687	-2.169645981	0.03401703
AL035563.1	ENSG00000273148	-1.207577613	0.03417483
AC243732.1	ENSG00000274756	-1.280431213	0.034493
AC245033.3	ENSG00000278603	-1.219054097	0.03458549
PPP1R18	ENSG00000146112	1.00583978	0.03520074
AC135279.1	ENSG00000255566	2.13080279	0.03559461
TFRC	ENSG00000072274	-1.252909114	0.03637297
ATP2C2-AS1	ENSG00000261286	1.2320736	0.03660579
ELOCP19	ENSG00000241975	1.76198924	0.03698131
SPANXB1	ENSG00000227234	1.69157768	0.03707248
AL627309.6	ENSG00000268903	1.86603213	0.03710577
CBLL1	ENSG00000105879	1.63820083	0.03759458
AC067838.1	ENSG00000272338	-2.807398705	0.03837697
TTPAL	ENSG00000124120	2.35399307	0.03844685
AC105052.2	ENSG00000213385	1.4525555	0.03873709
AC098487.1	ENSG00000248161	-1.040444016	0.03877655
CHCHD4P3	ENSG00000236156	1.75757669	0.03881974
CLEC11A	ENSG00000105472	-1.332049621	0.03925539
ASB1	ENSG00000065802	1.9721487	0.03932045
TUT1	ENSG00000149016	-1.086603641	0.03995411
AP000695.2	ENSG00000233818	1.14602065	0.04035844
AC017100.1	ENSG00000259985	1.06240854	0.04064011
AD000671.3	ENSG00000267439	1.14403154	0.04112137
AC087269.1	ENSG00000254367	-1.182379847	0.04127544
HIVEP1	ENSG00000095951	1.35474486	0.04130847
KATNBL1P6	ENSG00000228283	1.06418086	0.04139572
LINC01564	ENSG00000235899	1.20967425	0.04152259
PAN3-AS1	ENSG00000261485	-1.267676575	0.04193577
AC233300.1	ENSG00000279155	1.68473217	0.04242816
GCHFR	ENSG00000137880	-1.797638992	0.04274547
AC004231.1	ENSG00000234477	-1.980830726	0.04317504
USP49	ENSG00000164663	-1.031883522	0.04366956
AC132942.1	ENSG00000240898	1.69054137	0.04534162
GATA3	ENSG00000107485	1.11164138	0.04538594
AC027309.2	ENSG00000253683	-1.192462855	0.04595893
AC010323.1	ENSG00000167774	-Inf	0.04596124
AL158163.2	ENSG00000278601	1.51443133	0.04603075
AC133681.1	ENSG00000239620	2.84464934	0.04636847
TMEM250	ENSG00000238227	2.44735616	0.04678088
AL133410.1	ENSG00000227388	1.3616287	0.04692054
CYP2F2P	ENSG00000237118	-1.268165682	0.04789333
PLS3-AS1	ENSG00000271826	-1.122496532	0.04896578
AC040160.1	ENSG00000262691	-1.018119859	0.04918384

Supplementary Table 5

Relationship between LINC01977 expression and clinicopathological parameters of LUAD				
**Variable**	**No. patients**	**LINC01977 expression**		***P* value**
		**High expression**	**Low expression**	
**Age (yr)**				
＞62	109	53	56	0.669
≤62	77	35	42	
**Sex**				
Female	86	43	43	0.496
Male	100	45	55	
**Smoking**				
Yes/Ever	98	54	44	0.395
Never	88	43	45	
**Clinical stage**				
Early (I+IIA)	91	45	46	**0.008**
Advanced (IIB+III)	95	29	66	
**pT status**				
T1-T2	174	84	90	0.482
T3-T4	12	4	8	
**Lymph node metastasis**				
No metastasis (N0)	91	59	32	**<0.001**
Metastasis (N1+N2)	95	29	66	

Supplementary Table 6

	DNA sequence	Location
**E1**	ccaccacactcccaccacagtcagctccccagaatcaagctcatcaggtctagctctccct<br>ccctgcaggcagagctcgcaggactgtgccagctagggccacgccctcagcctcaagcc<br>ccatgccccagccactctgagctctcttgctgcacttacataagccatgctatgatggtttaaat<br>atgtctcctgaagttcacgtgttgaaaacttattcctgccacaggatgttgagaagtggggccta<br>acaaaggtaatgaggtcatgagagtctgccctcatgaatagattaatgtagttattgtgggagtg<br>ggttcattatcacgagggtgggtttg	chr17:79460389-79460729
**E2**	tacattgaagtcctaacccctagtacgttagatgtgaccgtatttggagataggacccttaccag<br>gccatatgggtgggcctcatccactctgaccagtgtccttctaagagaagaagattaggacac<br>agacatgcacagatcaaggggcggccatgtgaggacataaggagaagatgaccatctaca<br>agccaaagagaagccttcaaggaaaccaatgctgccaacagcttgatcttagacttctggcc<br>tcttgaattttgagaaaattaacctctgttgtgtgagcccccaggctgaggggcttcaccatggc<br>agcccaagccgaccacacacctcccacatctcatgaccaccctccattctcaagcgagga<br>cactgccctgggtctgttggtcagtggcagagcttattcaaacgtgacaagttctgtgatctgac<br>ctgtgtggcctctataccggcacttctcaaaccatctgtggtgaaggattcattggtttatttccaat<br>tcattctggacctacatttttgtttctttgttttgtttttgagacagggtctcactatgctgcccagctga<br>tctcaaacaccagggctcaaccaatcctcccacctcagcctcctgagtagttgaagtgacaa<br>gcccttacttttggtttttagaaaacctataaaaatagaatcactagaaaaatggaataaagaag<br>gcatacaaaacacaaagcccaggttcttaacgattagactcatcagacctaaaacgactcct<br>tcaaatgactaaaagattctaaatgcttaatttcagcttctgtactcagttgcagggatcaggagc<br>agcacctcacgggctggcacccatccacagacctcactttgagtagctgagatatgggtcat	chr17:79472376-79473272
**E3**	gggaggtgggggcaggggtactgggggcagggatggggatgtgggaaggtgggggaggg<br>gtacagagggtggggaggtgggggaaagggtactggggcagggatgggggtgcaggaag<br>gtgggaatggaatgcatgggacaggcacaggtacataaaggtggttgggggcacaggggtg<br>cagatggagggaggtgggggagagggtatgacagggtagggcacggggcaggggaggg<br>gtgcagacatccacagtctgggtgcaggtgtagggtggcagaaggtggttgggggtgtaggc<br>ggtggacggggtggaggttaaggcagaggcccctgtgggcagggaccctgcaggccggg<br>accctgcaggcctccagcctcggcacagcctgagcagtggcagcgtggcagatgggaac<br>gtgcagaaacatgcagaagcatggcccctggttaataagattaggatgttgaacggccgaca<br>tgttctccgagactggctgtatgccacaaatggaggggactgcgaccttaatagaatgctgaa<br>cttttggaaagtggagcaaaatggagagagtcagagttggggactgagagtccccctctcgg<br>gctggaggcc	chr17:79495539-79496152
**E4**	tgcttaatttactccaacccaaacagtgtatgtgtgccatcagccagacaggacacaaaaac<br>ggtttaggagatccactctgattagcctgagagactgtctctgggacagagtcatgagatcagg<br>gtggccagggagtggctgagctgtgtctccctgaatccccccaccactggcaccacccag<br>aaatttcacaacggagttttaaacagcaacacacaacaataataaatctaccagtttgtttctttt<br>cctaaaatccagtcactggacagagtaccacccccataccgaagaaccgaagagcagaa<br>ccagcatcacagagagcccagcaaccccggagcaatgcctggggaacatctgccctttca<br>gggaccattggtacagagcccagtgcccactgctgggtgtgggagccactcttcaatggcaa<br>aagtgtaagtaataggaacagttccatggatgaaatgcagccagggaggtggctgggagag<br>ggcct gggtgcagggggcctgg	chr17:79483969-79484486
**E5**	gggaggtgggggcaggggtactgggggcagggatggggatgtgggaaggtgggggaggg<br>gtacagagggtggggaggtgggggaaagggtactggggcagggatgggggtgcaggaag<br>gtgggaatggaatgcatgggacaggcacaggtacataaaggtggttgggggcacaggggtg<br>cagatggagggaggtgggggagagggtatgacagggtagggcacggggcaggggaggg<br>gtgcagacatccacagtctgggtgcaggtgtagggtggcagaaggtggttgggggtgtaggc<br>ggtggacggggtggaggttaaggcagaggcccctgtgggcagggaccctgcaggccggg<br>accctgcaggcctccagcctcggcacagcctgagcagtggcagcgtggcagatgggaac<br>gtgcagaaacatgcagaagcatggcccctggttaataagattaggatgttgaacggccgaca<br>tgttctccgagactggctgtatgccacaaatggaggggactgcgaccttaatagaatgctgaa<br>cttttggaaagtggagcaaaatggagagagtcagagttggggactgagagtccccctctcgg<br>gctggaggcc	chr17:79495539-79496152
**E6**	gacaggcccattcactgggcgggctcagccgccctgccacacctccacatgtccagcgga<br>aggtcccagacccttcatctcgctggccagagaggctggttgtctgcagccctcaggcccttc<br>ttcctgcctgtaaaagcctcacagctaccaaagaagagatgtccaacagctccaggaaacc<br>tgagtcactggggttggtgggggaggccacttttgttcccgagtcccaccctctgcctcctc	chr17:79501268-79501513

Supplementary Table 7. Cloning primers, RACE primers and lncRNA ASO sequences

**Cloning primers**	
LINC01977(2791)	Primer-F: AATTTTCCACTGTCTCTCAGTATCC
	Primer-R: TTTTTTTTTTTTTTAGAGGACAAAGCACA
LINC01977(2295)	Primer-F: GCAGAAGGAAGAGAGCCTT
	Primer-R: TTTTTTTTTTTTTTAGAGGACAAAGCACAC
LINC01977(1799)	Primer-F: CGATCTCCTGACCTCGTG
	Primer-R: TTTTTTTTTTTCACAGAGGACAAAGCA
LINC01977(1299)	Primer-F: GGTGGGAGAGTCGGAGT
	Primer-R: TTTTTTTTTTTCACAGAGGACAAAGCA
LINC01977(799)	Primer-F: GCCGTGTCATTTCGGAGG
	Primer-R: TTTTTTTTTTTCACAGAGGACAAAGCA
LINC01977(299)	Primer-F: ACAGACACGCACACGCA
	Primer-R: TTTTTTTTTTTCACAGAGGACAAAGCA
Enhancer-1	Primer-F: CCACCACACTCCCACCACAG
	Primer-R: CAAACCCACCCTCGTGATAATGA
Enhancer-2	Primer-F: TACATTGAAGTCCTAACCCCTAGTACG
	Primer-R: ATGACCCATATCTCAGCTACTCAAAGT
Enhancer-4	Primer-F: TGCTTAATTTACTCCAACCCAAACAGTGTATGTGTG
	Primer-R: CCCAGGCCCCCTGCACC
Enhancer-6	Primer-F: GACAGGCCCATTCACTGGGC
	Primer-R: GAGGAGGCAGAGGGTGGGA
ZEB1-P(0-100)	Primer-F: GAGCTGGAAAGTTTTTTTTTCCTTTCT
	Primer-R: ACCGAATCAGGTCATAGACTATGG
ZEB1-P(300-400)	Primer-F: AGACATAATGTATAGCAAAGAATATTTACAGT
	Primer-R: TTTTCTCTCCCACATTTTCTTGGG
ZEB1-P(1200-1300)	Primer-F: AGGCTGCTGGCAAGCGG
	Primer-R: TGGTTTCCCCCCAAGCGAACC
ZEB1-P(1400-1500)	Primer-F: CCTCATCAAGGGAACTCCCCG
	Primer-R: GGCTACCATCAGTCCCACGC
**RACE primers**	
5' RACE	Primer-R1: CTGCCAACAAGTCTGGGGAGAGAAGAGAGT
	Primer-R2: CGAGCTGAGAAATCCAGGGAAGACGAA
3' RACE	Primer-F1: CCCACCCCAAGAGGCTGTCTAACG
	Primer-F2: GGGAAACAATGAGAAACCAGATACCATGGA
**LncRNA ASO sequences**	
LINC01977-ASO-1	TCACCCAGATGGAGTTTCAA
LINC01977-ASO-2	GAGCGTAGAGCCGTGTCATT
LINC01977-ASO-3	GCCTTATCCAAACAGTTCAC
**sgRNA sequences**	
CREBBP sg#1	GCGTCCACAGCAATATCCAA

CREBBP sg#2	AGACCGCACCTGGTTACTAA
CREBBP sg#3	AGCTCTAAAGGATCGCCGCA
EP300 sg#1	GTACGACTAGGTACAGGCGA
EP300 sg#2	ATACGAGGCCCATAGCCCAT
EP300 sg#3	CGACCATCCATCAGATCGCA
SMAD3 sg#1	TGCGGCTCTACTACATCGGA
SMAD3 sg#2	GCTCTACTACATCGGAGGGG
SMAD3 sg#3	GCGGCTCTACTACATCGGAG

The DNA fragments of enhancer-3, -5 and LINC01977 promoter region were synthesized and cloned into the pLG3 plasmid. P refers to promoter region. The sgRNA used in this study was prepared by in vitro transcription from

Supplementary Table 8. qRT-PCR primers used in this study

Genes	Primers sequences
LINC01977	Primer-F: TGACAGTGTAACAGGTATTCGCC
	Primer-R: GGCTCTACGCTCATTCCCA
GAPDH	Primer-F: CCATGGGGAAGGTGAAGGTC
	Primer-R: GAAGGGGTCATTGATGGCAAC
ACTB	Primer-F: ACCTTCTACAATGAGCTGCG
	Primer-R: CCTGGATAGCAACGTACATGG
U6	Primer-F: ATTGGAACGATACAGAGAAGATT
	Primer-R: GGAACGCTTCACGAATTTG
PAI-1	Primer-F: AATCAGACGGCAGCACTGTCT
	Primer-R: GGCAGTTCCAGGATGTCGTAGT
COL1A1	Primer-F: TCCGACCTCTCTCCTCTGAA
	Primer-R: AGGGGGAAAAACTGCTTTGT
Vimentin	Primer-F: TCGCCATCAGTAGAAGGTAGCA
	Primer-R: CTCAATGTCAAGGGCCATCT
E-cadherin	Primer-F: TGGAGAGACACTGCCAACTG
	Primer-R: TTAGGGCTGTGTACGTGCTG
N-cadherin	Primer-F: GGATCAACCCCATACACCAG
	Primer-R: TGGTTTGACCACGGTGACTA
Snail	Primer-F: TTCTCACTGCCATGGAATTCC
	Primer-R: GCAGAGGACACAGAACCAGAAA
WBP2	Primer-F: TCAAGCAGCCCGTATTTGGTG
	Primer-R: GCCGTGAAAGTCAACTTGTAGGA
HSPA9	Primer-F: GGAAGGTAAACAAGCAAAGGTGC
	Primer-R: CCAACAAGTCGCTCACCATCT
CPNE1	Primer-F: CACTGCGTGACCTTGGTTCA
	Primer-R: CTCCCACATCCTGTAAAAGGAC
SYPL1	Primer-F: AAGATTACGTCCTCATAGGCGA
	Primer-R: TCGTGTAGCCAACATAAAGCAG
HAX1	Primer-F: AGCATCTTCAGCGATATGGGG
	Primer-R: CCTCCCGTAGTCTCTCACCA
HMGB1	Primer-F: TATGGCAAAAGCGGACAAGG
	Primer-R: CTTCGCAACATCACCAATGGA
SMAD3	Primer-F: TGGACGCAGGTTCTCCAAAC
	Primer-R: CCGGCTCGCAGTAGGTAAC
ZEB1	Primer-F: GATGATGAATGCGAGTCAGATGC
	Primer-R: ACAGCAGTGTCTTGTTGTTGT
ZEB1-P1(ChIP-qPCR)	Primer-F: TGGTGGGGTGGGGTCAATTCC
	Primer-R: ACGGTGTCCTTGCTTTGCTTGG
ZEB1-P2(ChIP-qPCR)	Primer-F: ACTCCGGTCACGTTTCAGTTT
	Primer-R: TGCTTCCCACCTCCTTCGAA

ZEB1-P3(ChIP-qPCR)	Primer-F: CTGGAAGGGAAGGGAAGGGAGTC
	Primer-R: TGAGGGGAAGGGCAGGTTTGG
ZEB1-P4(ChIP-qPCR)	Primer-F: TCCCTCGCCCCTCAATTCAAATTC
	Primer-R: CCGCACCTGGTTTACGACACTC
LINC01977-P1 (ChIP-qPCR)	Primer-F: TCCAAGGCGTCAAGGGCAATTC
	Primer-R: CAGGTGGTTCTCATGGACAAGGC
LINC01977-P2(ChIP-qPCR)	Primer-F: GAGAGGAGAGTGGAGCCGTCAG
	Primer-R: ACCGTAAAGGAGTAGGGATGGATCG
LINC01977-P3 (ChIP-qPCR)	Primer-F: TCCTGCTTAGGTGGGAGGTTGAC
	Primer-R: TGGGCCATACATCCATTTGGTTCC
E1 (ChIP-qPCR)	Primer-F: CCTGCCACAGGATGTTGAGAAGTG
	Primer-R: AAACCCACCCTCGTGATAATGAACC
E2 (ChIP-qPCR)	Primer-F: CTCCATTCTCAAGCGAGGACACTG
	Primer-R: GCCACACAGGTCAGATCACAGAAC
E3 (ChIP-qPCR)	Primer-F: GTGCAGGAAGGTGGGAATGGAATG
	Primer-R: GCCCTACCCTGTCATACCCTCTC
E4 (ChIP-qPCR)	Primer-F: CCATACCGAAGAACCGAAGAGCAG
	Primer-R: GGCAGATGTTCCCCAGGCATTG
E5 (ChIP-qPCR)	Primer-F: GTGCAGGAAGGTGGGAATGGAATG
	Primer-R: GCCCTACCCTGTCATACCCTCTC
E6 (ChIP-qPCR)	Primer-F: TTCTTCCTGCCTGTAAAAGCCTCAC
	Primer-R: GGTGGGACTCGGGAACAAAAGTG
CREBBP(Crispr/Cas9) sg#1	Primer-F: GACGACGTCTGGCTCATGTT
	Primer-R: AGGTACCACAGGCAGAGAGG
CREBBP(Crispr/Cas9) sg#2	Primer-F: CAACAGCAGATGAAGCAGCA
	Primer-R: AGGGACAGTTCGCTGGACAG
CREBBP(Crispr/Cas9) sg#3	Primer-F: ATTACAAGCGTGAGCCACCG
	Primer-R: CACCAATGTAGCTAATCAAG
EP300(Crispr/Cas9) sg#1	Primer-F: TTAACAGGCACAAATGTCTA
	Primer-R: CTGCTGTGCTCTGCTGTGAG
EP300(Crispr/Cas9) sg#2	Primer-F: CTCCTGTCTTTGGCTTTTCT
	Primer-R: CCCTAAGTCCAAAATTGTTA
EP300(Crispr/Cas9) sg#3	Primer-F: AGCCTGGATGACAGAGCGAG
	Primer-R: CAGGCCTCTATAAAATACGT
SMAD3(Crispr/Cas9) sg#1	Primer-F: CAGTCACTGGGAGCAGCTCT
	Primer-R: ATCCAATCACCTCCAGATTG
SMAD3(Crispr/Cas9) sg#2	Primer-F: TCACTGGGAGCAGCTCTGCT
	Primer-R: AACCTAATCCAATCACCTCC
SAMD3(Crispr/Cas9) sg#3	Primer-F: AGTCACTGGGAGCAGCTCTG
	Primer-R: TAATCCAATCACCTCCAGAT

Supplementary Table 9. Antibodies, reagents and kits used in this study

Antibodies	Source	Identifier	Application
anti-Acetyl-Histone H3 (Lys27) (D5E4)	Cell Signaling Technology	Cat#8173S, RRID: AB_10949503	IF(1:200), ChIP(1:50)
anti-CBP (D6C5)	Cell Signaling Technology	Cat#7389S, RRID: AB_2616020	IP(1:200), ChIP(1:100) WB(1:1000)
anti-CD3	Biolegend	Cat#317318, RRID: AB_1937212	Cell sorting
anti-CD4	Biolegend	Cat#317416, RRID: AB_571945	Cell sorting
anti-CD8	Biolegend	Cat#344722, RRID: AB_2075388	Cell sorting
anti-CD206 (E6T5J)	Cell Signaling Technology	Cat#24595S, RRID: AB_2892682	IF(1:150)
anti-CD68 (D4B9C)	Cell Signaling Technology	Cat#76437S, RRID: AB_2799882	IF(1:200)
anti-CBP	Abcam	Cat#ab2832, RRID: AB_303342	IHC(1:200)
anti-GAPDH (14C10)	Cell Signaling Technology	Cat#2118S, RRID: AB_561053	WB(1:1000)
anti-HA-tag (C29F4)	Cell Signaling Technology	Cat#3724T, RRID: AB_1549585	WB(1:1000)
anti-Histone H3 (D1H2)	Cell Signaling Technology	Cat#4499T, RRID: AB_10544537	WB(1:1000)
anti-P300 (D2X6N)	Cell Signaling Technology	Cat#54062S, RRID: AB_2799450	ChIP(1:100), IP(1:150) WB(1:1000)
anti-Phospho-SMAD2 (Ser465/467)(E8F3R)	Cell Signaling Technology	Cat#18338T, RRID: AB_2798798	WB(1:1000)
anti-Phospho-SMAD3 (Ser423/425)(C25A9)	Cell Signaling Technology	Cat#9520S, RRID: AB_2193207	WB(1:1000), IHC(1:150), IF(1:150), IP(1:200)
anti-SMAD2 (D43B4)	Cell Signaling Technology	Cat#5339S, RRID: AB_10626777	WB(1:1000)

anti-SMAD2/3 (D7G7)	Cell Signaling Technology	Cat#8685S, RRID: AB_10889933	WB(1:1000)
anti-SMAD3(phospho S423+425) (EP823Y)	Abcam	Cat#ab52903, RRID: AB_882596	IHC(1:200), IP(1:150)
anti-SMAD3(C67H3)	Cell Signaling Technology	Cat#9523S, RRID: AB_2193182	WB(1:800), ChIP(1:100)
anti-SMAD4 (D3R4N)	Cell Signaling Technology	Cat#46535S, RRID: AB_2736998	WB(1:1000), IP(1:150)
anti-ZEB1	Proteintech	Cat#21544-1-AP, RRID:AB_10734325	WB(1:800), IHC(1:400)
anti-β-Actin (8H10D10)	Cell Signaling Technology	Cat#3700S, RRID: AB_2242334	WB(1:1000)
anti-FLAG-Tag	Thermo Fisher	Cat#MA1-91878, RRID: AB_1957945	WB(1:1000)
anti-cyclinD1	Abcam	Cat#ab134175, RRID: AB_2750906	WB(1:1000)
anti-cyclinE1	Abcam	Cat#ab33911, RRID:AB_731787	WB(1:1000)
anti-E-cadherin	Cell Signaling Technology	Cat#14472S, RRID:AB_2728770	WB(1:1000) IHC(1:400)
anti-N-cadherin	Cell Signaling Technology	Cat#13116S, RRID:AB_2687616	WB(1:1000)
anti-SNAIL	Cell Signaling Technology	Cat#3895S, RRID:AB_2191759	WB(1:1000)
anti-cleaved Caspase3	Cell Signaling Technology	Cat#9664S, RRID:AB_2070042	IHC(1:300)
anti-Ki-67	Cell Signaling Technology	Cat#2586S, RRID:AB_2160343	IHC(1:300)
anti-PCNA	Cell Signaling Technology	Cat#13110S, RRID:AB_1083636	IHC(1:300)
**Reagents**	**Source**	**Identifier**	**Application**
SGC-CBP30	ApexBio	Cat#A4491	in vitro and in vivo assays

JQ1	ApexBio	Cat#A1910	in vitro assays
SB431542	ApexBio	Cat#A8249	in vitro assays
SIS3	ApexBio	Cat#B6096	in vitro assays
TGF-β	ApexBio	Cat#P1039	in vitro assays
Lipofectamine 3000	Invitrogen	Cat#L3000-075	DNA and lncRNA-ASO transfection
Lipofectamine CRISPRMAX	Invitrogen	Cat#CMAX00008	Crispr/Cas9 system
DMSO	Solarbio	Cat#D8371	*in vitro* assays
TRIzol	Invitrogen	Cat#15596018	RNA extraction
Mayer's hematoxylin solution	Solarbio	Cat#G1080	RNAScope and IHC assay
PowerUp^{TM} SYBR Green Master Mix	Thermo Fisher	Cat#00775499	qRT-PCR
Pierce IP lysis buffer	Thermo Fisher	Cat#87788	RNA pull-down assay
Cremophor	Selleck Chemicals	Cat#S6828	*In vivo* assays
Cas9 protein	Thermo Fisher	Cat#A50577	Crispr/Cas9 system
Ambion RNase cocktail	Thermo Fisher	Cat#AM2286	Dependency assay with co-IP
Phanta Max Master Mix (Dye Plus)	Vazyme	CatP525-02	High fidelity PCR amplification
**Kits**	**Source**	**Identifier**	**Application**
RNAScope 2.5 High Definition Red Reagent Assay Kit	Advanced Cell Diagnostics	Cat#322350	RNAScope assay
EZ-Magna RIP Kit	Merck Millipore	Cat#17-701	RIP assay
EZ-Magna Nuclear RIP Kit (Native)	Merck Millipore	Cat#17-10523	RIP assay
EdU DNA Proliferation Kit	KeyGene	Cat#KGA331-100	EdU assay
RNeasy FFPE Kit	Qiagen	Cat#73504	RNA extraction from FFPE
Primer Script RT reagent Kit	Takara	Cat#RR036A	qRT-PCR
Dual-Luciferase Assay Kit	Promega	Cat#E1910	Dual-luciferase reporter assays
RNAmax-T7 transcription Kit	RiboBio	Cat#C11001-2	In vitro RNA transcription
Pierce RNA 3' End	Thermo	Cat#20163	Biotin-lable RNA

Desthiobiotinylation Kit	Fisher		probes for RNA pull-down assay
Pierce Magnetic RNA-Protein Pull-Down Kit	Thermo Fisher	Cat#20164	RNA Pull-down
ChIP-IT Express Kit	Active Motif	Cat#53008	ChIP assays
PARIS protein and RNA isolation Kit	Invitrogen	Cat#AM1921	Subcellular fractions isolation
PCR Array (Human-EMT)	Wcgene Biotech	Cat#WC-MRNA0053-H	qRT-PCR detection for EMT associated genes
Human TGF-beta1 ELISA Kit	FCMACS Biotech	Cat#FMS-ELH038	ELISA detection for TGF-beta1
Genomic DNA extraction kit	TIANGEN	Cat#DP304	DNA extraction
Pierce Co-Immunoprecipitation Kit	Thermo Fisher	Cat#26149	co-IP assays
